# Transition-metal-free controlled polymerization for poly(*p*-aryleneethynylene)s†
†Electronic supplementary information (ESI) available: Experimental details and characterizations of new compounds and polymers. See DOI: 10.1039/c4sc02872d



**DOI:** 10.1039/c4sc02872d

**Published:** 2014-10-29

**Authors:** Takanobu Sanji, Asahi Motoshige, Hideaki Komiyama, Junko Kakinuma, Rie Ushikubo, Satoru Watanabe, Tomokazu Iyoda

**Affiliations:** a Iyoda Supra-Integrated Material Project (iSIM) , Exploratory Research for Advanced Technology (ERATO) , Japan Science and Technology Agency (JST) , Tokyo Institute of Technology , 4259-S2-3 Nagatsuta, Midori-ku , Yokohama 226-8503 , Japan . Email: sanji.t.aa@m.titech.ac.jp

## Abstract

A transition-metal-free controlled polymerization for the attainment of poly(*p*-aryleneethynylene)s is developed. The polymerization of 1-pentafluorophenyl-4-[(trimethylsilyl)ethynyl]benzene with a catalytic amount of fluoride anions proceeds in a chain-growth-like manner to afford polymers with controlled molecular weights and low polydispersity indexes. The mechanism involves a pentacoordinated fluorosilicate as a key intermediate. The anionic “living” nature of this process is applied to block copolymerization and also surface-terminated polymerization.

## Abstract 2

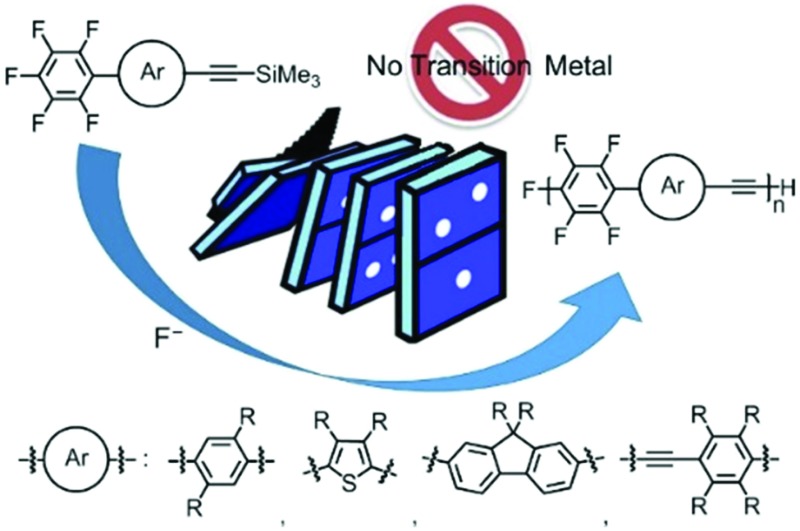
A catalytic amount of fluoride anions promoted the polymerization of 1-pentafluorophenyl-4-[(trimethylsilyl)ethynyl]benzene, providing a high-molecular-weight polymer in a chain-growth-like manner.

## Introduction

Among the most extensively studied families of molecular optoelectronic materials, such as organic field-effect transistors (FETs) and organic electroluminescent (EL) devices, poly(*p*-aryleneethynylene)s (PAEs) are one of the most important types of materials.^1^ The polymers are commonly prepared by polycondensations using Sonogashira- or Stille-type cross couplings^2^ or by an alkyne metathesis.^3^ In all cases, however, the molecular weight (MW) and polydispersity index (PDI) of the polymers are not controlled because the polycondensations proceed by a step-growth mechanism. In contrast to these conventional step-growth polycondensations, chain-growth polymerizations have been demonstrated recently, but they require transition-metal catalysts.^4^ There remains a need to achieve additional synthetic methods for well-defined polymers with control of the MW, the polydispersity, modification of the end group, and also block copolymerization, which could offer new architectures and materials.^5^

Here, we describe a transition-metal-free controlled polymerization for the attainment of PAEs. The polymerization proceeds in a chain-growth-like manner to afford the polymers with controlled MWs and low PDIs. This could be an alternative synthetic method for well-controlled PAEs.

## Results and discussion

**Scheme 1:**
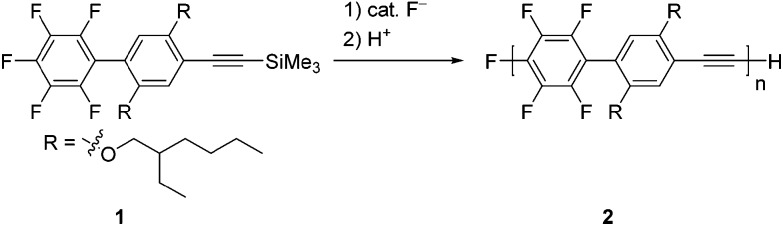
Polymerization of **1**.

We designed 1-pentafluorophenyl-4-[(trimethylsilyl)ethynyl]benzene **1** as a monomer and examined its polymerization with a catalytic amount of fluoride anions (Scheme 1). This is because fluoride anions were found to catalyze silylacetylene activation for subsequent reaction with a number of electrophiles,^6,7^ and also because regioselective S_N_Ar reactions of perfluoroaryl groups with nucleophiles^8,9^ are well studied. Very recently, we demonstrated the transition-metal-free polymerization of 2-perfluoroaryl-5-trimethylsilylthiophenes promoted by fluoride anions to afford polymers with controlled MWs and low PDIs.^10^ In contrast, the reported polycondensation of hexafluorobenzene and 1,4-bis[(trimethylsilyl)ethynyl]benzene was not controlled.^11^ Recently, Bielawski *et al.* reported a controlled Pd catalyzed transfer polycondensation for poly(*p*-phenyleneethynylene).^12^

A catalytic amount of fluoride anions smoothly promoted the polymerization of **1**. Table 1 summarizes the polymerization results. For example, the reaction of **1** with 5 mol% tetrabutylammonium fluoride (TBAF)^13^ as a fluoride anion source in tetrahydrofuran (THF) at room temperature for 2 h led to poly(*p*-tetrafluorophenylene-phenylene-ethynylene) **2** with a number-averaged MW (*M*_n_) of 4400 and a PDI of 1.31 in an 81% isolated yield (entry 2). The MWs measured by size-exclusion chromatography (SEC)-multi-angle light scattering (MALS) were almost the same as those measured by SEC. When the polymerization of **1** was examined with respect to varying the mol% of TBAF, the MW of the polymer linearly increased with an increasing monomer to TBAF ratio (entries 1–4), while the PDIs were relatively low (≤2). Addition of **1** to a solution of TBAF also afforded the polymer (entry 5). However, tetramethylammonium fluoride was not a good initiator because some remained undissolved in THF (entry 6). Potassium fluoride or cesium fluoride in the presence of 18-crown-6 or cryptand[2.2.2] did not give a good result (entries 7–9). Tetrabutylammonium difluorotriphenylsilicate was not effective for the polymerization of **1** (entry 10).

**Table 1:** Polymerization of **1** catalyzed by fluoride anions^*a*^

Entry	F^–^ (mol%)	Reaction conditions	Yield (%)	*M*_n_ ^*b*^ ^,^ ^*c*^	PDI^*b*^ ^,^ ^*c*^
1	Bu_4_NF (TBAF) (10)	THF, rt, 2 h	34	3950 (3600)	1.26 (1.24)
2	Bu_4_NF (TBAF) (5)	THF, rt, 2 h	81	4400 (4500)	1.31 (1.31)
3	Bu_4_NF (TBAF) (2)	THF, rt, 2 h	52	7400 (8900)	1.68 (1.68)
4	Bu_4_NF (TBAF) (1)	THF, rt, 2 h	82	11 500 (14 500)	2.11 (1.57)
5	Bu_4_NF (TBAF) (5)	THF, rt, 2 h^*d*^	48	7600	1.72
6	Me_4_NF (5)	THF, rt, 2 h	71	4500	4.15
7	KF (5)/18-C-6 (10)	THF, 80 °C, 20 h	1	1400	1.08
8	CsF (5)/18-C-6 (10)	Toluene, 80 °C, 20 h	75	13 700	3.88
9	KF (5)/cryptand[2.2.2] (10)	THF, rt, 2 h	0	—	—
10	Bu_4_N^+^[Ph_3_SiF_2_]^–^ (5)	THF, rt, 2 h	27	2500	1.48

^*a*^All reactions were run with [**1**] = 0.3 mmol in 5 mL solvent.

^*b*^The number-average MW (*M*_n_) and PDI were determined by SEC using polystyrene standards.

^*c*^The numbers in parentheses are the weight-averaged MW (*M*_w_) and the PDI determined by SEC-MALS.

^*d*^A THF solution of **1** was added to a TBAF solution.

The obtained polymer has a structure with high regioregularity, as demonstrated by ^1^H, ^13^C, and ^19^F NMR analyses (Fig. S1 in the ESI†). The ^1^H NMR spectrum of **2** shows major signals at around 0.8, 1.4, and 4.0 ppm owing to the side chain on the phenylene units, along with a small signal at 3.7 ppm arising from the ethynyl group at the polymer end. In the ^19^F NMR spectrum of the polymer, two strong signals and three weak signals were found, which are assigned to the 1,4-tetrafluorophenylene units in the main chain and the pentafluorophenyl group at the polymer end, respectively. Thus, the NMR spectra are consistent with a high regioregularity for the polymer main chain, indicating that the polymerization process itself must be highly regioselective. In addition, because the polymer ends are designated as the pentafluorophenyl and the ethynyl groups, the integral ratio of the peaks from the side chain on the main chain and the end group in the ^1^H and ^19^F NMR spectra provide a *M*_n_ of *ca.* 5000, which is in reasonable agreement with the MW estimated by SEC (Table 1, entry 2). Furthermore, matrix-assisted laser desorption ionization time-of-flight (MALDI-TOF) mass spectra also indicate that the polymer has pentafluorophenyl and ethynyl groups at its ends (Fig. S2 in the ESI†).

**Fig. 1:**
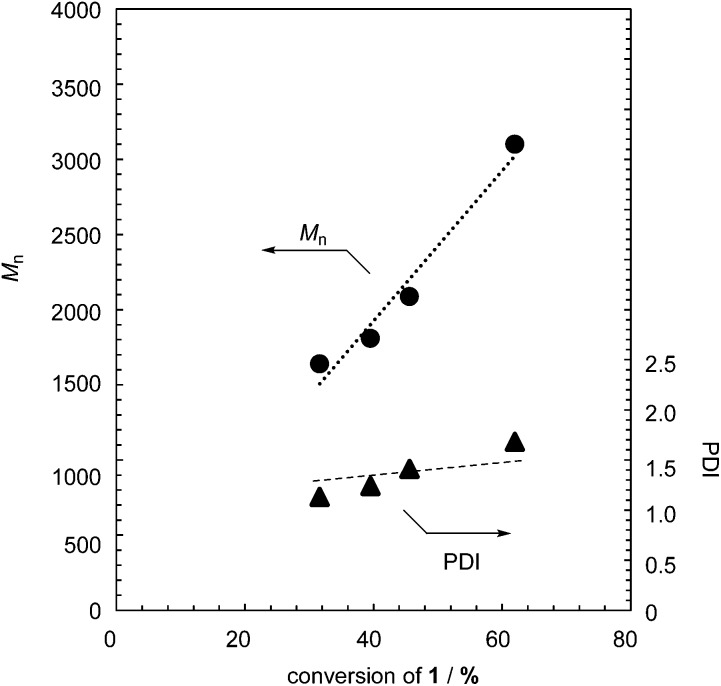
*M*_n_ and PDI of formed polymer **2** as functions of monomer conversion in the polymerization of **1** with 2.5 mol% TBAF.

The controlled MWs and relatively low PDIs found in Table 1 indicate that the polymerization proceeds in a chain-growth-like manner under the specified conditions. To provide further evidence for this, we monitored the polymerization of **1** with 2.5 mol% TBAF as a function of monomer conversion. Fig. 1 shows the *M*_n_ and PDI as functions of monomer conversion. The polymerization of **1** was fast, with the conversion of **1** being up to 50% after a few minutes (Table S1 and Fig. S3 in the ESI†). As shown in Fig. 1, the linear relationship between *M*_n_ and monomer conversion, and the relatively low PDI, confirm the chain-growth-like process.

**Scheme 2:**
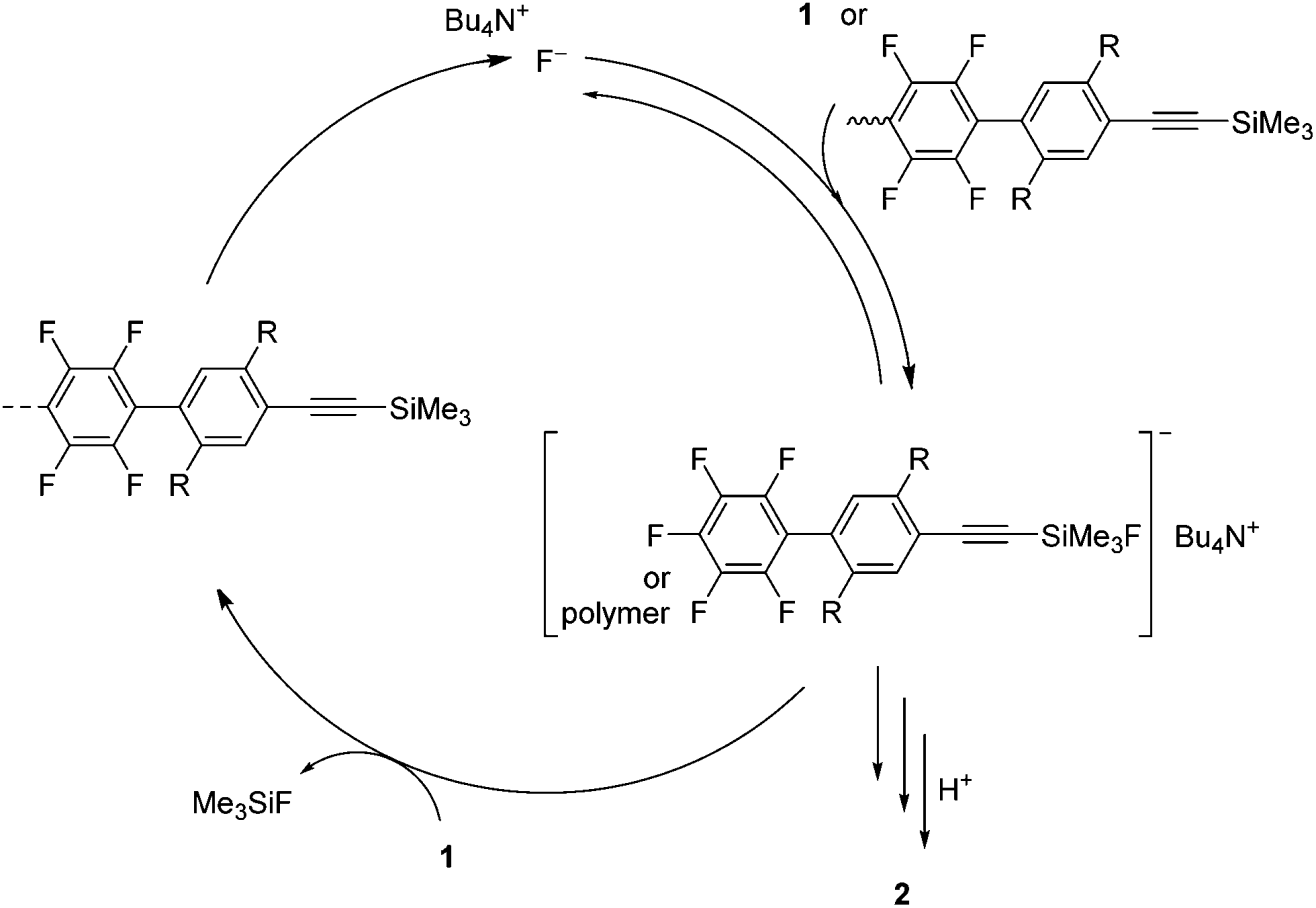
Possible polymerization mechanism of **1**.

Scheme 2 shows a possible polymerization mechanism. In the initial reaction step, a fluoride anion attacks the trimethylsilyl group of **1** to form a pentacoordinate silicate. The silicate is quite reactive and could regioselectively attack the 4-position of the pentafluorophenyl group of **1** to reproduce a fluoride anion. The fluoride anion would then transfer intramolecularly to the trimethylsilylethynyl group at the polymer end, where there may be an anion–π interaction that produces an associated pair.^14^ Then, the fluoride anion catalyzes the polymerization to give polymer **2**. In the polymerization, the reactivity of the trimethylsilyl group and/or the silicate at the polymer end is changed by replacing the 4-position of the pentafluorophenyl group of **1**. Watson *et al.* discussed the change in reactivity in the polymerization of 1,4-bis[(trimethylsilyl)ethynyl]benzene and hexafluorobenzene by substitution with fluoride anions.^11^ Further description of the polymerization mechanism requires further studies.

**Scheme 3:**
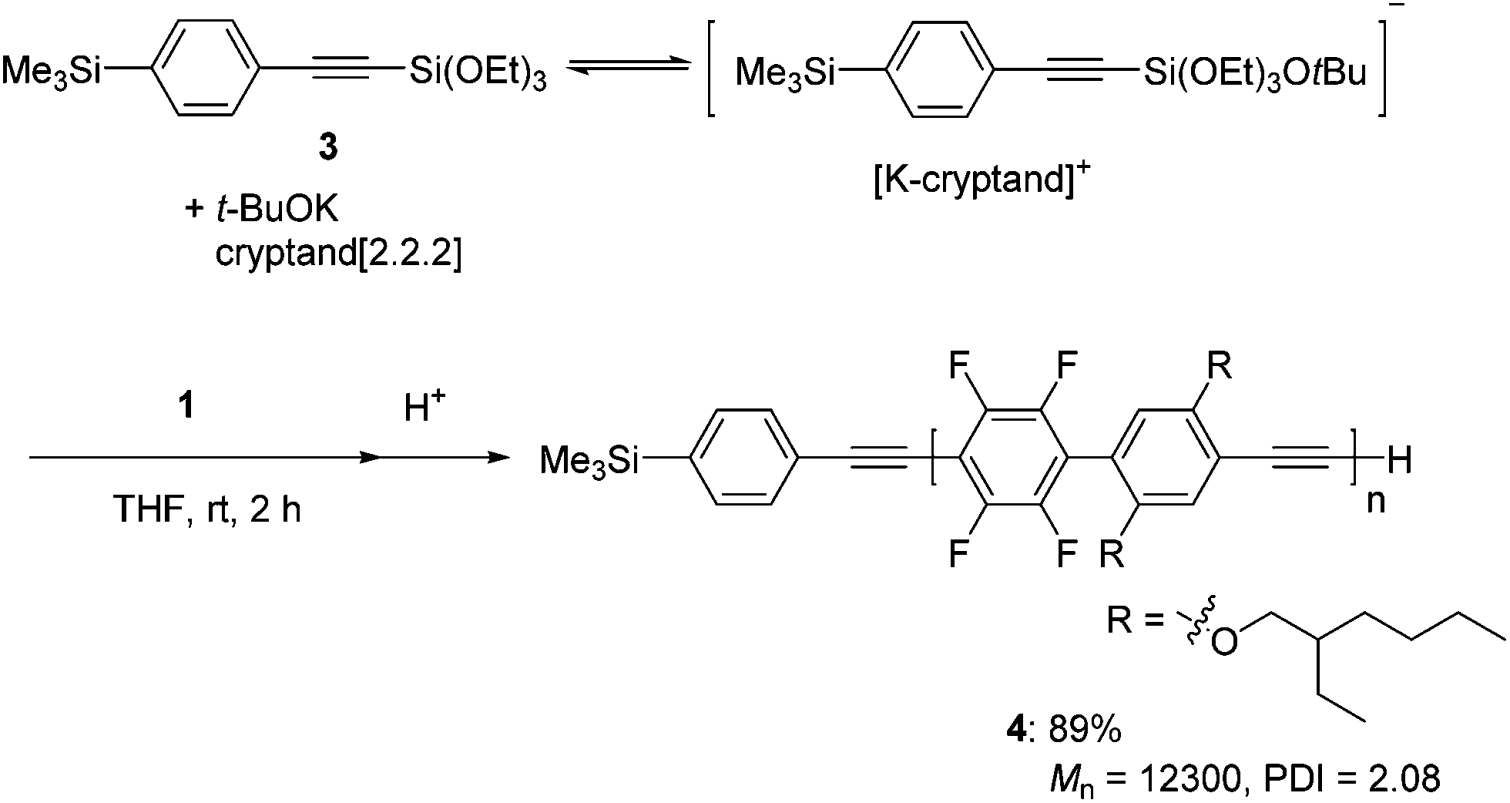
Polymerization of **1** with a pentacoordinated alkynylsilicate prepared by the reaction of **3** and potassium *t*-butoxide.

According to the polymerization mechanism, all of the propagating polymer chains contain reactive pentacoordinate silicate at a chain terminus. Indeed, a pentacoordinated alkynylsilicate, which is prepared *in situ* by the reaction of 1-(4-trimethylsilyl)phenyl-2-(triethoxysilyl)acetylene **3** and potassium *t*-butoxide,^15^ efficiently initiated the polymerization of **1** in the presence of cryptand[2.2.2] to give polymer **4** with a *M*_n_ of 12 300 and a PDI of 2.08 in 89% yield (Scheme 3).^16^ The ^1^H NMR spectrum of **4** shows peaks at around 0.3 and 3.4 ppm arising from the trimethylsilyl and ethynyl groups at the polymer ends, respectively, where the observed integral ratio is in good agreement with the calculated value (calcd for –SiMe_3_/–C≡CH 9.0, found 8.8) within the experimental errors (Fig. S4 in the ESI†).^17^

**Scheme 4:**
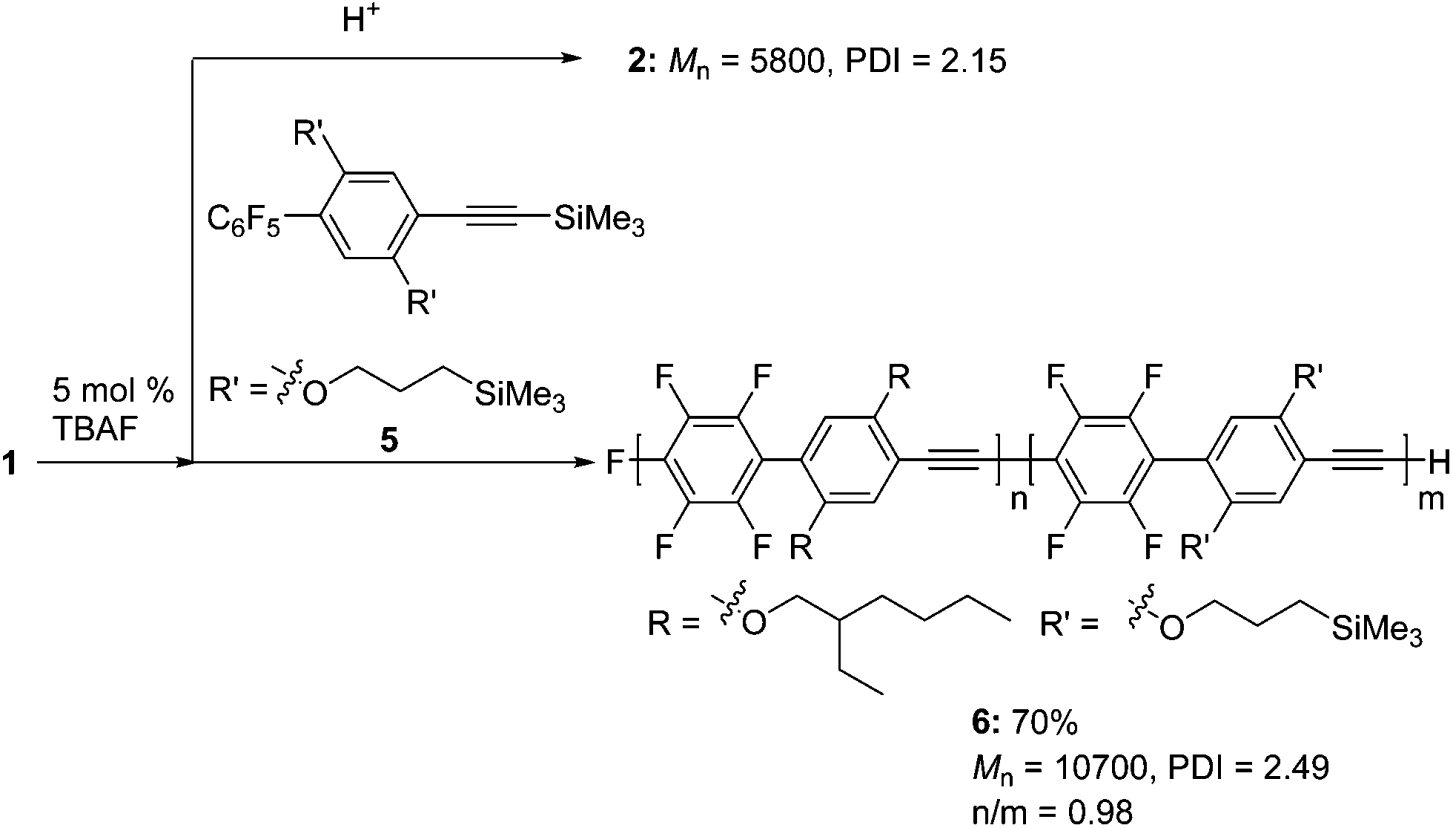
Synthesis of block copolymer **6**.

**Fig. 2:**
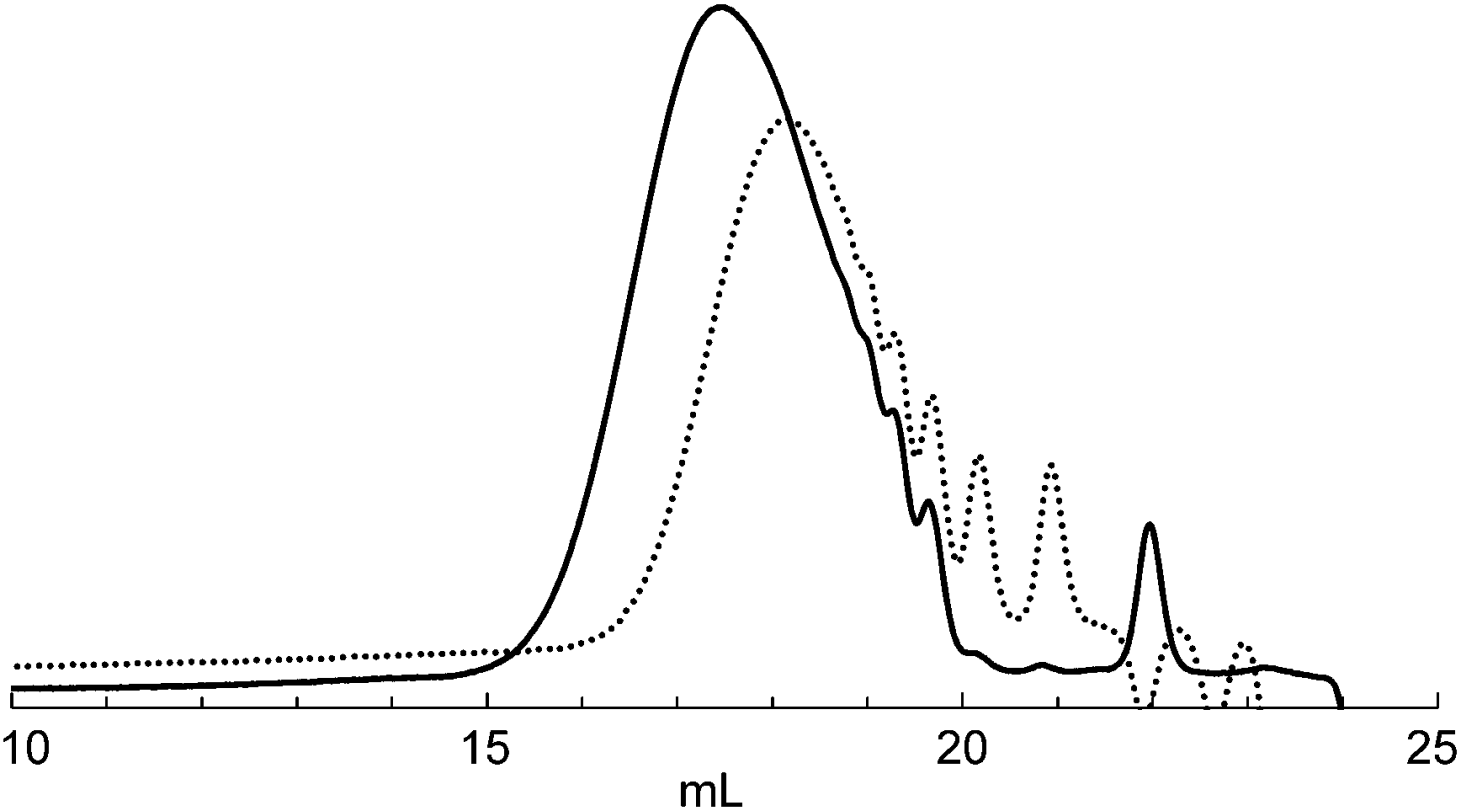
SEC curves (RI) obtained for the first polymerization of **1** (polymer **2**, dotted line) and following sequential addition of **5** (polymer **6**, solid line) after the first polymerization of **1**.

Next, we demonstrated the anionic “living” character of this polymerization end by applying it to the synthesis of a well-defined block copolymer (Scheme 4). After polymerization of **1** with a catalytic amount of TBAF, part of the mixture was studied to analyze the MW of the first block, and then a solution of **5** was added to the reaction mixture to give block copolymer **6**.^18^ As shown in Fig. 2, the SEC curve of the polymer obtained at the end of the reaction was shifted toward a higher MW compared to that of the first polymerization. The first/second block ratio (n/m) of **6** was found to be 0.98 by ^1^H NMR analysis (Fig. S6 in the ESI†).

**Scheme 5:**
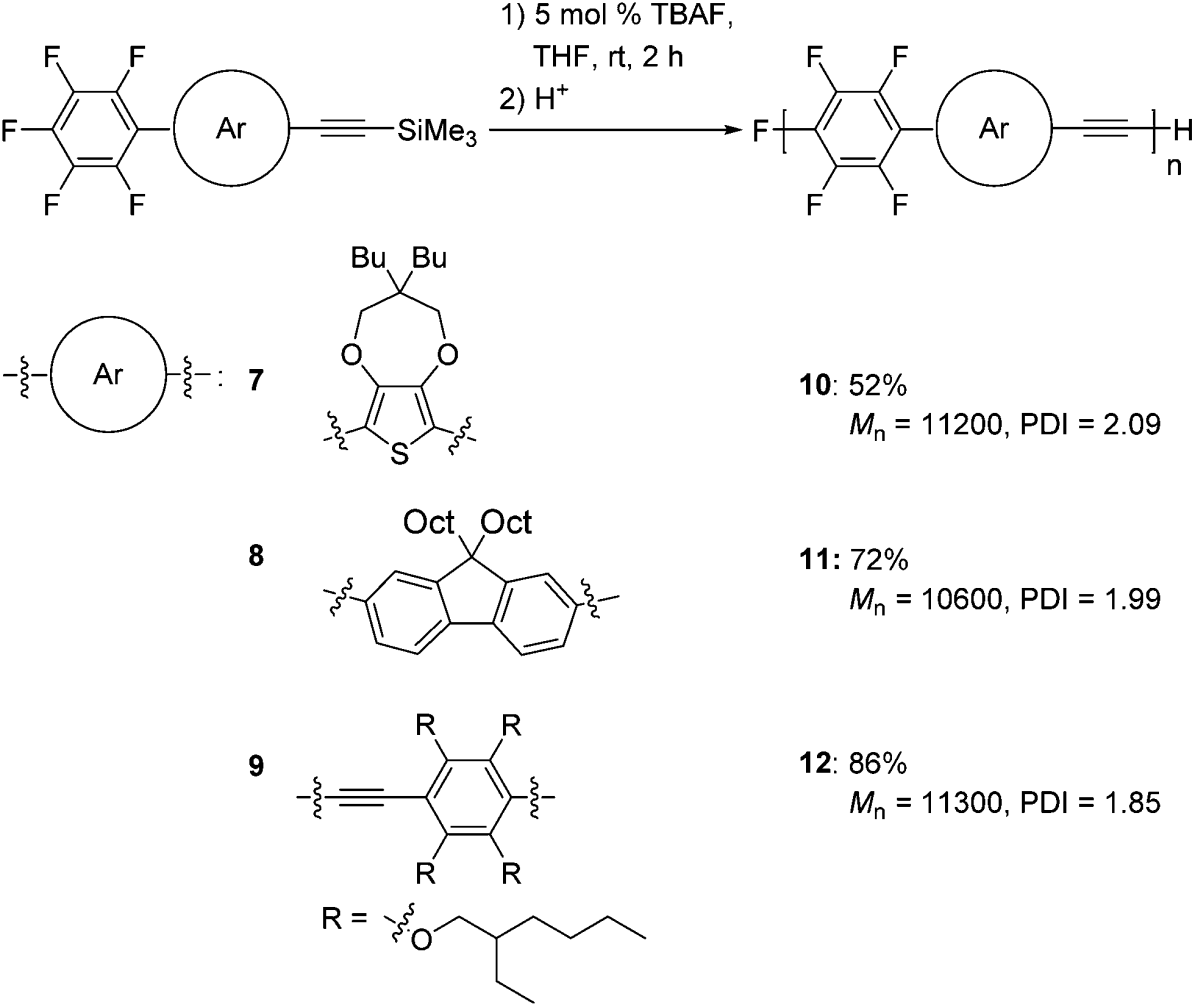
Polymerizations of **7–9**.

We also extended our method to the polymerization of a variety of aromatic monomers, **7–9**, which possess trimethylsilylethynyl and pentafluorophenyl groups (Scheme 5). The reaction of 2-pentafluorophenyl-5-[(trimethylsilyl)ethynyl]thiophene **7** with 5 mol% TBAF gave polymer **10** with a *M*_n_ of 11 200 and a PDI of 2.09 in a moderate yield. Fluorene-incorporated **8** was polymerized by addition of 5 mol% TBAF to give polymer **11** with a *M*_n_ of 10 600 and a PDI of 1.99 in a good yield. A phenyleneethynylene monomer **9** was also polymerized to afford polymer **12** with a *M*_n_ of 11 300 and a PDI of 1.85. The structures of these polymers are highly ordered, as demonstrated by NMR analyses (Fig. S7–S9 in the ESI†), indicating that the polymerizations also proceed with high regioselectivities.

**Scheme 6:**
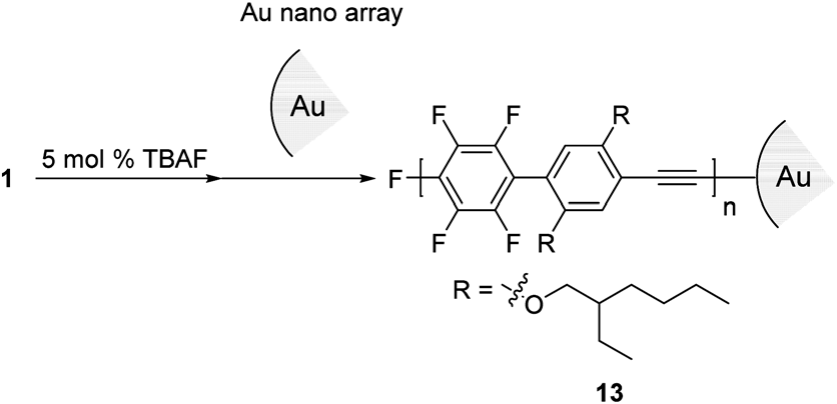
Surface termination with a Au nanoparticle array for the polymerization of **1**.

**Fig. 3:**
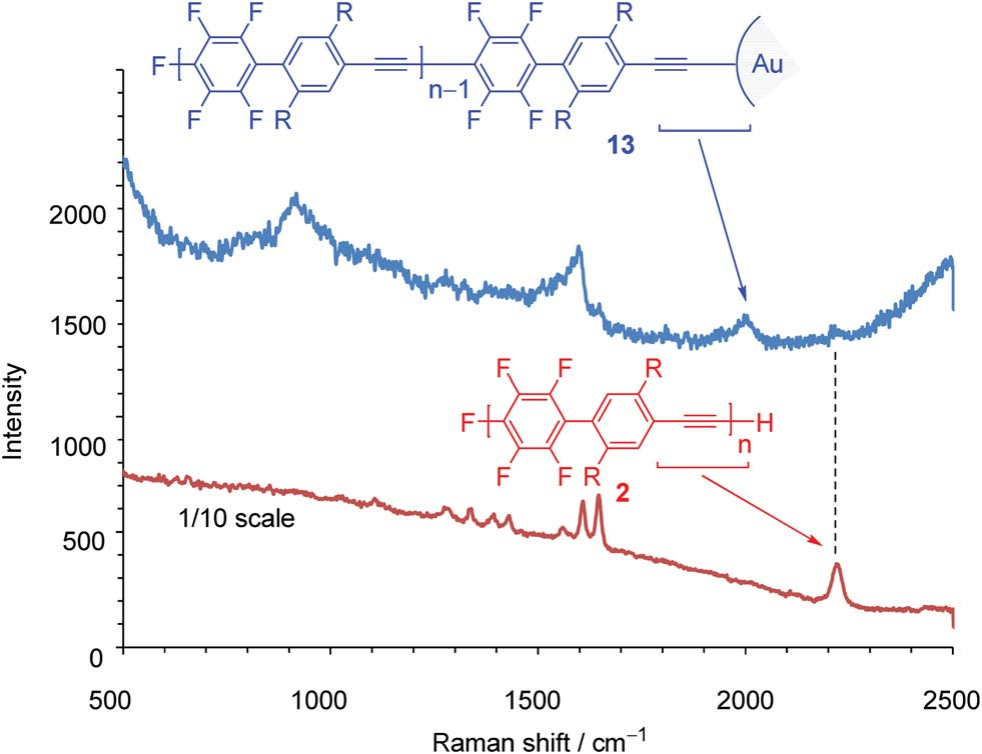
Raman spectra of **2** (film, red) and **13** (blue).

Next, we turned our attention to exploring the surface-terminated polymerization of **1**. Au surfaces of nanometer-sized particles are an ideal substrate for this study because of their well-established chemistry and their features which could be exploited for molecular device applications.^19^ Surface-terminated polymerization was accomplished by the addition of a surface-enhanced Raman scattering (SERS)-active Au nanoparticle (*φ* 15 nm) array^20^ to a polymerization mixture of **1** (Scheme 6). After standing for 1 h, the plate was washed with THF and then analyzed by Raman spectroscopy. SEC analysis of the polymerization mixture shows that the resulting polymer, **13**, has a *M*_n_ of 4700 and a PDI of 1.79. The *ν*_C≡C_ stretching region in the SERS spectra is particularly informative. The Raman spectrum of **2** shows a signal at around ∼2200 cm^–1^ owing to the ethynyl groups in the polymer main chain (Fig. 3). On the other hand, in the Raman spectrum of **13**, a broad and downshifted signal is observed at 2050 cm^–1^. The red-shift of ∼150 cm^–1^ indicates a strong interaction with the Au substrate, which is in agreement with previous reports.^21^ Thus, the ethynyl group of the polymer end is attached to the gold substrate *via* covalent Au–C≡C bonds. Although this is a preliminarily demonstration, it represents a possible step for molecular device applications. Further details will be reported.

## Conclusions

We have developed a transition-metal-free controlled synthesis of PAEs promoted by fluoride anions. The polymerization proceeds in an anionic chain-growth-like manner to afford PAEs with controlled MWs and relatively low PDIs. The polymerization end is active and affords a block copolymer. We also demonstrated the synthesis of a surface-terminated PAE on a Au nanoparticle array. We expect that this concept can be extended to prepare other well-controlled conjugated polymers, providing new opportunities in optoelectronics and other applications. Further study along this line is currently in progress.
