# Supplementary material for: Transition-metal-free controlled polymerization for poly(p-aryleneethynylene)s
Source: Chem Sci. 2014 Oct 29;6(1):492–6. doi: 10.1039/c4sc02872d (PMC5592747; doi:10.1039/c4sc02872d)
Supplement: Supplementary file 1 [file SC-006-C4SC02872D-s001.pdf]

Electronic Supplementary Material (ESI) for Chemical Science.
This journal is © The Royal Society of Chemistry 2014

Electronic Supplementary Information (ESI) for

Transition-metal-free controlled polymerization for poly(*p*-aryleneethynylene)s

Takanobu Sanji,* Asahi Motoshige, Hideaki Komiyama, Junko Kakinuma, Rie Ushikubo, Satoru Watanabe and Tomokazu Iyoda
Iyoda Supra-Integrated Material Project (iSIM), Exploratory Research for Advanced Technology (ERATO), Japan Science and Technology Agency (JST), and Tokyo Institute of Technology, 4259-S2-3 Nagatsuta, Midori-ku, Yokohama 226-8503, Japan. E-mail: sanji.t.aa@m.titech.ac.jp

Experimental section

Measurements. ^{1}H, ^{13}C, ^{19}F, and ^{29}Si NMR spectra were recorded using a Bruker Biospin AVANCE 400 FT-NMR spectrometer at 400, 100, 376, and 79.5 MHz, respectively. ^{1}H and ^{13}C chemical shifts were referenced to solvent residues. Mass and FAB mass spectra were obtained with a JEOL JMS-700 mass spectrometer. SEC was performed on a JASCO HPLC LC-2000Plus equipped with SHODEX LF804 (×2) columns with THF as an eluent. SEC-multi-angle light scattering (SEC-MALS) experiments were also performed on a JASCO instrument equipped with a Wyatt Technology DAWN HELEOS II 18-angle laser light scattering detector (λ = 658 nm). The analysis was carried out using Astra v.5.3.4 software. The scattering angle ranges from 15° to 165°. The dn/dc of **2**, estimated by Wyatt Technology Optilab T-rEX, was 0.175. Matrix-assisted laser desorption ionization time-of-flight (MALDI-TOF) mass spectra were recorded on a Shimadzu/Kratos AXIMA-CFR plus in the linear ion mode. Dithranol (1,8-dihydroxy-9[10H]-anthracenone) was used as the matrix for the MALDI-TOF mass measurements. Raman spectra were recorded using a HoloLab 5000R series Raman system (Kaiser Optical Systems Inc., USA). The excitation wavelength was 532 nm with 0.25 mW power on the sample.

Materials. All manipulations involving air and moisture-sensitive compounds were carried out under atmosphere of dry argon. All solvents and reagents were of reagent quality, purchased from commercial sources and used without further purification. Silica gel for column chromatography was purchased from Merck (Silica Gel 60, 0.0040-0.063 nm). TLC plates were purchased from Merck (Silica Gel60F254 TLC plate).

Synthetic procedures and compounds characterization

Synthesis of 1,4-(bis(2-ethylhexyloxy)-2-pentafluorophenyl)-5-(trimethylsilylethynyl)benzene 1

Route 1

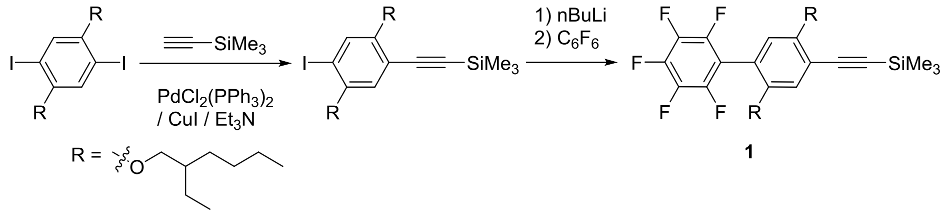

(S1)

1,4-Di(2-ethylhexyloxy)-2-iodo-5-(trimethylsilylethynyl)benzene was prepared by the reported procedure.[S1]

Synthesis of 1,4-(di(2-ethylhexyloxy)-2-pentafluorophenyl)-5-(trimethylsilylethynyl)benzene 1

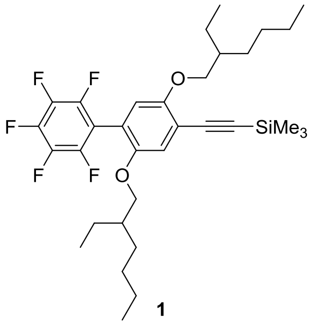

To 1,4-di(2-ethylhexyloxy)-2-iodo-5-(trimethylsilylethynyl)benzene (2.85 g, 5.12 mmol) in ether (60 mL) was added a hexane solution of 1.58 M n-butyllithium (4.0 mL, 6.32 mmol) at −78 °C for a few minutes. The mixture was stirred at the temperature for 1 h. Hexafluorobenzene (5.25 g, 28.2 mmol) was added to the mixture at 0 °C at once and then the mixture was stirred at room temperature overnight. To the mixture was added water and the solution was extracted with hexane. The organic layer was washed with a saturated aqueous solution of NH_4Cl and NaCl and dried over anhydrous $MgSO_4$. After filtration and removal of the solvent, the residue was purified with chromatography (SiO_2, hexane/toluene = 20/1) to give **1** (2.15 g, 3.60 mmol, 70%). A pale yellow oil: ^{1}H NMR ($CDCl_3$, 400 MHz) δ 0.33 (s, 9H), 0.87-1.02 (m, 12H), 1.27-1.82 (m, 18H), 3.84-3.96 (m, 4H), 6.96 (s, 1H), 7.11 (s, 1H); ^{13}C{^{1}H} NMR ($CDCl_3$, 100 MHz) δ −0.1, 11.0. 11.3, 13.9, 14.1, 23.0, 23.1, 29.0, 29.2, 30.5, 30.6, 39.4, 39.7, 71.3, 72.0, 99.7, 100.8, 115.0, 115.7, 116.4, 116.8, 125.3, 128.2, 129.0. 150.3, 154.4; ^{19}F{^{1}H} NMR ($CDCl_3$, 376 MHz) δ −163.6 (ddd, J_F = 21, 21, 8.2 Hz, 2F), −156.0 (t, J_F = 21 Hz, 1F), −139.7 (td, J_F = 21, 8.2 Hz, 2F); ^{29}Si{^{1}H} NMR ($CDCl_3$, 79.5 MHz) δ −17.6; HRMS (FAB, matrix = 3-nitrobenzyl alcohol, NBA) calcd for $C_{33}H_{45}F_5O_2SSi$ ($[M]^+$) *m*/*z* 596.7966, found 596.3095.

Route 2

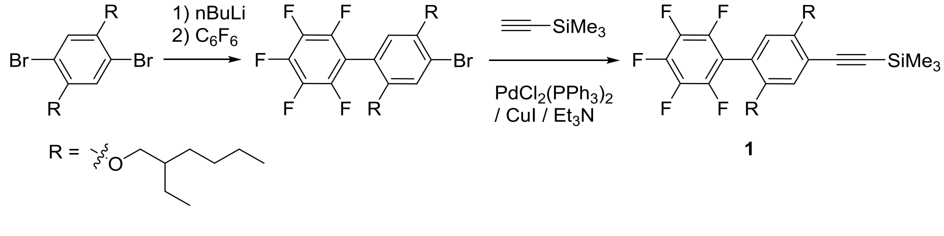

(S2)

S1 S. Kang, R. J. Ono and C. W. Bielawski, *J. Am. Chem. Soc.*, 2013, **135**, 4984.

2,5-Dibromo-1,4-di(2-ethylhexyloxy)benzene was prepared by the reported procedure.[S2]

Synthesis of 2-bromo-1,4-di(2-ethylhexyloxy)-3-pentafluorophenylbenzene

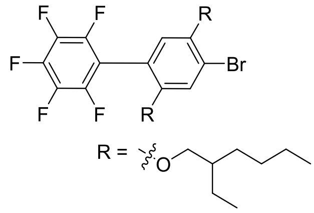

To a ether solution (100 mL) of 2,5-dibromo-1,4-di(2-ethylhexyloxy)benzene (8.77 g, 17.8 mmol) was added a hexane solution of 1.58 M n-butyllithium (12.5 mL, 19.8 mmol) at −78 °C for 10 min. After stirring the mixture at the temperature, hexafluorobenzene (8.07 g, 43.4 mmol) was added to the mixture at one portion, and then the mixture was stirred at room temperature overnight. To the mixture was added a saturated aqueous solution of $NaHCO_3$ and the resulting mixture was extracted with hexane. The organic layer was washed with a saturated aqueous solution of NaCl and dried over anhydrous $MgSO_4$. After filtration and removal of the solvent, the residue was purified with chromatography (SiO_2, hexane) to give the title compound (7.01 g, 12.2 mmol, 68%). A colorless oil; ^1H NMR ($CDCl_3$, 400 MHz) δ 0.87-1.01 (m, 12H), 1.26-1.63 (m, 17H), 1.81 (septet like m, 1H), 3.84-3.91 (m, 4H), 6.64 (s, 1H), 7.27 (s, 1H); $^{13}C\{^1H\}$ NMR ($CDCl_3$, 100 MHz) δ 11.0, 11.1, 13.9, 14.0, 22.94, 23.0, 23.8, 29.1, 30.5, 39.4, 39.6, 71.6, 72.6, 112.1, 112.3, 112.5, 114.5, 114.8, 116.4, 117.6, 136.1, 136.3, 138.7, 138.8, 138.9, 143.1, 143.1, 145.5, 145.6, 145.6, 149.8, 151.0; $^{19}F\{^1H\}$ NMR ($CDCl_3$, 376 MHz) δ −163.4 (ddd, J_F = 21, 21, 8.1 Hz, 2F), −155.8 (t, J_F = 21 Hz, 1F), −139.8 (td, J_F = 21, 8.1 Hz, 2F); HRMS (FAB, matrix = 3-nitrobenzyl alcohol, NBA) calcd for $C_{28}H_{36}BrO_2F_5$ ($[M]^+$) *m*/*z* 579.4887, found 578.1821.

Synthesis of 1,4-di(2-ethylhexyloxy)-2-pentafluorophenyl-5-(trimethylsilylethynyl)benzene 1

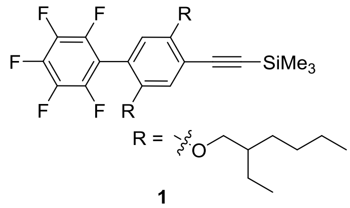

1

To 2-bromo-1,4-di(2-ethylhexyloxy)-3-pentafluorophenylbenzene (6.74 g, 11.63 mmol), $PdCl_2(PPh_3)_2$ (205 mg, 0.29 mmol), and CuI (110 mg, 0.58 mmol) in THF/*i*-Pr_2NH (30 mL, v/v = 2/1) was added trimethylsilylacetylene (2.45 g, 250 mmol) at room temperature. The mixture was stirred at the temperature for 10 min and then at 60 °C overnight. To the mixture was added a saturated aqueous solution of NH_4Cl and the resulting mixture was extracted with hexane. The organic layer was washed with a saturated aqueous solution of NaCl and dried over anhydrous $MgSO_4$. After filtration and removal of the solvent, the residue was purified with chromatography (SiO_2, hexane/toluene = 20/1) to give **1** (4.52 g, 7.57 mmol, 65%). The spectra was the same as

---

S2 A. P. Monkman, L.-O. Pålsson, R. W. T. Higgins, C. Wang, M. R. Bryce, A. S. Batsanov and J. A. K. Howard, *J. Am. Chem. Soc.*, 2002, **124**, 6049.

mentioned above.

Polymerization of 1,4-di(2-ethylhexyloxy)-2-pentafluorophenyl-5-(trimethylsilylethynyl)benzene 1

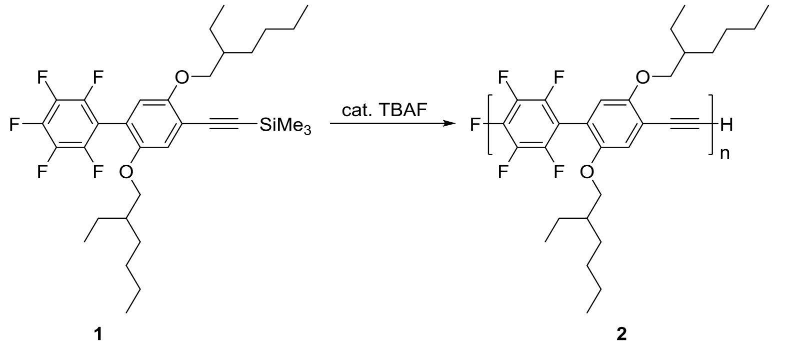

(S3)

A typical polymerization is as follows: A typical polymerization is as follows: In an argon filled glove box, to a THF solution (5 mL) of **1** (164 mg, 275 mmol) was added TBAF (1 M THF solution, 15 μL, 15×10^{-3} mmol, 5 mol %) at room temperature. After stirring for 2 h at room temperature, the mixture was poured into methanol (200 mL). The precipitate was collected by centrifugation. A second cycle of dissolving–precipitation followed by freeze-drying gave polymer **2** as a pale yellow powder (108 mg, 0.214 mmol for the monomer unit, 81%). A yellow powder: M_n = 4400, PDI = 1.31 (SEC relative to polystyrene standards); ^{1}H NMR ($CDCl_3$, 400 MHz) δ 0.89-1.01 (bm, 12H), 1.29-1.83 (m, 18H), 3.86-3.99 (m, 4H), 6.91 (bs, 1H), 7.23 (bs, 1H); ^{13}C{^{1}H} NMR ($CDCl_3$, 100 MHz) δ 11.03. 11.1, 13.9, 14.0, 22.9, 23.0, 29.0, 29.1, 30.5, 30.6, 39.5, 39.7, 71.6, 72.2, 115.6, 116.7, 145. 2 (bm), 147.7 (bm), 150.5, 154.3; ^{19}F{^{1}H} NMR ($CDCl_3$, 376 MHz) δ −140.7-−140.5 (bm, 2F), −137.9-−137.8 (bm, 2F).

Polymerization of 1 with 1-trimethylsilyl-4-[(triethoxysilyl)ethynyl]benzene 3/*t*-BuOK/cryptand[2.2.2]

Synthesis of 1-trimethylsilyl-4-[(triethoxysilyl)ethynyl]benzene 3

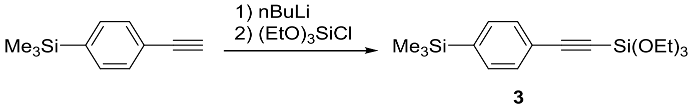

(S4)

To a THF solution (33 mL) of 1-trimethylsilyl-4-(ethynyl)benzene (2.95 g, 16.9 mmol) was added to a 1.6 M hexane solution of n-butyllithium (8.8 mL, 14.1 mmol) at −78 °C and then stirred at 0 °C for 4 h. The mixture was added to a THF solution (42 mL) of triethoxychlorosilane (4.2 g, 21.1 mL) at −78 °C and then stirred at room temperature overnight. The solvent was removed under reduced presser and hexane was added to the residue. After filtration and evaporation of the solvent, the distillation gave the title compound in 39% (2.17 g, 6.40 mmol). A colorless oil: bp 70-105 °C/1 mmHg; ^{1}H NMR ($CDCl_3$, 400 MHz) δ 0.27 (s, 9H), 1.30 (t, J = 7.0 Hz, 9H), 3.95 (q, J = 7.0 Hz, 6H), 7.49 (d, J = 1.6 Hz, 4H); ^{13}C{^{1}H} NMR ($CDCl_3$, 100 MHz) δ −1.3, 18.0, 59.1,85.5, 104.3,

122.2, 131.4, 133.1, 142.5; $^{29}Si\{^1H\}$ NMR ($CDCl_3$, 79.5 MHz) δ −72.4, −3.3; HRMS (FAB, matrix = 3-nitrobenzyl alcohol) calcd for $C_{17}H_{28}O_3Si_2$ ($[M]^+$) *m/z* 336.1577, found 336.1643.

Polymerization of 1 with 1-trimethylsilyl-4-(triethoxysilylethynyl)benzene 3/*t*-BuOK/cryptand[2.2.2]

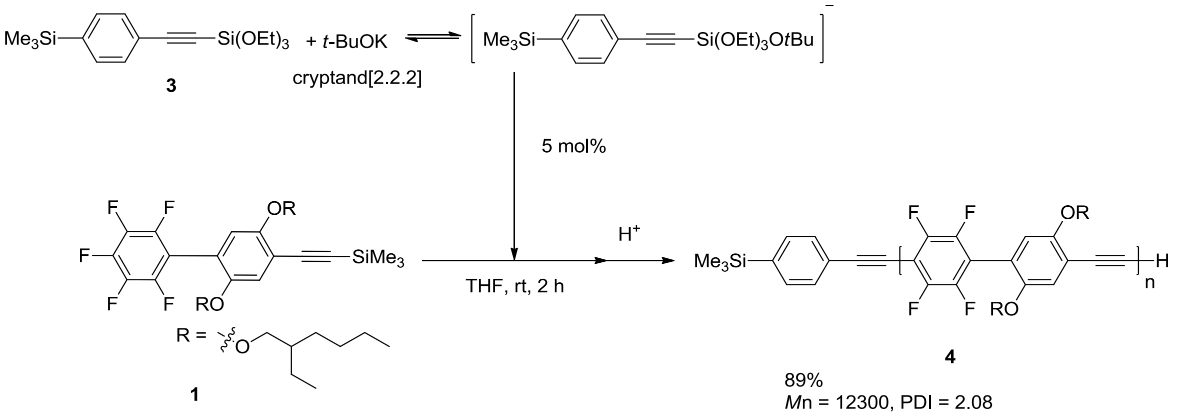

(S5)

A mixture of 1-trimethylsilyl-4-[(triethoxysilyl)ethynyl]benzene **3**, potassium *t*-buthoxide (5.6 mg, 0.017 mmol), and cryptand[2.2.2] (12.7 mg, 0.034 mmol) in THF (2 mL) was stirred at room temperature for 30 min. To the mixture was added a THF solution (2 mL) of **1** (200 mg, 0.335 mmol) at room temperature slowly (0.1 mL min^{-1}). After stirring for 2 h at the temperature, an aliquot of 0.2 mL was quenched with ethanol to analysis of the SEC. The remaining mixture was poured into methanol (200 mL) and the precipitate was collected by centrifugation. A second cycle of dissolving–precipitation followed by freeze-drying gave polymer **4** as a white powder (179 mg, 0.30 mmol for the monomer unit, 89%). A yellow powder: M_n = 12300, PDI = 2.08 (SEC relative to polystyrene standards); ^1H NMR ($CDCl_3$, 400 MHz) δ 0.30 (s), 0.82-0.98 (bm), 1.26-1.83 (bm), 3.32 (s), 3.88 (bs), 6.84 (bs), 7.15 (bs); $^{19}F\{^1H\}$ NMR ($CDCl_3$, 376 MHz) δ −163, −156, −141, −140, −138.

A control initiation experiment using 1-trimethylsilyl-4-(trimethylsilylethynyl)benzene/TBAF for the polymerization of 1

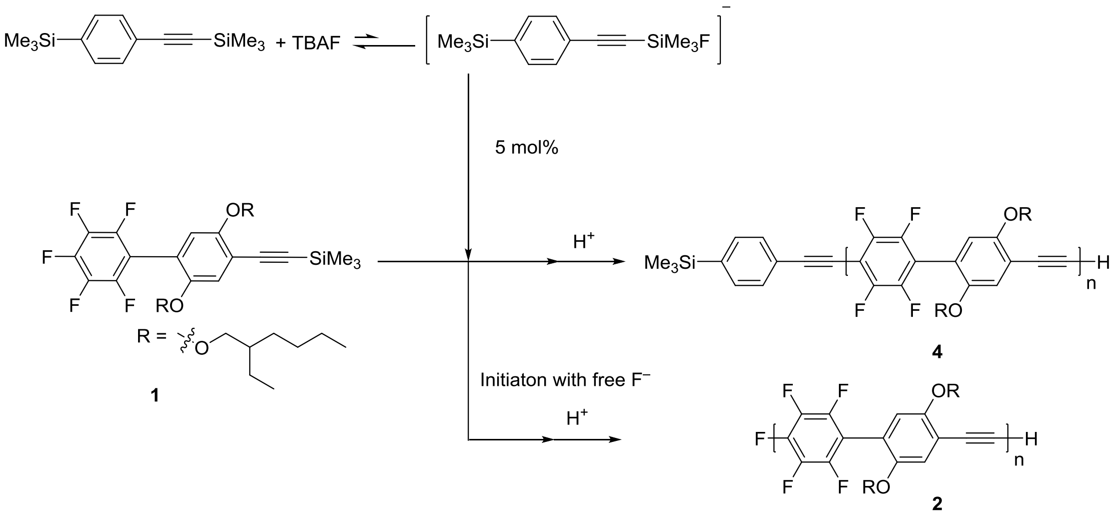

(S6)

To a THF solution (1 mL) of 1-trimethylsilyl-4-[(trimetylsilyl)ethynyl]benzene (40 mg, 1.62×10^{-4} mmol) was added TBAF (1 M THF solution, 15 μL, 15×10^{-3} mmol,) at room temperature. After stirring for 30 min. A 0.1 mL of the mixture was added to **1** (208 mg, 0.349 mmol) in THF (5 mL) at room temperature. After stirring for 2 h at the temperature, the mixture was poured into methanol (200 mL) and the precipitate was collected by centrifugation. A second cycle of dissolving–precipitation followed by freeze-drying gave the polymers with a mixture as a white powder (131 mg, 0.26 mmol for the monomer unit, 74%). A yellow powder: M_n = 3200, PDI = 1.79 (SEC relative to polystyrene standards). The ^{1}H NMR showed the obtained polymer is a mixture of initiation end-capped polymer **4** and no-end-capped polymer **2**. The introduction ratio of the initiator unit was ≈ 10% (Figure S5).

Synthesis of 1,4-bis(3’-trimethylsilylpropoxy)-2-pentafluorophenyl-5-(trimethylsilylethynyl)benzene 5

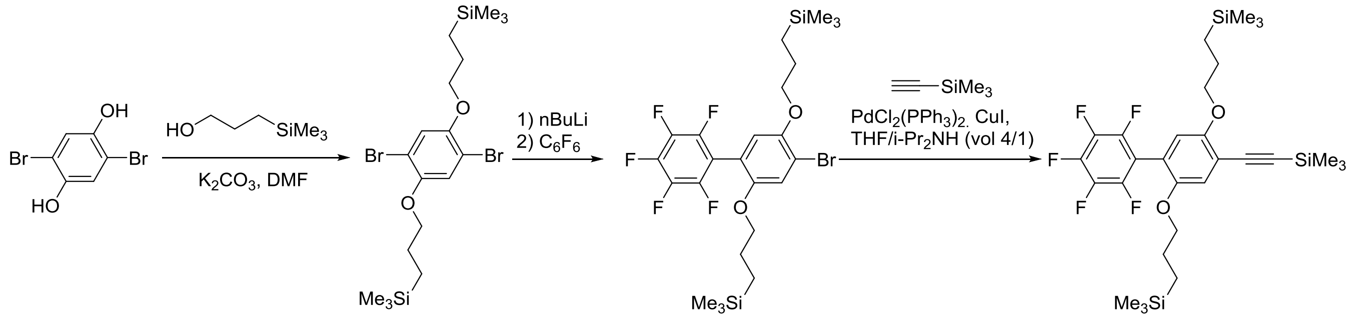

5

(S7)

Synthesis of 2,5-dibromo-1,4-bis(3’-trimethylsilylpropoxy)benzene

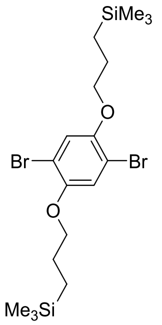

A mixture of 2,5-dibromohydroquinone (4.90 g, 18.0 mmol), 3-bromopropyltrimethylsilane[S3] (7.10 g, 36.3 mmol), and K_2CO_3 (6.56 g, 47.5 mmol) in DMF (60 mL) was stirred at 80 °C for 10 h. After addition of a saturated aqueous solution of NH_4Cl to the mixture, the resulting mixture was extracted with hexane. The organic layer was washed with a saturated aqueous solution of NaCl and dried over anhydrous $MgSO_4$. After filtration and removal of the solvent, the residue was purified with chromatography (SiO_2, hexane) to give the title compound (8.43 g, 17.0 mmol, 94%). A colorless oil; ^{1}H NMR ($CDCl_3$, 400 MHz) δ 0.05 (s, 18H), 0.63-0.66 (m, 4H), 1.81-1.84 (m, 4H),

---

S3 L. H. Sommer, R. E. V. Strien and F. C. Whitmore, *J. Am. Chem. Soc.*, 1949, **71**, 3056.

3.93 (t, J = 6.8 Hz, 4H), 5.10 (s, 2H); $^{13}C\{^1H\}$ NMR ($CDCl_3$, 100 MHz) δ −1.7, 12.5, 23.8, 72.9, 111.4, 118.5, 150.1; $^{29}Si\{^1H\}$ NMR ($CDCl_3$, 79.5 MHz) δ 1.9; HRMS (FAB, matrix = 3-nitrobenzyl alcohol, NBA) calcd for $C_{18}H_{32}Br_2O_2Si_2$ ($[M]^+$) *m/z* 496.4299, found 494.0301.

Synthesis of 2-bromo-1,4-bis(3'-trimethylsilylpropoxy)-5-pentafluorophenylbenzene

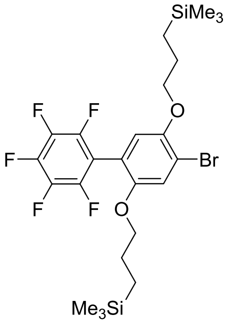

To an ether solution (200 mL) of 2,5-dibromo-1,4-bis(3'-trimethylsilylpropoxy)benzene (8.25 g, 116.6 mmol) was added a hexane solution of n-butyllithium (1.58 M, 12.0 mL, 19.2 mmol) at −40 °C. After stirring the mixture at the temperature for 70 min, hexafluorobenzene (7.0 g, 37.6 mmol) was added to the mixture at one portion, and then the mixture stirred at room temperature overnight. To the mixture was added a saturated aqueous solution of $NaHCO_3$ and the resulting mixture was extracted with ether. The organic layer was washed with a saturated aqueous solution of NaCl and dried over anhydrous $MgSO_4$. After filtration and removal of the solvent, the residue was purified with chromatography (SiO_2, hexane) to give the title compound (8.14 g, 13.9 mmol, 84%). A colorless oil; ^1H NMR ($CDCl_3$, 400 MHz) δ 0.00(5) (s, 9H), 0.06 (2, 9H), 0.47-0.51 (m, 2H), 0.64-0.68 (m, 2H), 1.64-1.70 (m, 2H), 1.83-1.88 (m, 2H), 3.89-3.98 (m, 2H), 6.81 (s, 1H), 6.93 (s, 1H); $^{13}C\{^1H\}$ NMR ($CDCl_3$, 100 MHz) δ −1.9, −1.8, 12.3, 12.5, 23.7, 23.8, 72.0, 73.0, 114.5, 115.0, 116.0, 116.9, 117.6, 118.0, 125.3, 128.2, 129.0, 136.3, 136.36, 137.9, 137.8, 138.9, 138.8, 139.0, 143.2, 145.6, 145.7, 145.7, 149.6, 150.4, 151.0; $^{19}F\{^1H\}$ NMR ($CDCl_3$, 376 MHz) δ −163.5-−163.1 (m, 2F), −155.9-−155.6 (m, 1F), −139.9-−139.6 (m, 2F); $^{29}Si\{^1H\}$ NMR ($CDCl_3$, 79.5 MHz) δ 1.8, 1.9; HRMS (FAB, matrix = 3-nitrobenzyl alcohol, NBA) calcd for $C_{24}H_{32}BrF_5O_2Si_2$ ($[M]^+$) *m/z* 583.5839, found 582.1049.

1,4-bis(3'-trimethylsilylpropoxy)-2-pentafluorophenyl-5-(trimethylsilylethynyl)benzene 5

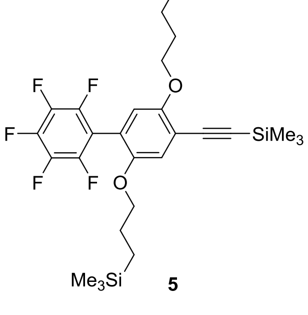

5

To 2-bromo-1,4-bis(3'-trimethylsilylpropoxy)-5-pentafluorophenylbenzene (7.17 g, 12.3 mmol), $PdCl_2(PPh_3)_2$ (98 mg, 0.14 mmol), and CuI (96 mg, 0.50 mmol) in THF/*i*-Pr_2NH (100 mL, v/v = 4/1) was added trimethylsilylacetylene (2.40 g, 24.4 mmol) at room temperature. The mixture was stirred at the temperature for 10 min and then at 50 °C overnight. To the mixture was added a

saturated aqueous solution of NH_4Cl and the resulting mixture was extracted with hexane. The organic layer was washed with a saturated aqueous solution of NaCl and dried over anhydrous $MgSO_4$. After filtration and removal of the solvent, the residue was purified with chromatography (SiO_2, hexane/toluene = 10/1) to give **5** (2.70 g, 3.44 mmol, 30%). A colorless oil; ^{1}H NMR ($CDCl_3$, 400 MHz) δ 0.00(9) (s, 9H), 0.05 (s, 9H), 0.31 (s, 9H), 0.46-0.50 (m, 2H), 0.67-0.72 (m, 2H), 1.62-1.66 (m, 2H), 1.82-1.86 (m, 2H), 3.89-3.98 (m, 4H), 6.75 (s, 1H), 7.08 (s, 1H); ^{13}C{^{1}H} NMR ($CDCl_3$, 100 MHz) δ −1.93, −1.77, −0.02, 12.3, 12.7, 23.7, 24.0, 71.8, 72.4, 76.7, 77.0, 77.3, 99.8, 100.8, 112.5, 115.1, 116.4, 116.6, 117.3, 143.1, 145.5, 150.3, 154.15; ^{19}F{^{1}H} NMR ($CDCl_3$, 376 MHz) δ −163.4 (dt, J = 21, 8.2 Hz, 2F), −156.0 (t, J = 21 Hz, 1F), −139.8 (dd, J = 21, 8.2 Hz, 2F); ^{29}Si{^{1}H} NMR ($CDCl_3$, 79.5 MHz) δ −17.6, 1.74, 1.86; HRMS (FAB, matrix = 3-nitrobenzyl alcohol, NBA) calcd for $C_{29}H_{41}F_5O_2Si_3$ ([M]$^+$) m/z 600.2335, found 600.2335.

Polymerization of 1,4-bis(3’-trimethylsilylpropoxy)-2-pentafluorophenyl-5-(trimethylsilylethynyl)benzene 5

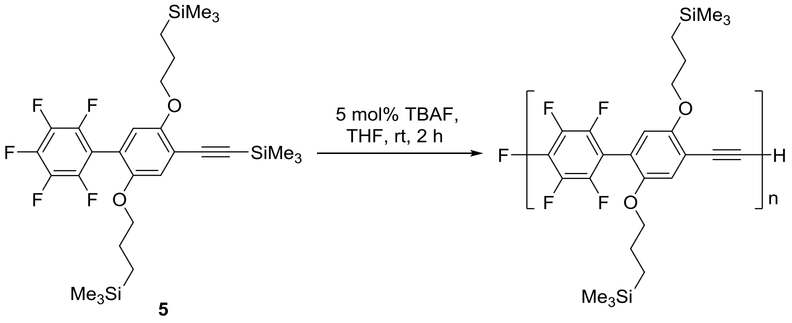

(S8)

In an argon filled glove box, to a THF solution (2 mL) of 1,4-bis(3’-trimethylsilylpropoxy)-2-pentafluorophenyl-5-(trimethylsilylethynyl)benzene **5** (180 mg, 300 mmol) was added TBAF (1 M THF solution, 15 μL, 15×10^{-3} mmol, 5 mol %) at room temperature. After stirring for 2 h at the temperature, the mixture was poured into methanol (200 mL). The precipitate was collected by centrifugation. A second cycle of dissolving–precipitation followed by freeze-drying gave the polymer as a pale yellow powder (92.3 mg, 0.181 mmol for the monomer unit, 60%). A yellow powder: M_n = 4000, PDI = 1.42 (SEC relative to polystyrene standards); ^{1}H NMR ($CDCl_3$, 400 MHz) δ 0.00-0.05 (bm), 0.50, 0.72, 1.57, 1.69, 1.88, 3.38, 3.92-34.04, 6.82-6.83, 6.88, 7.12, 7.19-7.20; ^{13}C{^{1}H} NMR ($CDCl_3$, 100 MHz) δ 1.8, 12.3, 23.7, 71.9, 72.5, 113.6, 116.21, 117.0, 117.6, 143.1, 144.9, 145.6, 147.7, 150.4, 154.1; ^{19}F{^{1}H} NMR ($CDCl_3$, 376 MHz) δ −163.2, −155.7, −140.7-−140.5 (bm), −139.7, −137.8,-−137.7 (bm).

Synthesis of block copolymer 6

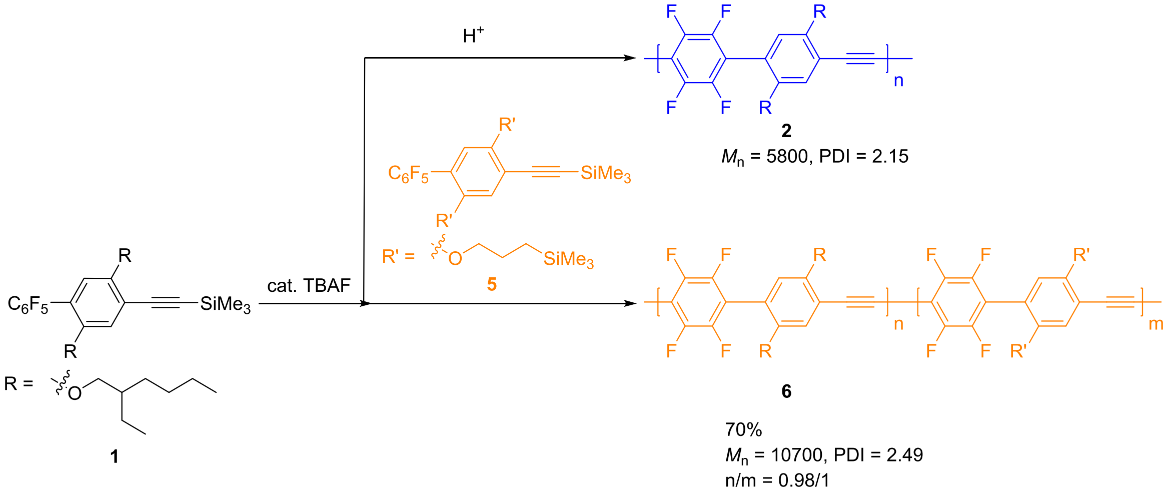

(S9)

In an argon filled glove box, to a THF solution (2 mL) of **1** (180 mg, 0.302 mmol) was added TBAF (1 M THF solution, 15 μL, 1.5×10^{-2} mmol, 5 mol %) at room temperature. After stirring for 2 h at the temperature, an aliquot of 0.2 mL was quenched with ethanol and subjected to SEC analysis (**2**: M_n = 5800, PDI = 2.15).

For the second polymerization, to the remaining reaction mixture was added a 1 mL THF solution of 1,4-bis(3'-trimethylsilylpropoxy)-2-pentafluorophenyl-5-(trimethylsilylethynyl)benzene **5** (179 mg, 0.30 mmol) at room temperature. After stirred at room temperature for additional 2 h, 0.2 mL of ethanol was added. After purification in a similar manner as mentioned above, block copolymer **6** was obtained (214 mg, 70%). A pale yellow powder: M_n = 10700, PDI = 2.49 (SEC relative to polystyrene standards), n/m = 0.98, ^{1}H NMR ($CDCl_3$, 400 MHz) δ 0.00-0.05 (bm), 0.50, 0.82, 0.89-1.00 (m), 1.28-1.68 (bm), 3.91-4.04 (bm), 6.89, 7.22; ^{13}C{^{1}H} NMR ($CDCl_3$, 100 MHz) δ −1.9, −1.8, 11.1, 11.2, 12.3, 12.5, 14.0, 14.1, 23.0, 23.1, 23.7, 23.8, 23.9, 23.9, 29.0, 29.1, 30.4, 30.5, 39.4, 39.6, 71.4, 71.9, 72.0, 72.5, 113.4, 115.4, 116.1, 116.6, 117.0, 143.0, 144.9, 145.6, 147.5, 150.3, 150.4, 154.1, 154.2; ^{19}F{^{1}H} NMR ($CDCl_3$, 376 MHz) δ −163.2, −155.6, −140.7-−140.5 (bm), −139.6, −138.1-−137.7 (bm).

Synthesis of 2-pentafluoropheny-3,4-(2′,2′-dibutylpropylenedioxy)-5-(trimethylsilylethynyl)thiophene 7

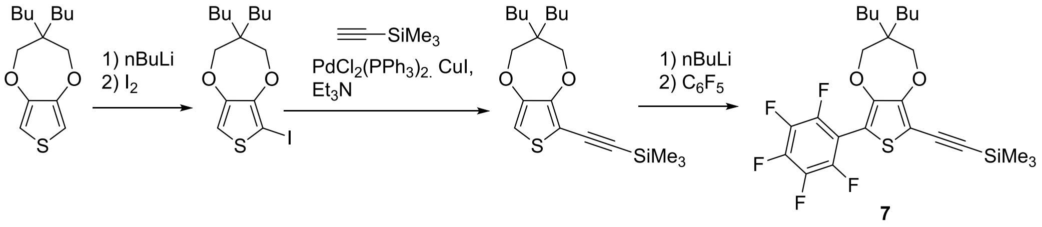

(S10)

2-Iodo-3,4-(2′,2′-dibutylpropylenedioxy)thiophene

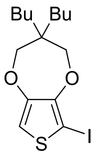

To a THF solution (100 mL) of 3,4-(2′,2′-dibutylpropylenedioxy)thiophene (3.93g, 14.6 mmol) was added a hexane solution of 1.58 M n-butyllithium (10.2 mL, 16.1 mmol) at −78 °C for 10 min. The mixture was stirred at the temperature for 30 min and then iodide (6.31 g, 24.9 mmol) was added to the mixture at 0 °C. After the mixture was stirred at room temperature overnight, 10% aqueous sodium thiosulfate solution was added to the mixture until disappearance of the reddish color. The resulting mixture was extracted with hexane. The organic layer was washed with a saturated aqueous solution of NaCl and dried over anhydrous $MgSO_4$. After filtration and removal of the solvent, the residue was purified with chromatography (SiO_2, hexane/toluene = 10/1) to give the title compound (2.70 g, 6.84 mmol, 47%). A pale yellow oil; ^{1}H NMR ($CDCl_3$, 400 MHz) δ 0.96 (t, *J* = 7.2 Hz, 6H), 1.25-1.41 (m, 14H), 3.87 (s, 2H), 3.94 (s, 2H), 6.67 (s, 1H); ^{13}C{^{1}H} NMR ($CDCl_3$, 100 MHz) δ 14.0, 23.5, 25.0, 31.6, 43.7, 56.1, 77.8, 77.9, 104.6, 109.6, 148.5, 151.90; HRMS calcd for $C_{15}H_{23}O_2S_1$ ($[M]^+$) *m/z* 394.3169, found 394.0472.

2-Trimethylsilylethynyl-3,4-(2′,2′-dibutylpropylenedioxy)thiophene

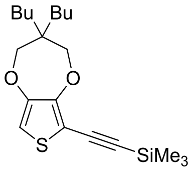

To 2-iodo-3,4-(2′,2′-dibutylpropylenedioxy)thiophene (2.2 g, 5.6 mmol), $PdCl_2(PPh_3)_2$ (198 mg, 0.28 mmol), and CuI (122 mg, 0.64 mmol) in Et_3N (10 mL) was added trimethylsilylacetylene (2.1 g, 21.4 mmol) at room temperature. The mixture was stirred at 40 °C overnight. To the mixture was added a saturated aqueous solution of NH_4Cl and the resulting mixture was extracted with hexane. The organic layer was washed with a saturated aqueous solution of NaCl and dried over anhydrous $MgSO_4$. After filtration and removal of the solvent, the residue was purified with chromatography (SiO_2, hexane/toluene = 5/1) to give the title compound (1.85 g, 5.1 mmol, 91%). A pale yellow oil; ^{1}H NMR ($CDCl_3$, 400 MHz) δ 0.28 (s, 9H), 0.97 (t, *J* = 7.2 Hz, 6H), 1.28-1.45 (m, 12H), 3.87 (s, 2H), 3.99 (s, 2H), 6.37 (s, 1H); ^{13}C{^{1}H} NMR ($CDCl_3$, 100 MHz) δ 0.02, 14.0, 23.5, 25.0, 31.6, 43.7, 77.6, 77.8, 105.7, 125.3, 128.2, 129.0, 148.7, 152.5; ^{29}Si{^{1}H} NMR ($CDCl_3$, 79.5 MHz) δ −17.5; HRMS calcd for $C_{20}H_{32}O_2SSi$ ($[M]^+$) *m/z* 364.6244, found 364.1901.

2-pentafluoropheny-3,4-(2′,2′-dibutylpropylenedioxy)-5-(trimethylsilylethynyl)thiophene 7

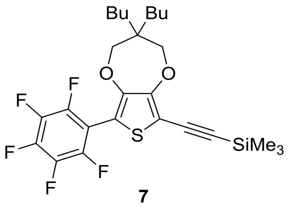

7

To a THF solution (50 mL) of 2-trimethylsilylethynyl-3,4-(2′,2′-dibutylpropylenedioxy)thiophene (1.65 g, 4.52 mmol) was added a hexane solution of n-butyllithium (1.58 M, 3.50 mL, 5.53 mmol) at −78 °C for 5 min. The mixture was stirred at the same temperature for 30 min and then to 0 °C for 30 min. To the mixture was added hexafluorobenzene at one portion at −40 °C and then the mixture was stirred at room temperature overnight. To the mixture was added a saturated aqueous

solution of $NaHCO_3$ and the resulting mixture was extracted with hexane. The organic layer was washed with a saturated aqueous solution of NaCl and dried over anhydrous $MgSO_4$. After filtration and removal of the solvent, the residue was purified with chromatography (SiO_2, hexane/toluene = 20/1) to give **7** (0.48 g, 0.905 mmol, 22%). A pale yellow oil; ^{1}H NMR ($CDCl_3$, 400 MHz) δ 0.34 (s, 9H), 0.99 (t, J = 7.2 Hz, 6H), 1.37-1.46 (m, 12H), 3.77 (s, 2H), 4.07 (s, 2H); ^{13}C{^{1}H} NMR ($CDCl_3$, 100 MHz) δ −0.10, 14.0, 21.4, 23.5, 25.0, 31.6, 43.9, 77.9, 78.1, 94.5, 103.7, 104.7, 105.8, 107.3, 125.3, 128.2, 129.0, 136.6, 137.9, 139.1, 143.6, 146.1, 147.8, 152.1; ^{19}F{^{1}H} NMR ($CDCl_3$, 376 MHz) δ −162.3 (td, J = 21, 6.8 Hz, 2F), −154.3 (t, J = 21 Hz, 1F), −138.4 (dd, J = 21, 6.8 Hz, 2F); ^{29}Si{^{1}H} NMR ($CDCl_3$, 79.5 MHz) δ −17.0; HRMS (FAB, matrix = 3-nitrobenzyl alcohol, NBA) calcd for $C_{26}H_{31}F_5O_2SSi$ ($[M]^+$) m/z 530.6745, found 530.1733.

Polymerization of 2-pentafluoropheny-3,4-(2′,2′-dibutylpropylenedioxy)-5-(trimethylsilylethynyl)thiophene 7

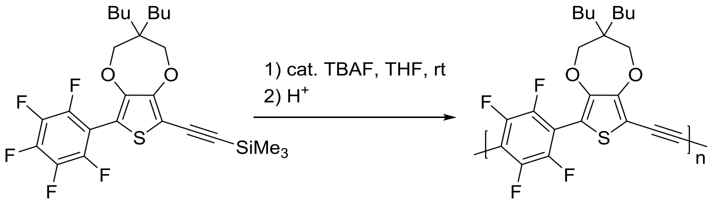

7 10

(S11)

In an argon filled glove box, to a THF solution (5 mL) of **7** (315 mg, 0.593 mmol) was TBAF (1 M THF solution, 5.0 μL, 5.0 × 10^{-3} mmol) at room temperature. After stirring for 2 h at the temperature, 2 mL EtOH was added to the mixture. The mixture was poured into methanol (200 mL) and the precipitate was collected by centrifugation. A second cycle of dissolving–precipitation followed by freeze-drying gave polymer **10** as a white powder (71.6 mg, 0.163 mmol for the monomer unit, 52%). A yellow powder: M_n = 11200, PDI = 2.09 (SEC relative to polystyrene standards); ^{1}H NMR ($CDCl_3$, 400 MHz) δ 0.90-0.96 (bm, 6H), 1.30-1.58 (bm, 12H), 3.98-4.10 (bs, 4H); ^{13}C{^{1}H} NMR ($CDCl_3$, 100 MHz) δ 14.0, 23.5, 25.0, 31.6, 31.6, 43.9, 78.0 (bm), 145.4 (bm), 147.9 (bm), 148.1 (bm); ^{19}F{^{1}H} NMR ($CDCl_3$, 376 MHz) δ −139.3-−139.1 (bm), −138.2-−138.1 (bm).

Synthesis of 2-pentafluorophenyl-7-trimethylsilylethynyl-9,9-di(*n*-octyl)fluorene 8

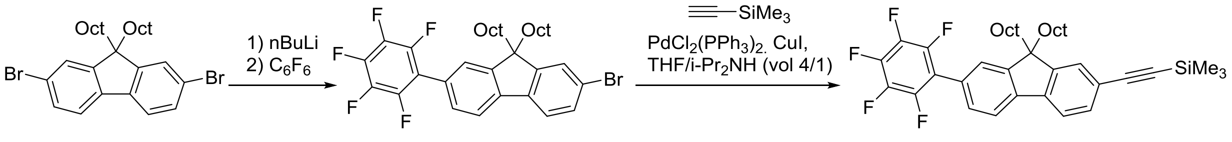

8

(S12)

2-bromo-7-pentafluorophenyl-9,9-di(*n*-octyl)fluorene

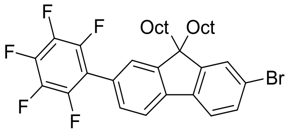

To 2,7-dibromo-9,9-di(*n*-octyl)fluorene (5.0 g, 9.17 mmol) in a mixture of THF (100 mL) and ether (100 mL) was added a hexane solution (1.58 M) of n-butyllithium (6.06 mL, 9.57 mmol) at −78 °C. After stirring at the temperature for 1 h, hexafluorobenzene (1.15 g, 10.0 mmol) was added at the same temperature and the mixture was stirred at room temperature overnight. After addition of methanol, the organic layer was extracted with hexane and washed with a saturated aqueous solution of NaCl and dried over anhydrous $MgSO_4$. After filtration and removal of the solvent, the residue was purified with chromatography (SiO_2, hexane) to give the title compound (1.09 g, 1.71 mmol, 20%). A white waxy oil; ^{1}H NMR ($CDCl_3$, 400 MHz) δ 0.64 (bs, 4H), 0.82 (t, *J* = 7.2 Hz, 6H), 1.05-1.23 (m, 20H), 1.92-1.99 (m, 4H), 7.35-7.40 (m, 2H), 7.47-7.49 (m, 2H), 7.56 (d, *J* = 9.0 Hz, 1H), 7.77 (d, *J* = 7.8 Hz, 1H); ^{13}C{^{1}H} NMR ($CDCl_3$, 100 MHz) δ 0.0, 14.0, 22.6, 23.7, 23.7, 29.1, 29.8, 29.9, 31.7, 40.1, 40.2, 55.6, 76.7, 77.0, 77.3, 120.0, 121.4, 121.9, 123.0, 124.9, 125.1, 126.3, 129.1, 130.2, 139.2, 141.2 150.7, 153.4; ^{19}F{^{1}H} NMR ($CDCl_3$, 376 MHz) δ −162.3 (td, *J* = 21, 7.5 Hz, 2F), −155.9 (t, *J* = 21 Hz, 1F), −143.0 (dd, *J* = 21, 7.5 Hz, 2F); HRMS (FAB, matrix = 3-nitrobenzyl alcohol, NBA) calcd for $C_{35}H_{40}BrF_5$ ($[M]^+$) *m*/*z* 634.2234, found 634.2219.

2-pentafluorophenyl-9,9-di(*n*-octyl)-7-(trimethylsilylethynyl)fluorene 8

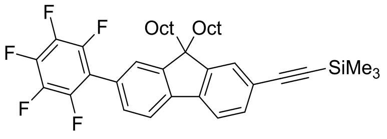

8

To 2-bromo-7-pentafluorophenyl-9,9-di(*n*-octyl)fluorene (1.09 g, 1.71 mmol), $PdCl_2(PPh_3)_2$ (72 mg, 0.10 mmol), and CuI (32 mg, 0.17 mmol) in THF/i-Pr_2NH (20 mL, v/v = 10/1) was added trimethylsilylacetylene (0.26 mL, 1.90 mmol). The mixture was stirred at 70 °C for overnight. To the mixture was added a saturated aqueous solution of NH_4Cl and the resulting mixture was extracted with ethyl acetate. The organic layer was washed with a saturated aqueous solution of NaCl and dried over anhydrous $MgSO_4$. After filtration and removal of the solvent, the residue was purified with chromatography (SiO_2, hexane) to give **8** (720 mg, 1.10 mmol, 64%). A colorless oil; ^{1}H NMR ($CDCl_3$, 400 MHz) δ 0.30 (s, 9H), 0.62 (bs, 4H), 0.82 (t, *J* = 5.5 Hz, 6H), 1.04-1.26 (m, 22H), 1.97 (t, *J* = 6.3 Hz, 4H), 7.38-7.41 (m, 2H), 7.47-7.50 (m, 2H), 7.67 (d, *J* = 5.8 Hz, 1H), 7.78 (d, *J* = 5.8 Hz, 1H); ^{13}C{^{1}H} NMR ($CDCl_3$, 100 MHz) δ 0.0, 14.0, 22.6, 23.7, 29.1, 29.9, 31.6, 31.7, 40.2, 55.4, 76.7, 94.4, 106.0, 119.9, 120.2, 122.1, 124.9, 125.1, 126.4, 129.0, 131.3, 140.6, 141.5, 151.1, 151.4; ^{19}F{^{1}H} NMR ($CDCl_3$, 376 MHz) δ −162.6 (td, *J* = 21, 8.2 Hz, 2F), −156.2 (t, *J* = 21 Hz, 1F), −143.2 (dd, *J* = 21, 8.2 Hz, 2F); HRMS (FAB, matrix = 3-nitrobenzyl alcohol, NBA) calcd for $C_{40}H_{49}F_5O_2Si$ ($[M]^+$) *m*/*z* 652.3524, found 652.3514.

Polymerization of 2-pentafluorophenyl-9,9-di(*n*-octyl)-7-(trimethylsilylethynyl)fluorene 8

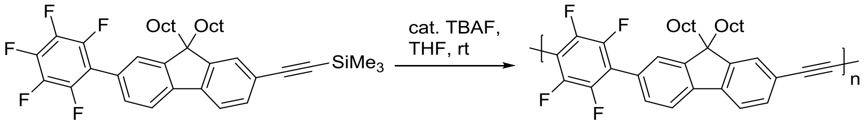

8 **11** (S13)

In an argon filled glove box, to a THF solution (6 mL) of **8** (508 mg, 0.389 mmol) was TBAF (1 M THF solution, 5.0 μL, 5.0 × 10^{-3} mmol) at room temperature. After stirring for 2 h at the temperature, 2 mL EtOH was added to the mixture. The mixture was poured into methanol (200 mL) and the precipitate was collected by centrifugation. A second cycle of dissolving–precipitation followed by freeze-drying gave polymer **11** as a white powder (108 mg, 0.183 mmol for the monomer unit, 86%). A yellow powder: M_n = 10600, PDI = 1.99 (SEC relative to polystyrene standards); ^{1}H NMR ($CDCl_3$, 400 MHz) δ 0.70-0.92 (bm), 1.13-1.27 (bm), 2.07 (bs), 3.20 (s), 7.55-7.91 (bm); ^{13}C{^{1}H} NMR ($CDCl_3$, 100 MHz) δ 14.0, 22.6, 23.8, 29.2, 29.9, 23.5, 40.1, 55.6, 55.6, 120.3, 120.4, 120.8, 125.0, 126.3, 129.1, 129.2, 131.4, 141.3, 141.8, 151.5, 151.5; ^{19}F{^{1}H} NMR ($CDCl_3$, 376 MHz) δ −162.5, −155.97, −144.1—144.0 (bm), −143.2, −137.6-−137.5 (bm).

Synthesis of 2,3,5,6-tetra(2’-ethylhexyloxy)-1-trimethylsilyethynyl-4-[(pentafluorophenyl)ethynyl]benzene 9

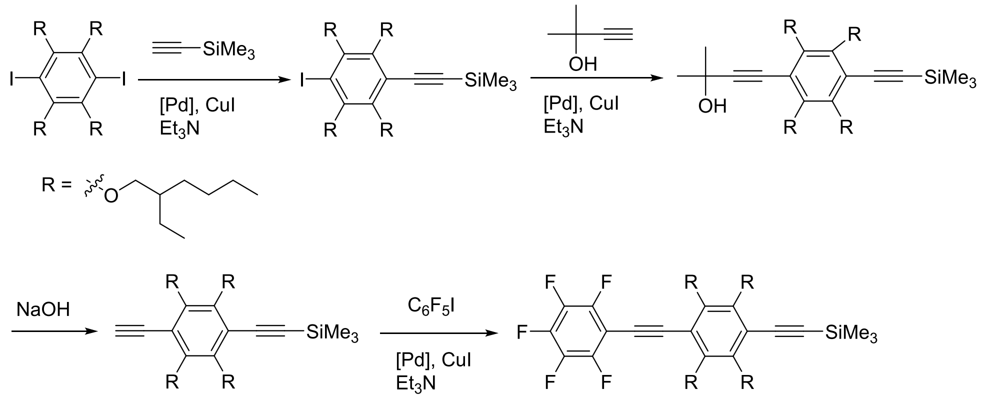

(S14)

1-trimethylsilylethynyl-2,3,5,6,-tetra(2’-ethylhexyloxy)-4-iodobenzene

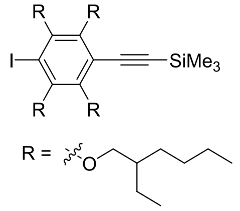

To 1,4-diiodo-2,3,5,6,-tetra(2’-ethylhexyloxy)benzene[S4] (6.70 g, 7.95 mmol), $PdCl_2(PPh_3)_2$ (280 mg, 0.40 mmol), and CuI (150 mg, 0.80 mmol) in Et_3N (50 mL) was added trimethylsilylacetylene (0.67 mL, 4.77 mmol). The mixture was stirred at room temperature for overnight. To the mixture was added a saturated aqueous solution of NH_4Cl and the resulting mixture was extracted with ethyl acetate. The organic layer was washed with a saturated aqueous solution of NaCl and dried over anhydrous $MgSO_4$. After filtration and removal of the solvent, the residue was purified with chromatography (SiO_2, hexane/toluene = 20/1 -10/1) to give the title compound (1.10 g, 1.35 mmol, 17%). A pale yellow oil; ^{1}H NMR ($CDCl_3$, 400 MHz) δ 0.26 (s, 9H), 0.89-0.96 (m, 24H), 1.33-1.82

S4 T. Dutta, K. B. Woody and M. D. Watson, *J. Am. Chem. Soc.* 2008, **130**, 452.

(m, 36H), 3.80 (d, *J* = 6.4 Hz, 4H), 3.87-3.95 (m, 4H); $^{13}C\{^1H\}$ NMR ($CDCl_3$, 100 MHz) δ −0.1, 11.2, 14.1, 23.1, 23.7, 29.32, 30.3, 30.3, 40.4, 40.5, 77.5, 93.9, 97.2, 103.9, 128.2, 129.0, 148.9, 150.32; $^{29}Si\{^1H\}$ NMR ($CDCl_3$, 79.5 MHz) δ −17.9; HRMS (FAB, matrix = 3-nitrobenzyl alcohol) calcd for C43H77O4Si ($[M]^+$) *m/z* 812.4636, found 812.4662.

1-trimethylsilylethynyl-2,3,5,6-tetra(2’-ethylhexyloxy)-4-ethynylbenzene

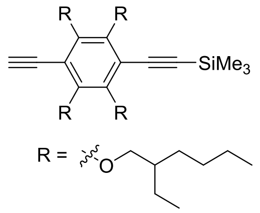

To 1-trimethylsilylethnyl-2,3,5,6,-tetra(2’-ethylhexyloxy)-4-iodobenzene (8.10 g, 9.96 mmol), $PdCl_2(PPh_3)_2$ (700 mg, 1.00 mmol), and CuI (380 mg, 1.99 mmol) in piperidine (100 mL) was added 2-methyl-3-butyne-2-ol (4.87 mL, 49.8 mmol). The mixture was stirred at 50 °C for overnight. After removal piperidine under reduced pressure, to the mixture was added a saturated aqueous solution of NH_4Cl and the resulting mixture was extracted with ethyl acetate. The organic layer was washed with a saturated aqueous solution of NaCl and dried over anhydrous $MgSO_4$. After filtration and removal of the solvent, the residue was purified with chromatography (SiO_2, hexane/dichloromethane = 10/1 -1/1) to give 2-methyl-4-(2,3,5,6-tetra(2’-ethylhexyloxy)-4-((trimethylsilyl)ethynyl)phenyl)but-3-yn-2-ol (6.10 g, 7.93 mmol, 77%). A pale yellow oil; ^1H NMR ($CDCl_3$, 400 MHz) δ 0.07 (s, 9H), 0.87-0.94 (m, 24H), 1.31-1.77 (m, 42H), 2.00 (s, 1H), 3.83-3.96 (m, 8H); $^{13}C\{^1H\}$ NMR ($CDCl_3$, 100 MHz) δ −0.1, 11.2, 14.1, 23.1, 23.7, 29.3, 30.2, 30.4, 31.4, 40.5, 40.6, 65.8, 74.9, 97.1, 102.8, 104.2, 113.97, 114.2, 149.8, 150.3; HRMS (FAB, matrix = 3-nitrobenzyl alcohol) calcd for $C_{48}H_{84}O_5Si$ ($[M]^+$) *m/z* 768.6088, found 768.6082. Without further purification, the next reaction was examined. A toluene solution (100 mL) of 2-methyl-4-(2,3,5,6-tetra(2’-ethylhexyloxy)-4-((trimethylsilyl)ethynyl) phenyl)but-3-yn-2-ol and NaOH (1.6 g, 39.7 mmol) was refluxed for 6 h. After filtration of the mixture and evaporation of solvent, the title compound was obtained (87%, 4.90 g, 6.89 mmol). A pale yellow oil; ^1H NMR ($CDCl_3$, 400 MHz) δ 0.24 (s, 9H), 0.77-0.94 (m, 24H), 1.30-1.73 (m, 36H), 3.86-3.96 (m, 9H); $^{13}C\{^1H\}$ NMR ($CDCl_3$, 100 MHz) δ −0.1, 11.1, 14.1, 23.1, 23.6, 23.7, 29.2, 29.3, 30.3, 40.5, 40.5(1), 76.2, 76.3, 76.7, 86.4, 86.5, 97.1, 104.3, 113.6, 114.0, 114.7, 150.4, 150.5, 150.6; $^{29}Si\{^1H\}$ NMR ($CDCl_3$, 79.5 MHz) δ −17.9; HRMS (FAB, matrix = 3-nitrobenzyl alcohol) calcd for $C_{45}H_{78}O_4Si$ ($[M]^+$) *m/z* 710.5569, found 710.5665.

2,3,5,6-tetra(2’-ethylhexyloxy)-1-trimethylsilyethynyl-4-(pentafluorophenyl)ethynylbenzene 9

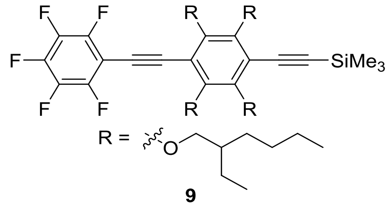

9

A mixture of 1-trimethylsilylethynyl-2,3,5,6,-tetra(2’-ethylhexyloxy)-4-ethynylbenzene (4.90 g,

6.89 mmol), pentafluoroiodobenzene (9.13 mL, 22.1 mmol), $PdCl_2(PPh_3)_2$ (0.48 g, 0.69 mmol), and CuI (0.26 g, 1.38 mmol) in triethylamine (100 mL) was stirred at 40 °C for overnight. After filtration of the mixture and evaporation of the solvent, the residue was purified with chromatography (SiO_2, hexane/dichloromethane = 9/1) and GPC to give **9** in 20% (1.23 g, 1.40 mmol). A waxy solid; ^{1}H NMR ($CDCl_3$, 400 MHz) δ 0.06 (s, 9H), 0.28 (s, 9H), 0.61-0.65 (m, 2H), 1.79-1.83 (m, 2H), 2.39 (s, 1H), 3.93 (t, *J* = 6.8 Hz, 2H), 6.83 (d, *J* = 4.4 Hz, 2H), 7.42 (d, *J* = 4.4 Hz, 2H); ^{13}C{^{1}H} NMR ($CDCl_3$, 100 MHz) δ −1.7, 0.1, 12.6, 23.8, 70.7, 92.3, 105.4, 114.4, 115.0, 133.5, 159.3; ^{29}Si{^{1}H} NMR ($CDCl_3$, 79.5 MHz) δ −18.2, 1.9; HRMS (FAB, matrix = 3-nitrobenzyl alcohol) calcd for $C_{17}H_{28}OSi_2$ ($[M]^+$) *m/z* 304.1679, found 304.1680.

Polymerization of 2,3,5,6-tetra(2’-ethylhexyloxy)-1-trimethylsilyethynyl-4-(pentafluorophenyl)ethynylbenzene 9 with TBAF

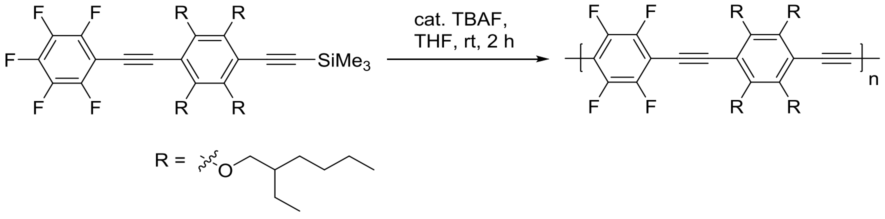

9 12 (S15)

In an argon filled glove box, to a THF solution (2 mL) of **9** (120 mg, 0.14 mmol) was TBAF (1 M THF solution, 7.0 μL, 7.0 × 10^{-3} mmol) at room temperature. After stirring for 2 h at the temperature, 2 mL EtOH was added to the mixture. The mixture was poured into methanol (200 mL) and the precipitate was collected by centrifugation. A second cycle of dissolving–precipitation followed by freeze-drying gave polymer **13** as a pale yellow powder (110 mg, 86%). A yellow powder: M_n = 11300, PDI = 1.85 (SEC relative to polystyrene standards); ^{1}H NMR ($CDCl_3$, 400 MHz) δ 0.84-0.96 (m), 1.27-1.81(m), 3.51 (bs), 3.94 (bs), 7.51 (s), 7.71 (bs); ^{19}F{^{1}H} NMR ($CDCl_3$, 376 MHz) δ −161.7, −152.0, −136.2, −135.1.

Surface-terminated polymerization of 1 with Au nano dot array

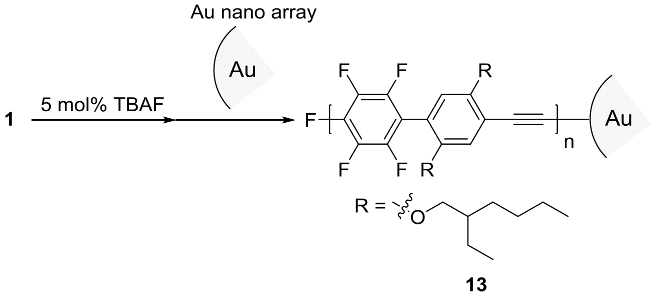

13 (S16)

Au nanoparticle array (ϕ 15 nm) was prepared on a glass substrate according to the reported procedure.[S5] The detail will be reported in a forthcoming paper.

S5 S. Hadano, H. Handa, K. Nagai, T. Iyoda, J. Li and S. Watanabe, *Chem. Lett.*, 2013, **42**, 73.

To a THF solution (1 mL) of **1** (50 mg, 8.4×10^{-2} mmol) was added a 1 M THF solution of TBAF (5 μL, 33 mmol) at room temperature. After stirred for 1 h, an Au nanoparticle array was added to the polymerization mixture. The mixture was strand for 1 h and then the plate was picked up. The substrate was washed with THF and then subjected to the Raman spectroscopy. The SEC analysis of the polymerization mixture shows the resulting polymer has a M_n of 4700 and a PDI of 1.79.

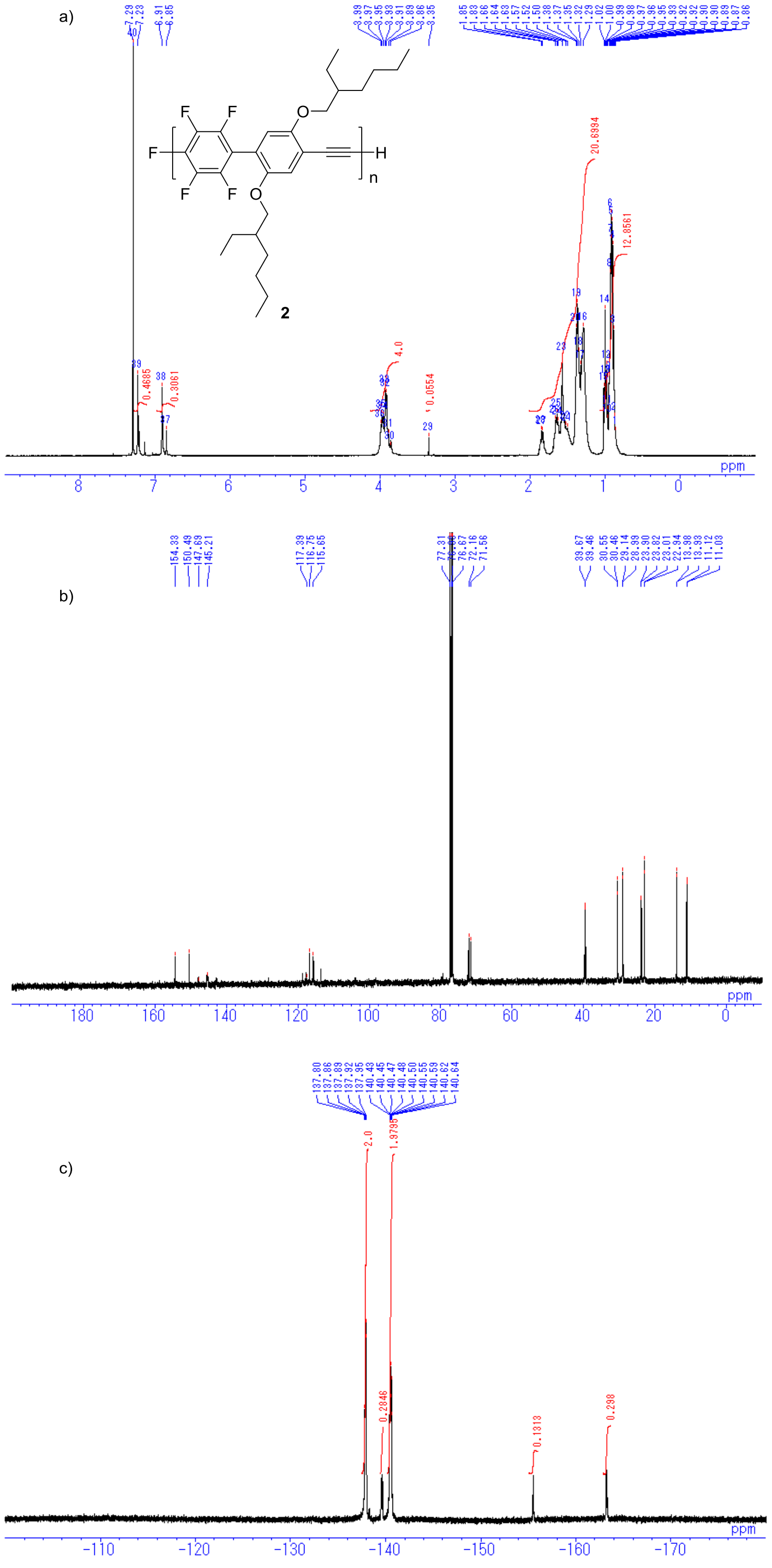

Fig. S1 a) ^{1}H, b) ^{13}C, and c) ^{19}F NMR spectra of **2** ($CDCl_3$).

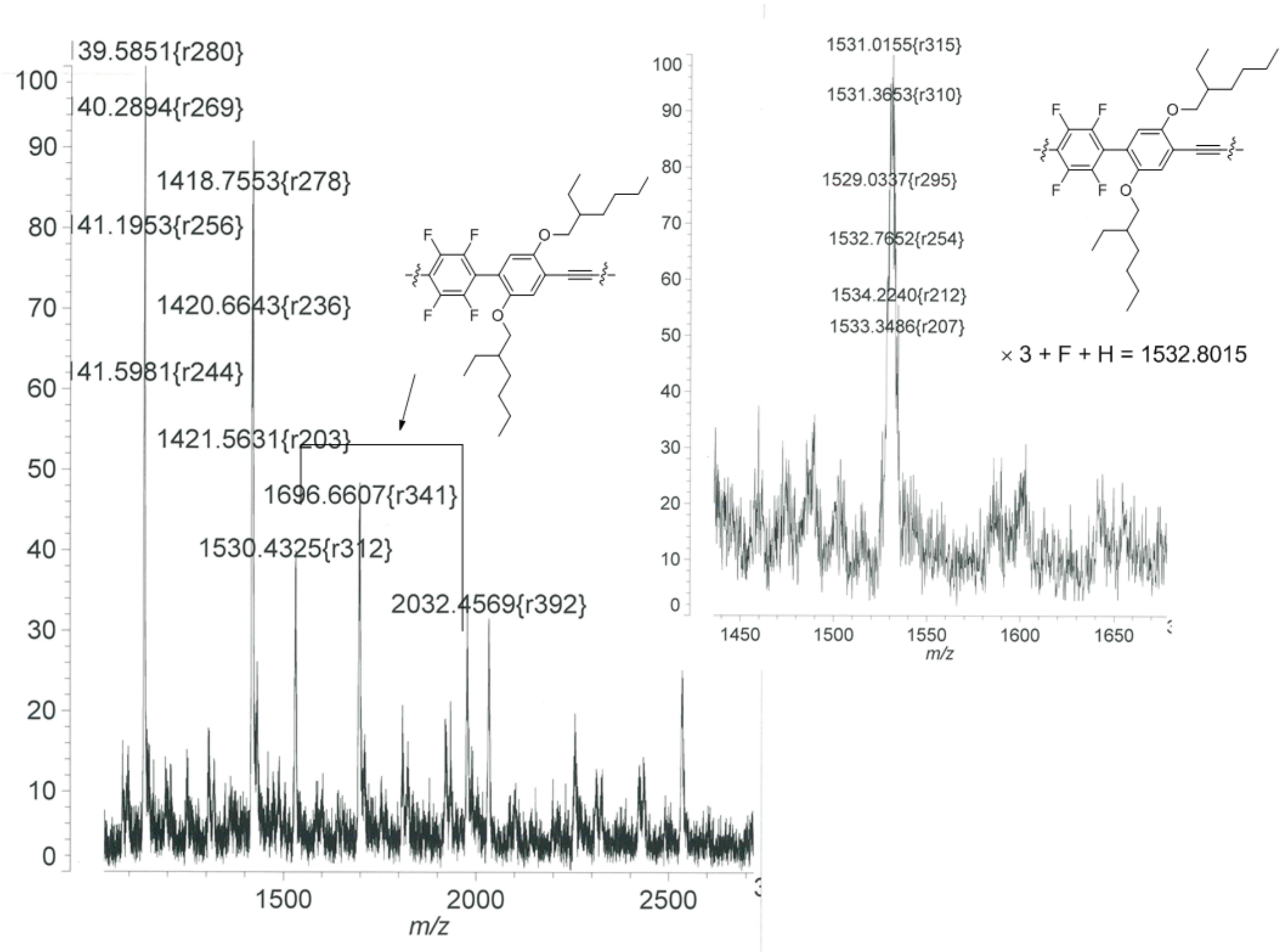

Fig. S2 MALDI-TOF mass spectra of **2**.

Studies on M_n and PDI as function of monomer conversion in the polymerization of 1

A stock solution of **1** (0.60 g, 1.00 mmol) and fluorobenzene (0.31 g, 3.23 mmol) in THF (20 mL) was prepared. To a 2 mL of the solution was added 2.5 mol % of TBAF. The reaction was terminated at 5, 10, 20, 40, 90, and 180 s, respectively, by addition of 0.2 mL of ethanol. To the reaction mixture was added 2 mL of $CDCl_3$. The conversion of **1** was estimated by ^{19}F NMR analysis using fluorobenzene as an internal standard. The M_n and PDI were estimated by SEC analysis with polystyrene standards. The results are shown in Table S1.

Table S1 M_n and PDI as function of monomer conversion in the polymerization of **1**[a]

Time/s	Conversion of **1**/%[a]	M_n[b]	PDI
0	0	766	1.02
5	32	1639	1.13
7	40	1809	1.24
40	46	2087	1.41
180	62	3101	1.68

[a] Determined by the integral ratio in the ^{19}F NMR with fluorobenzene as an internal standard.
[b] Determined by SEC with polystyrene standards.

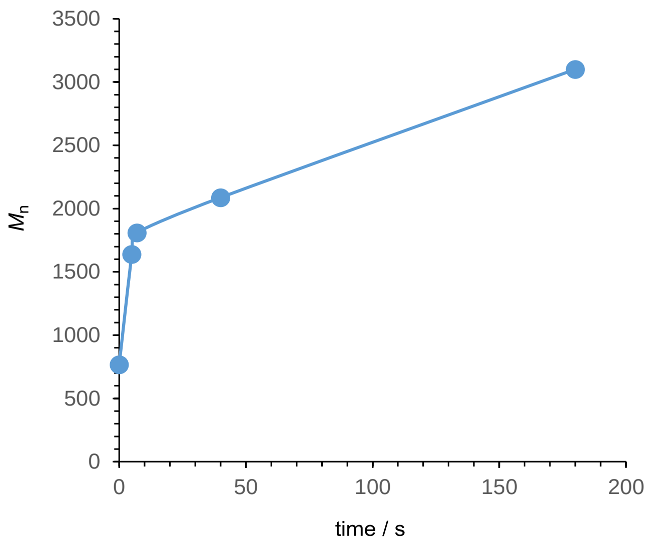

Fig. S3 M_n as function of time in the polymerization of **1**.

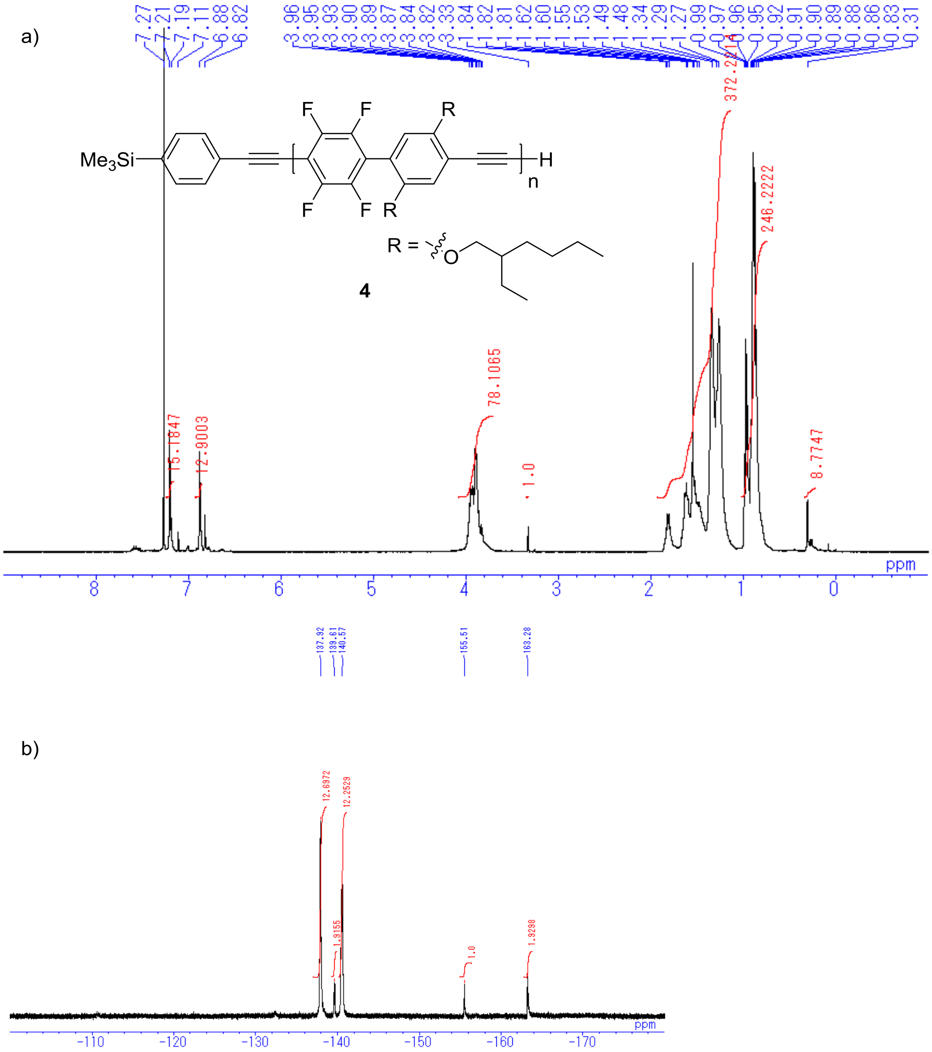

Fig. S4 a) ^{1}H and b) ^{19}F NMR spectra of **4** prepared by the polymerization of **1** with 1-(4-trimethylsilyl)phenyl-2-(triethoxysilyl)acetylene **3**/K^{t}OBu/cryptand[2.2.2] ($CDCl_3$).

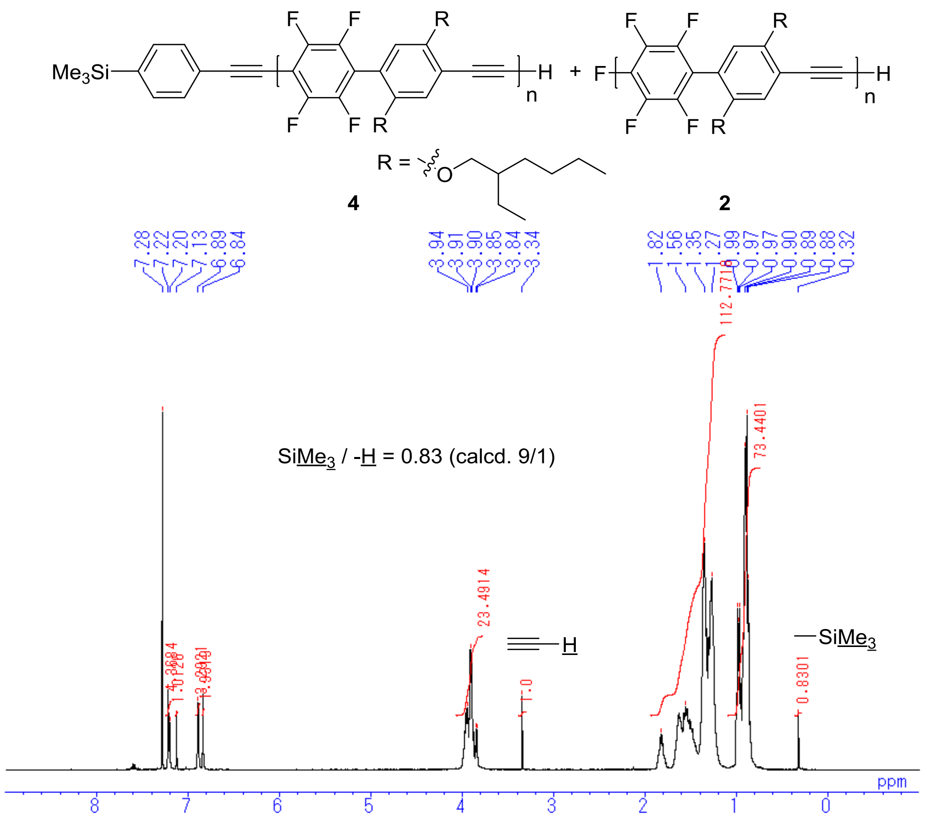

Fig. S5 ^{1}H NMR spectrum of the polymer initiated with 1-trimethylsilyl-4-(trimethylsilylethynyl)benzene/TBAF in the polymerization of **1** ($CDCl_3$).

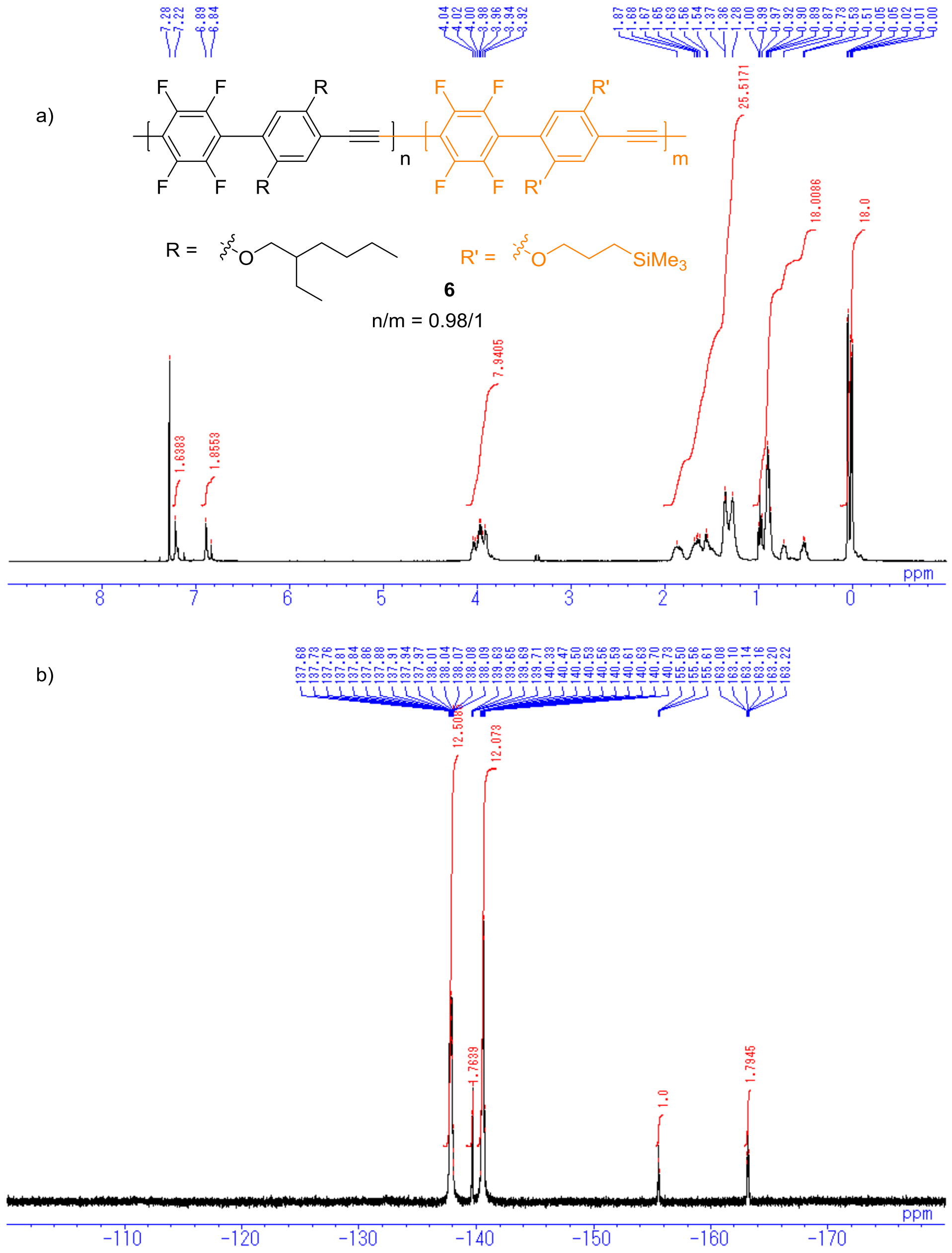

Fig. S6 a) ^{1}H and b) ^{19}F NMR spectra of **6** ($CDCl_3$).

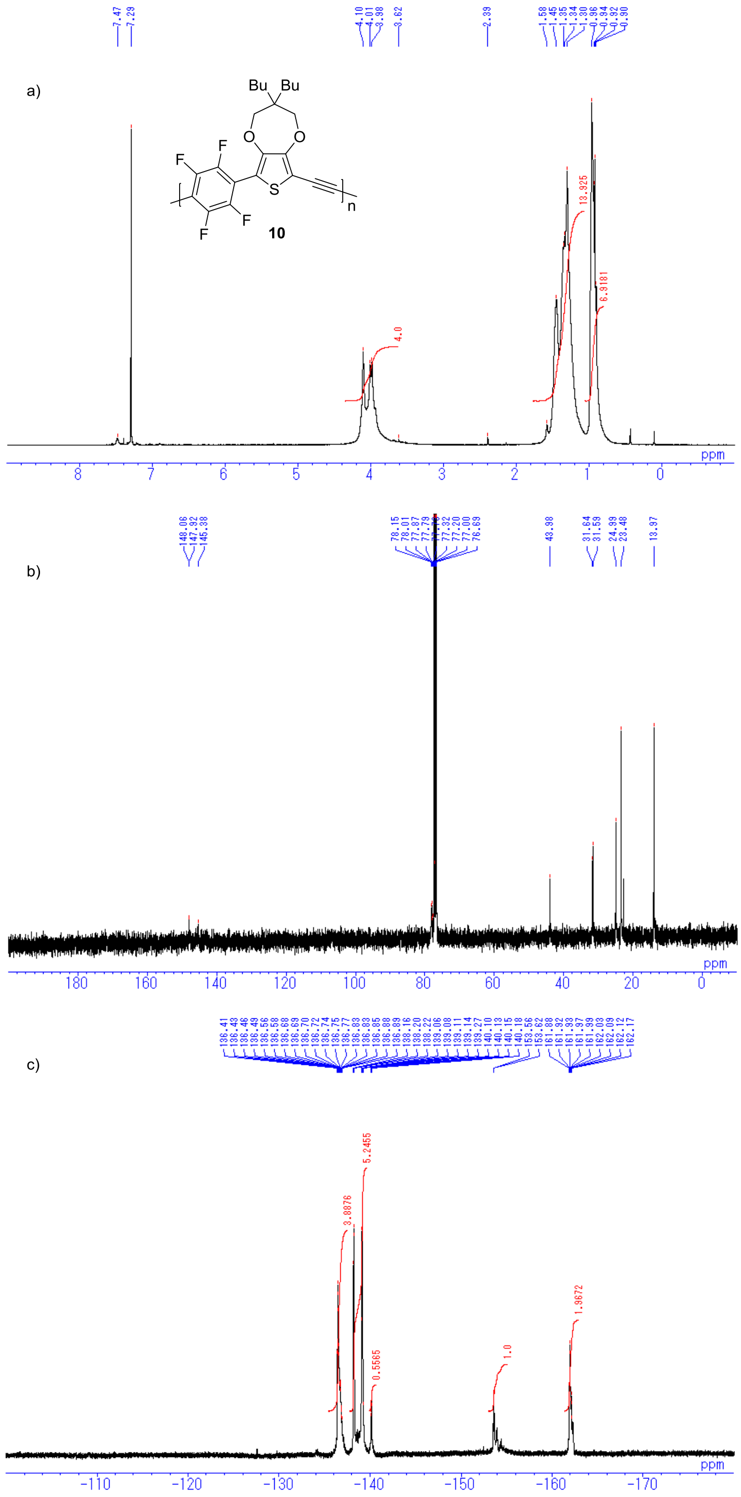

Fig. S7 a) ^{1}H, b) ^{13}C, and c) ^{19}F NMR spectra of **10** ($CDCl_3$)

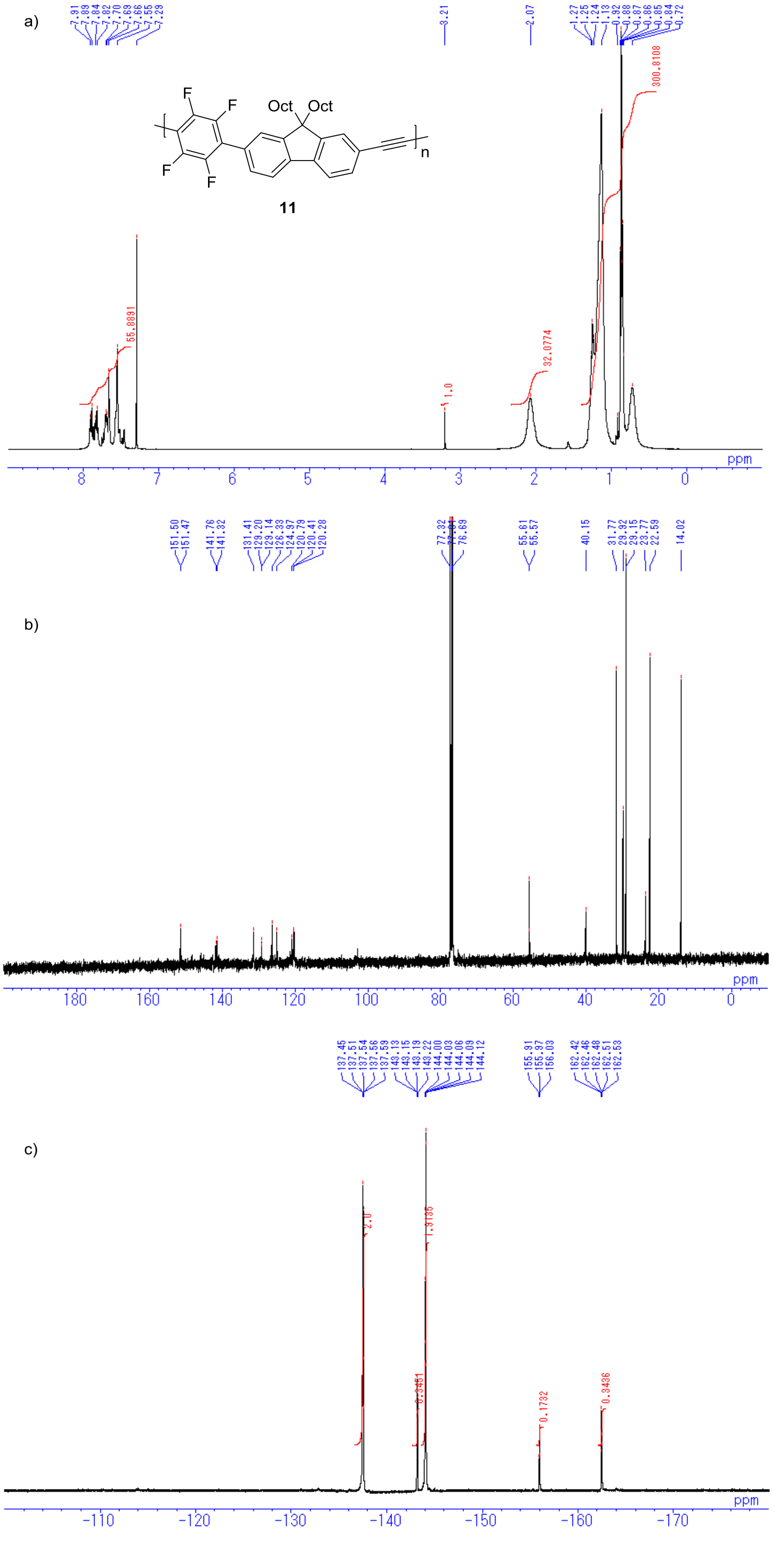

Fig. S8 a) ^{1}H, b) ^{13}C, and c) ^{19}F NMR spectra of **11** ($CDCl_3$).

a)

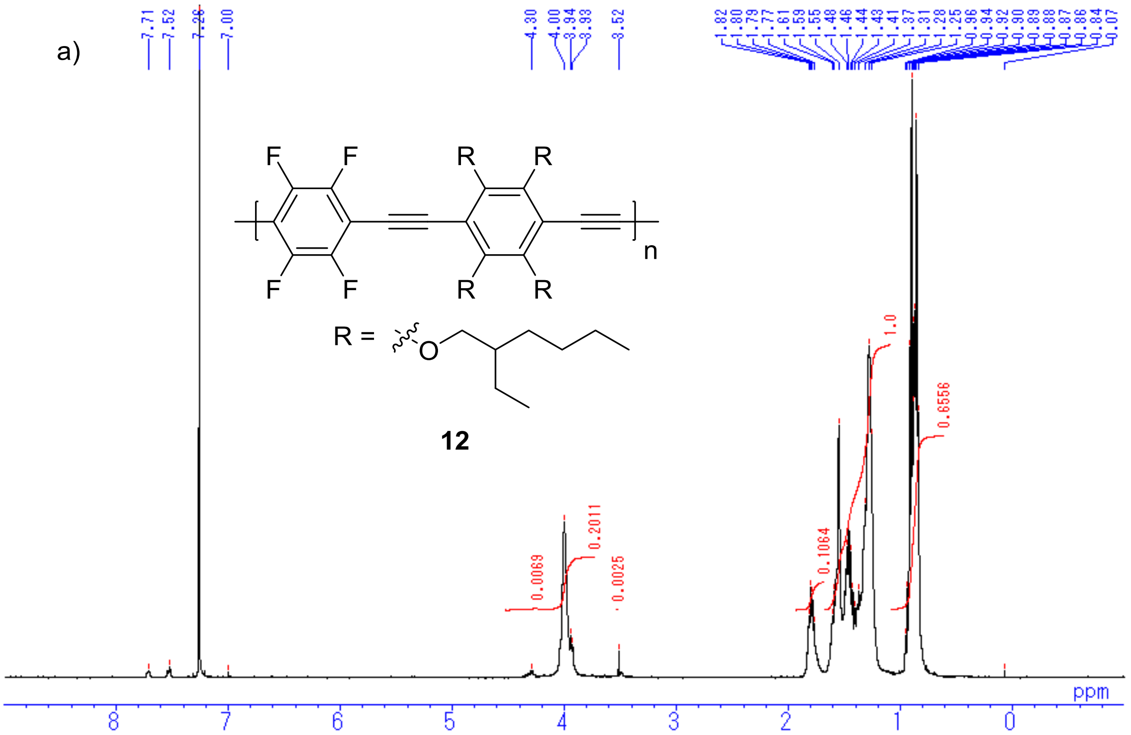

b)

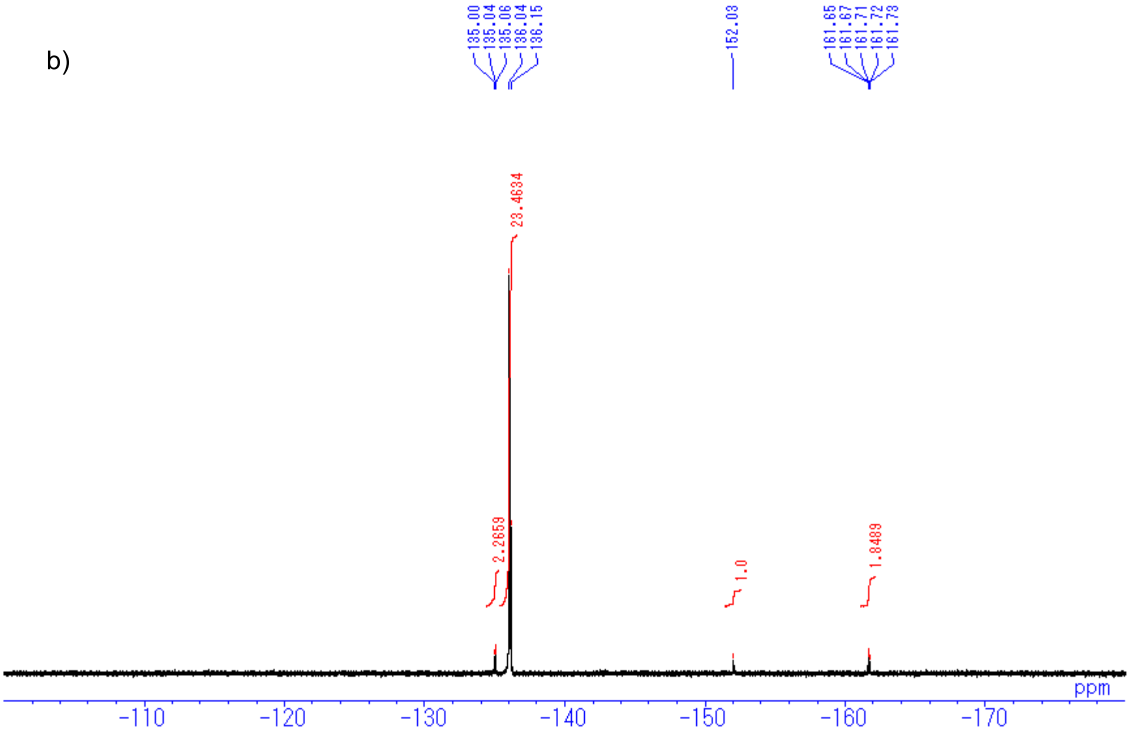

Fig. S9 a) ^{1}H, b) ^{13}C, and c) ^{19}F NMR spectra of **12** ($CDCl_3$).

--

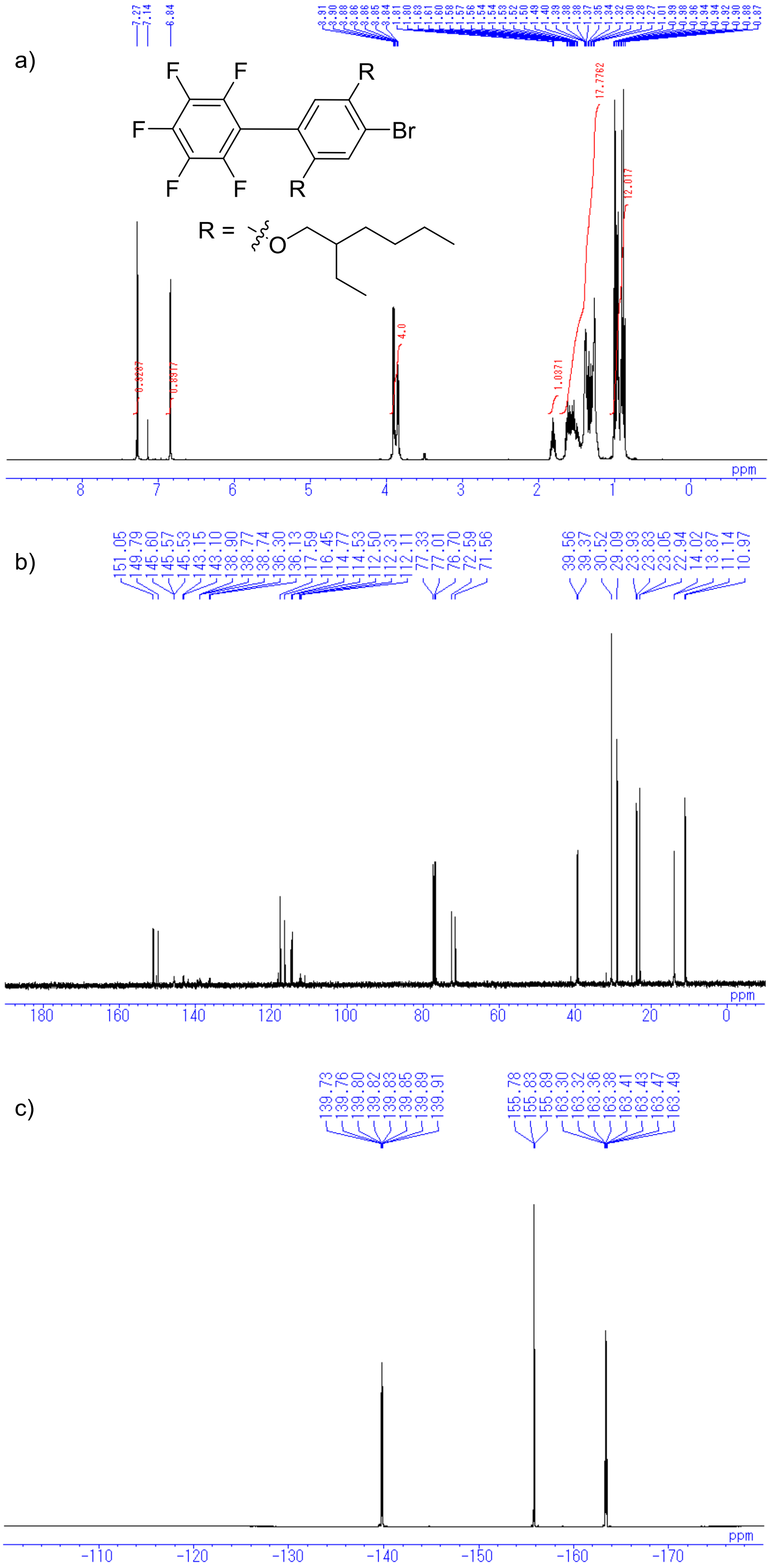

(a) ^{1}H, b) ^{13}C, and c) ^{19}F NMR spectra of
2-bromo-1,4-di(2-ethylhexyloxy)-3-pentafluorophenylbenzene ($CDCl_3$)

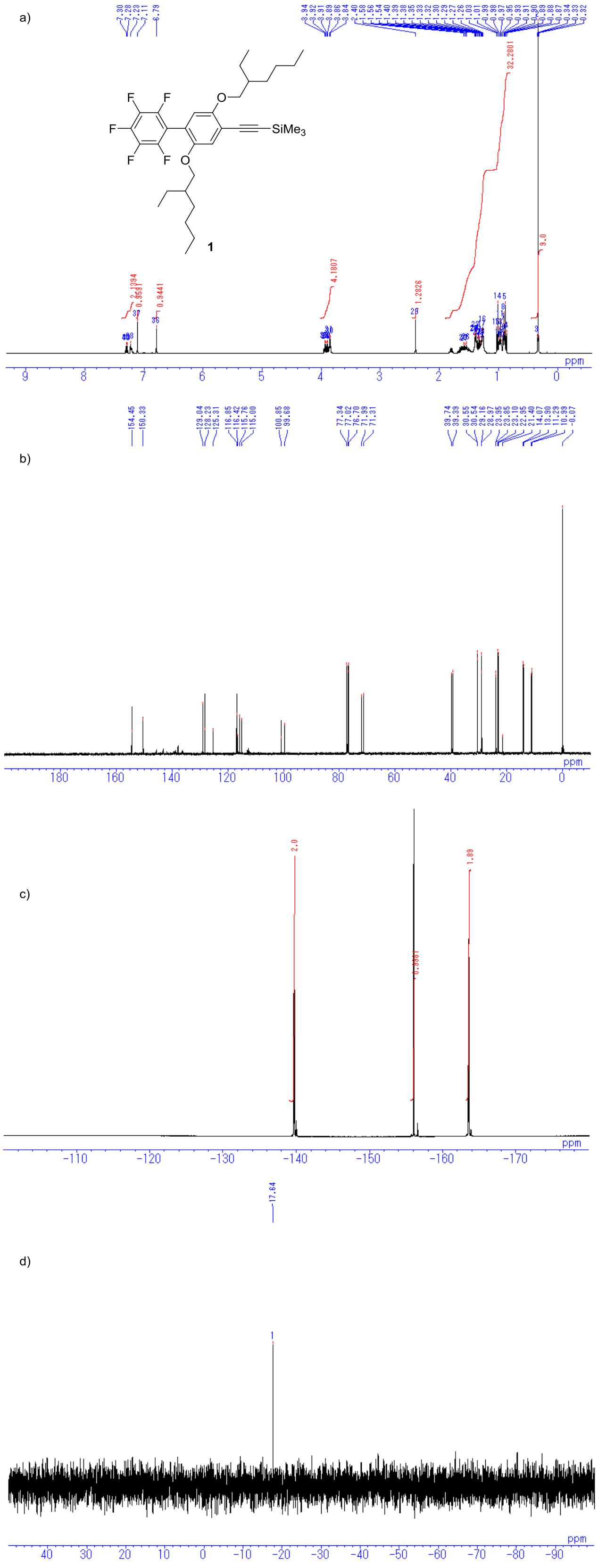

(a)^{1}H, (b) ^{13}C, (c) ^{19}F, and ^{29}Si NMR spectra of **1** ($CDCl_3$).

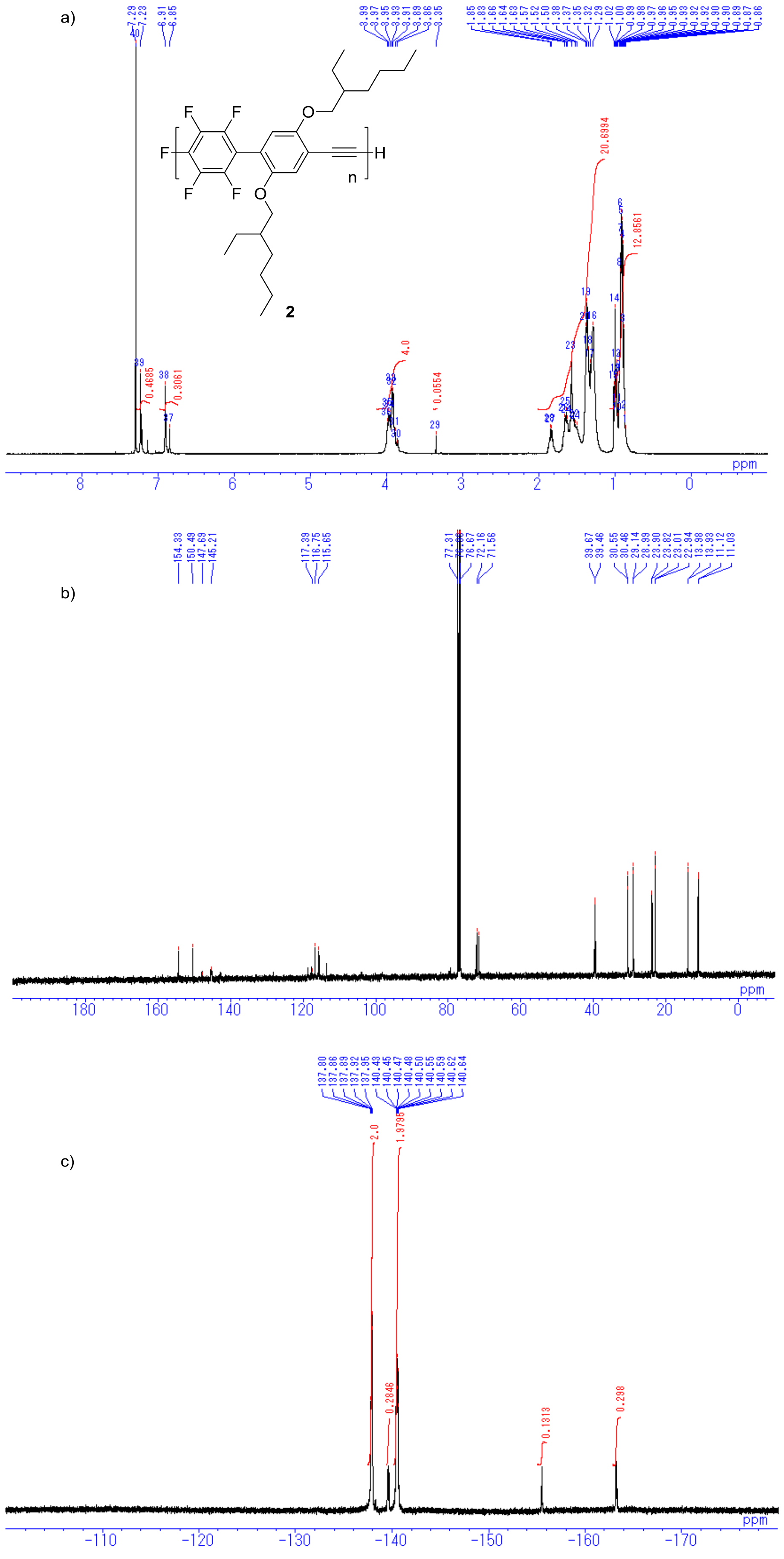

a) ^{1}H, b) ^{13}C, and c) ^{19}F NMR spectra of **2** ($CDCl_3$)

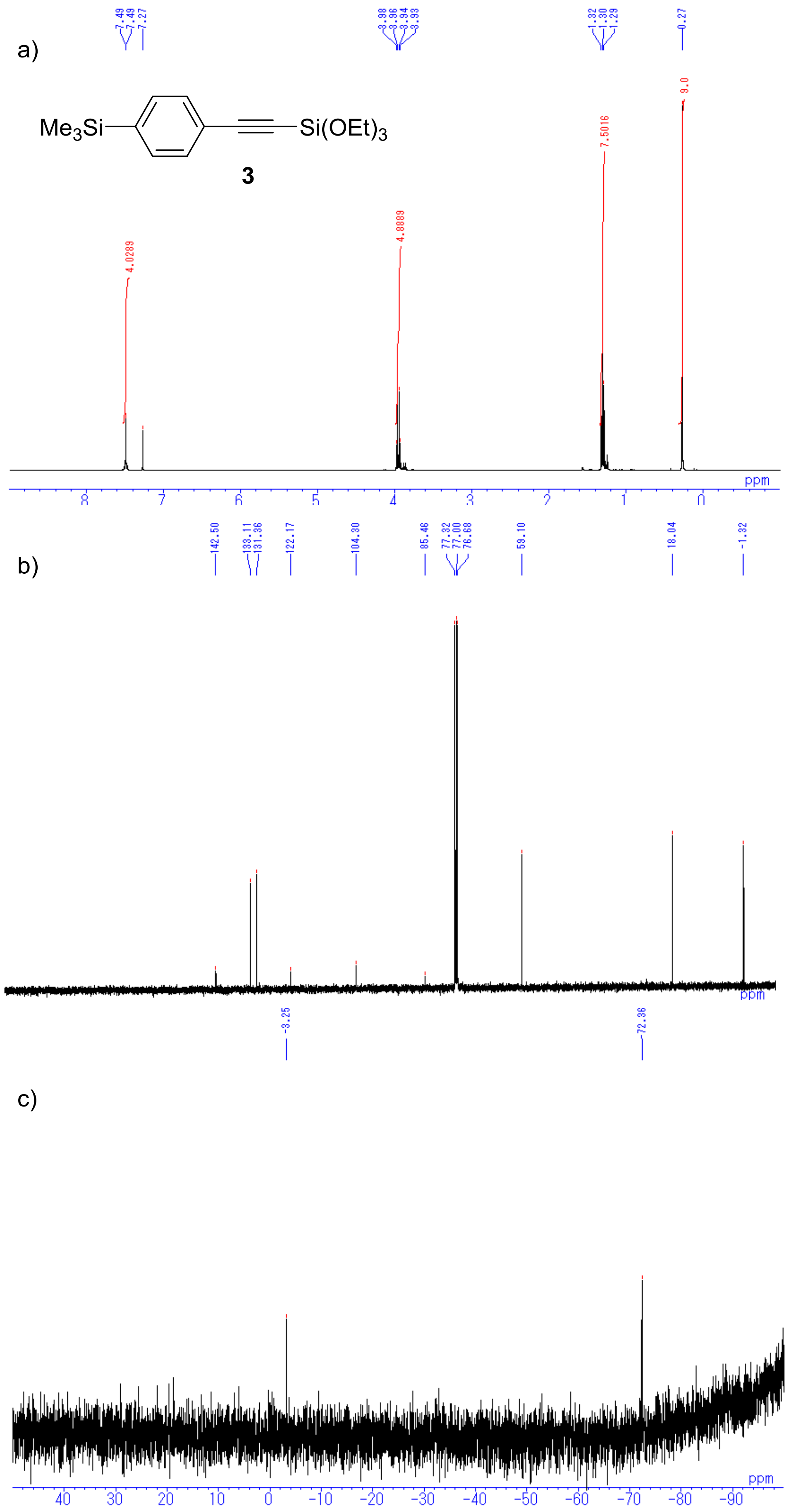

(a) ^{1}H, (b) ^{13}C, (c) ^{19}F NMR spectra of **3** ($CDCl_3$)

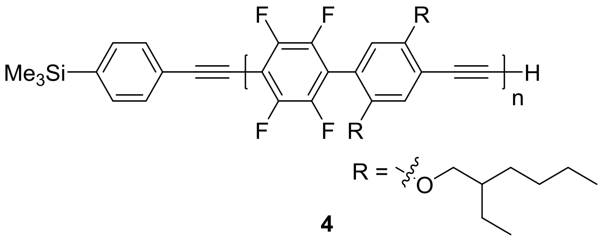

4

a)

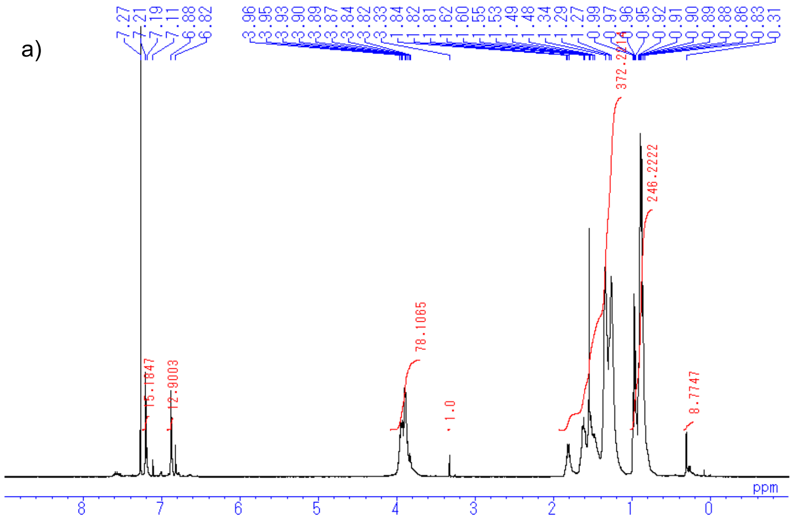

b)

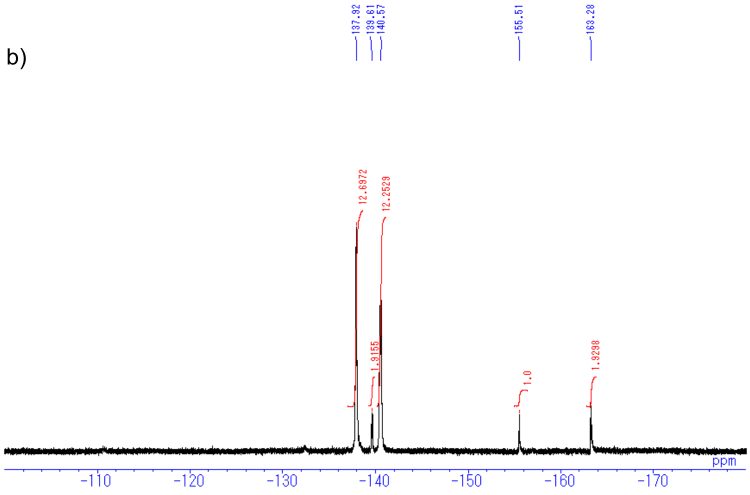

(a) ^{1}H and (b) ^{19}F NMR spectra of **4** ($CDCl_3$)

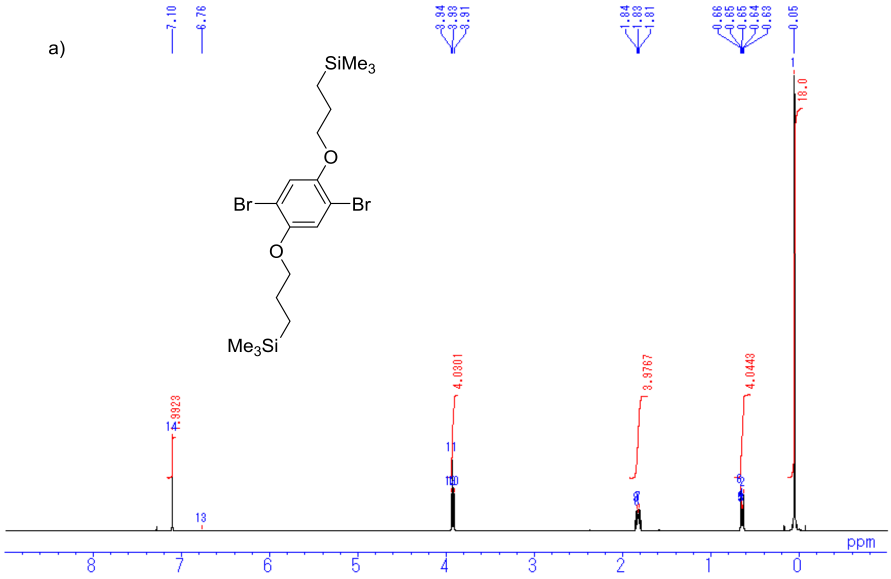

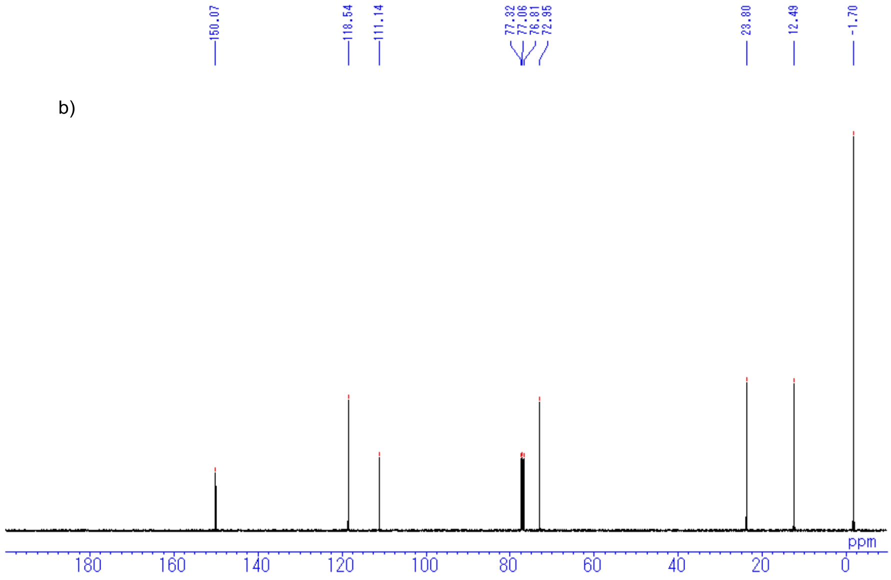

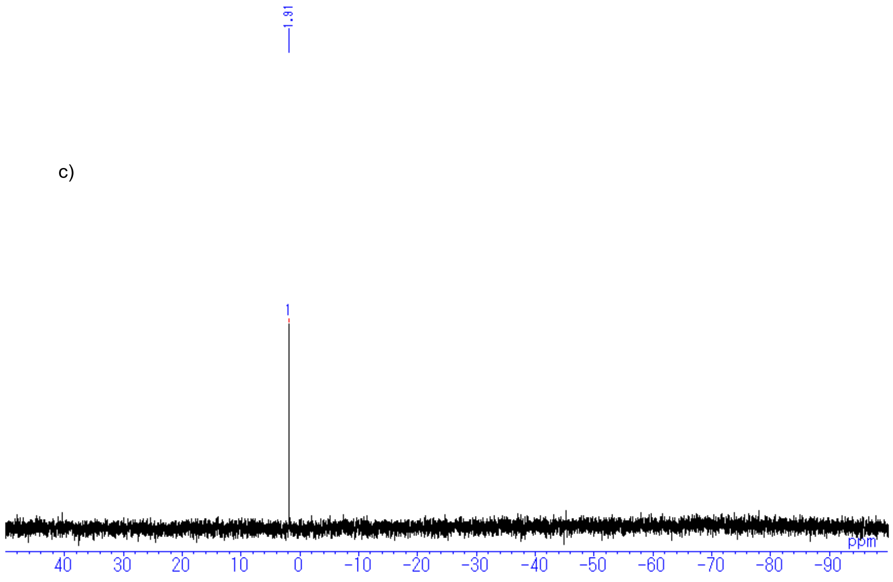

(a) ^{1}H, (b) ^{13}C, and (c) ^{29}Si NMR spectra of 2,5-dibromo-1,4-bis(3'-trimethylsilylpropoxy)benzene ($CDCl_3$)

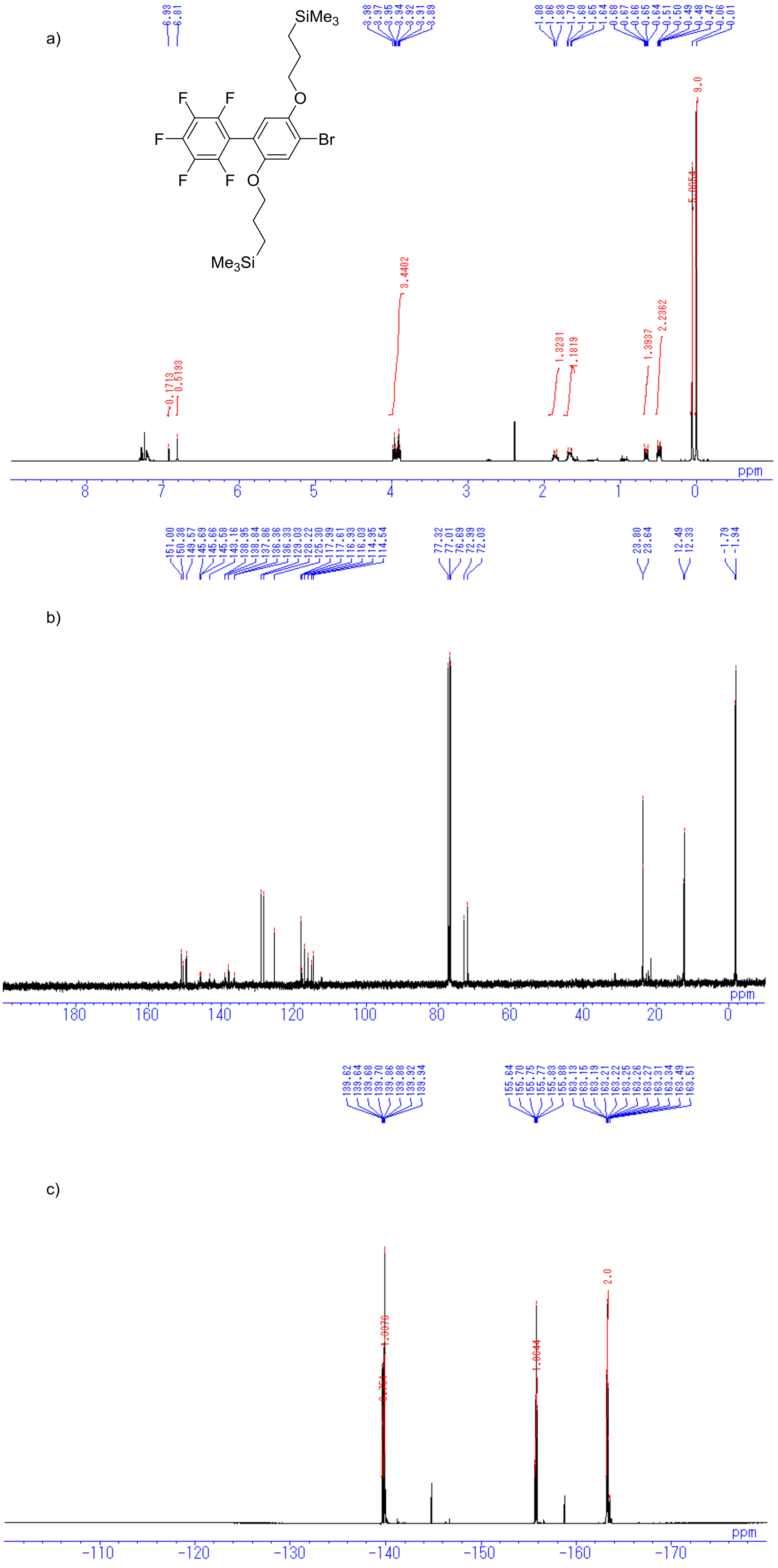

(a) ^{1}H, (b) ^{13}C, and (c) ^{29}Si NMR spectra of 2-bromo-1,4-bis(3'-trimethylsilylpropoxy)-5-pentafluorophenylbenzene ($CDCl_3$)

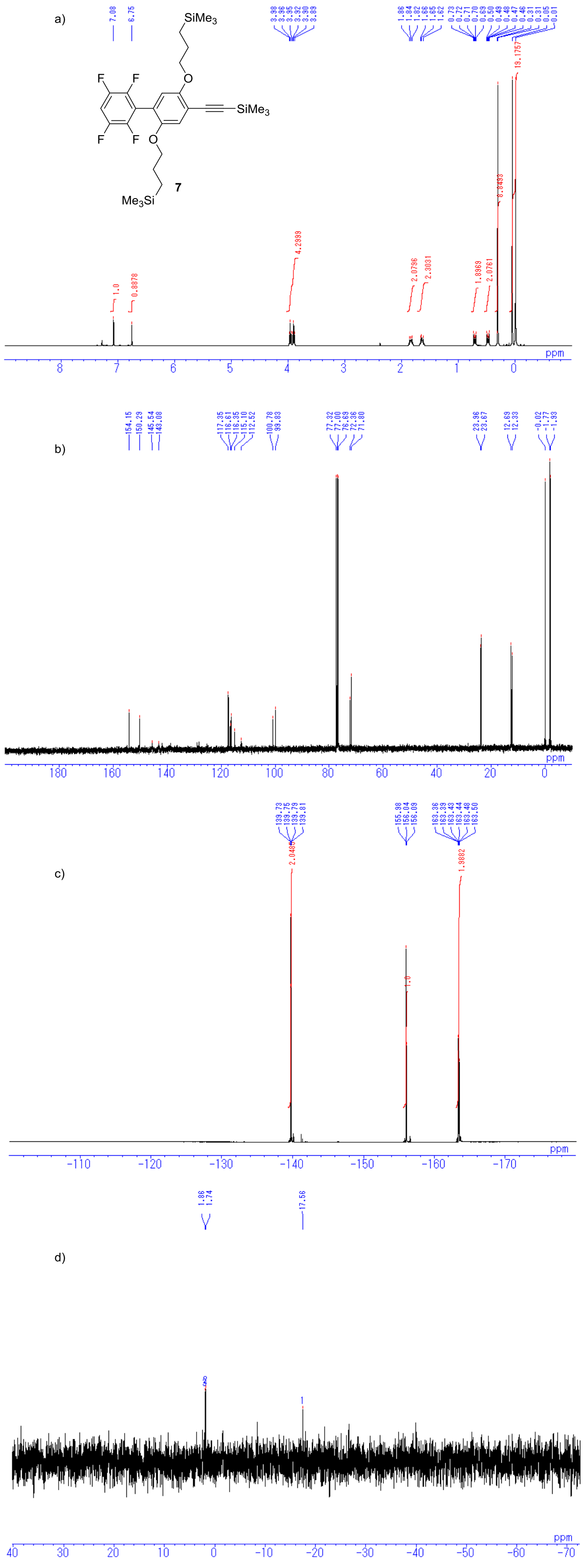

(a) ^{1}H, (b) ^{13}C, (c) ^{19}F, and (d) ^{29}Si NMR spectra of 1,4-bis(3’-trimethylsilylpropoxy)-2-pentafluorophenyl-5-(trimethylsilylethynyl)benzene **7** ($CDCl_3$)

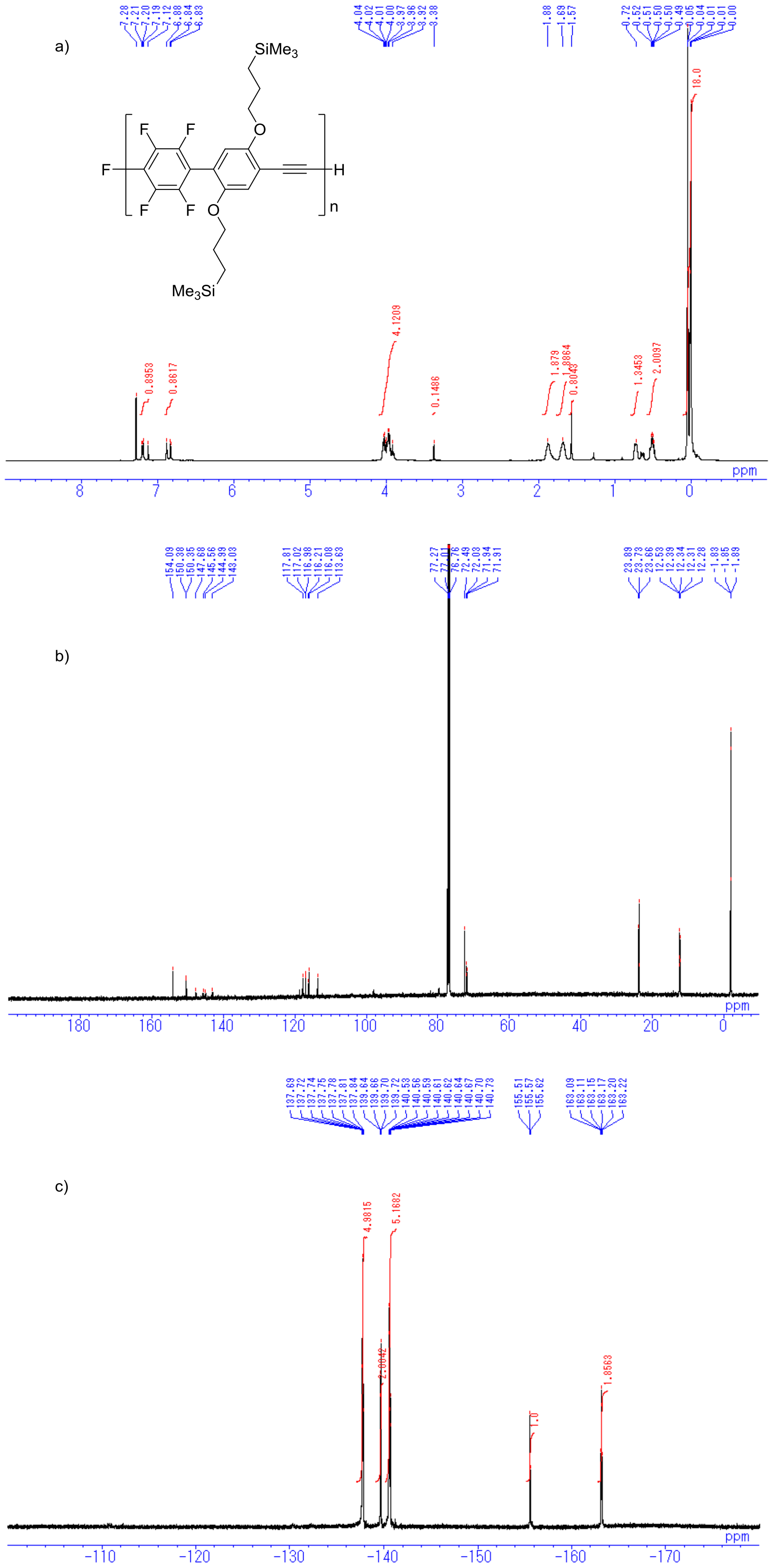

(a) ^{1}H, (b) ^{13}C, and (c) ^{19}F NMR spectra of the polymer prepared by the polymerization of **7**, poly(1,4-tetrafluorophenylene-1,4-bis(3’-trimethylsilylpropoxy)phenylene-thieneylene) ($CDCl_3$)

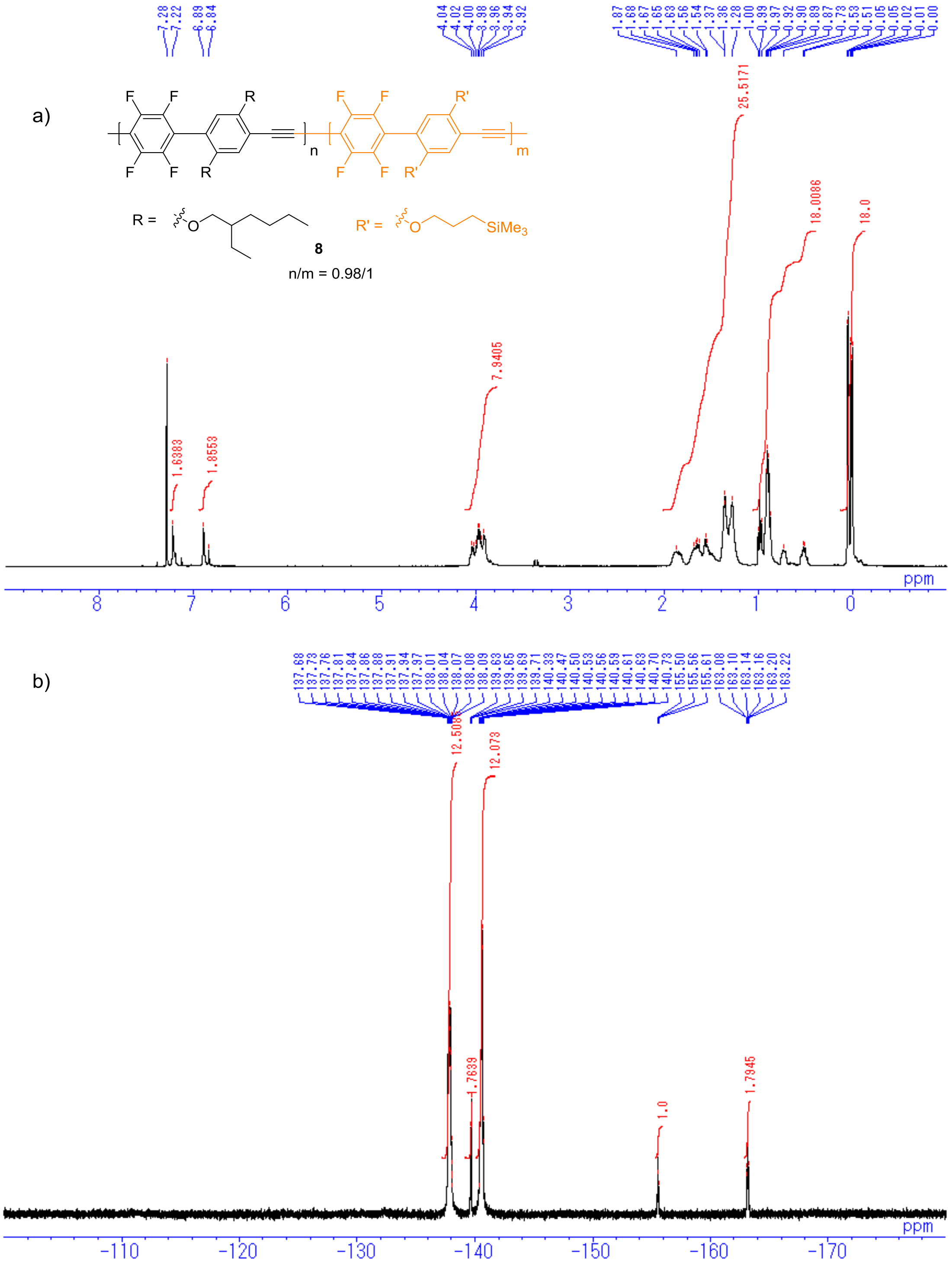

a) ^{1}H and ^{19}F NMR spectra of **8** ($CDCl_3$)

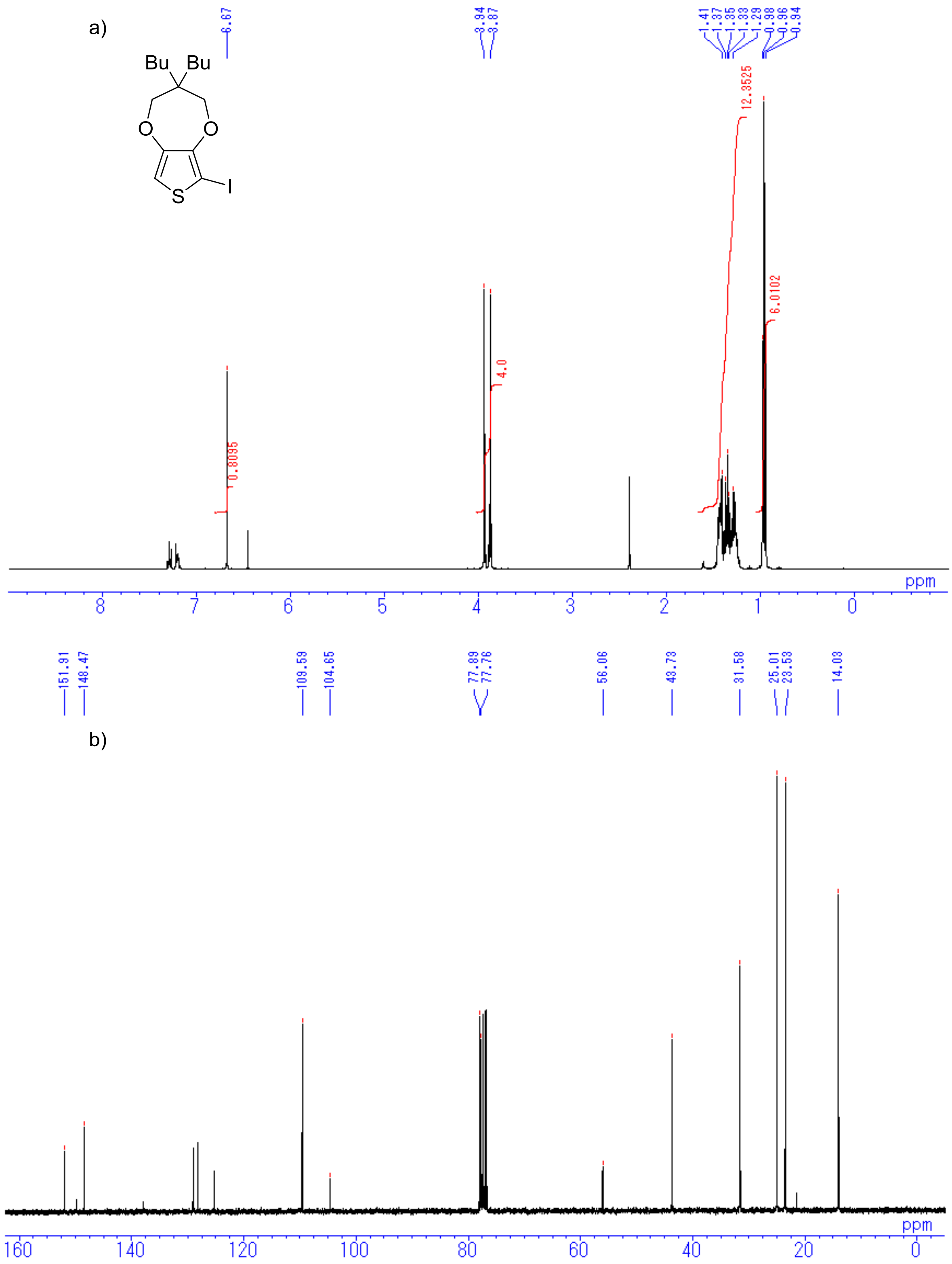

(a) ¹H and (b) ¹³C NMR spectra of 2-iodo-3,4-(2′,2′-dibutylpropylenedioxy)thiophene ($CDCl_3$)

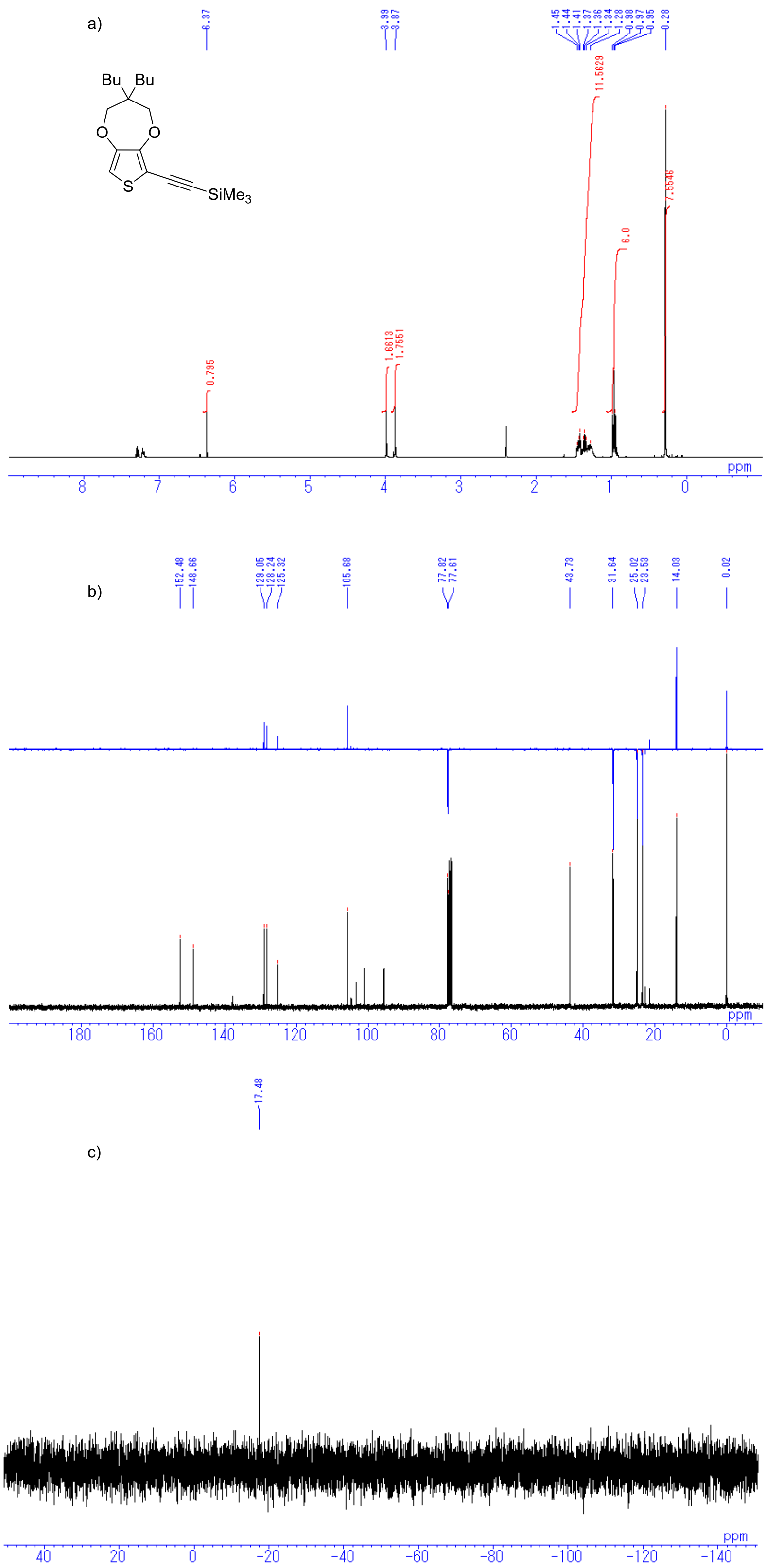

a) ^{1}H, b) ^{13}C, ^{29}Si NMR spectra of
2-trimethylsilylethynyl-3,4-(2′,2′-dibutylpropylenedioxy)thiophene ($CDCl_3$)

a)

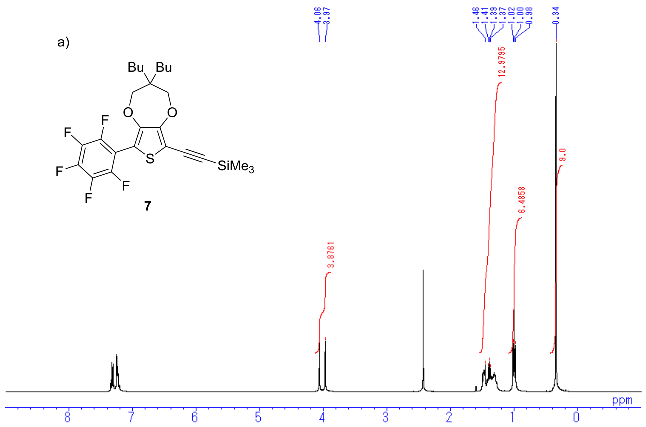

b)

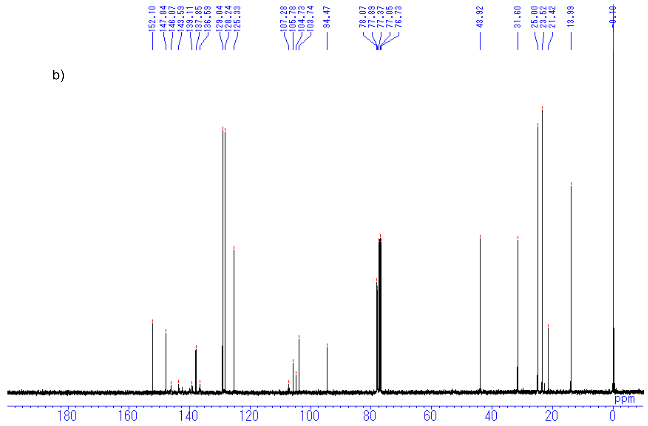

c)

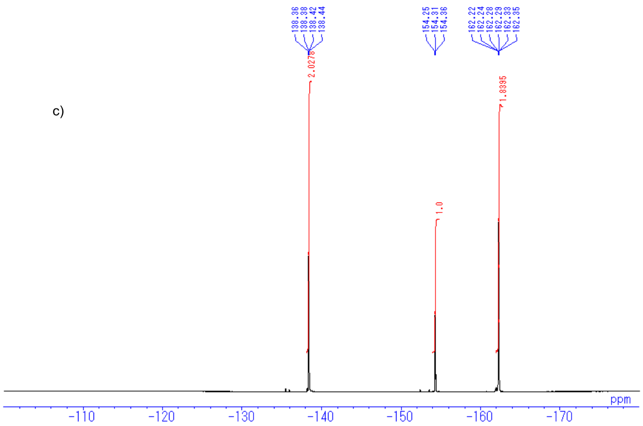

e)

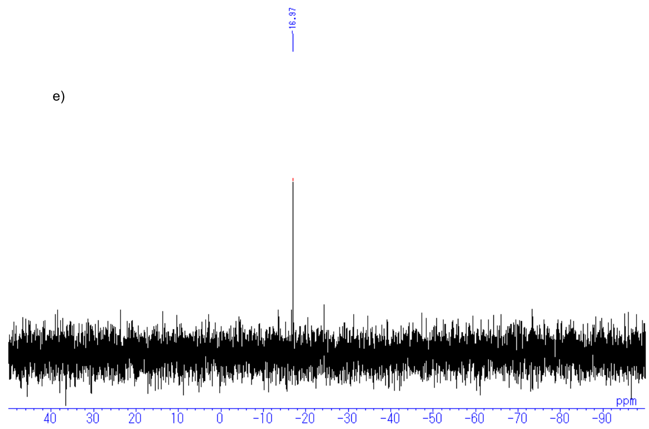

a) ^{1}H, ^{13}C, ^{19}F, and d) ^{29}Si NMR spectra of **7** ($CDCl_3$)

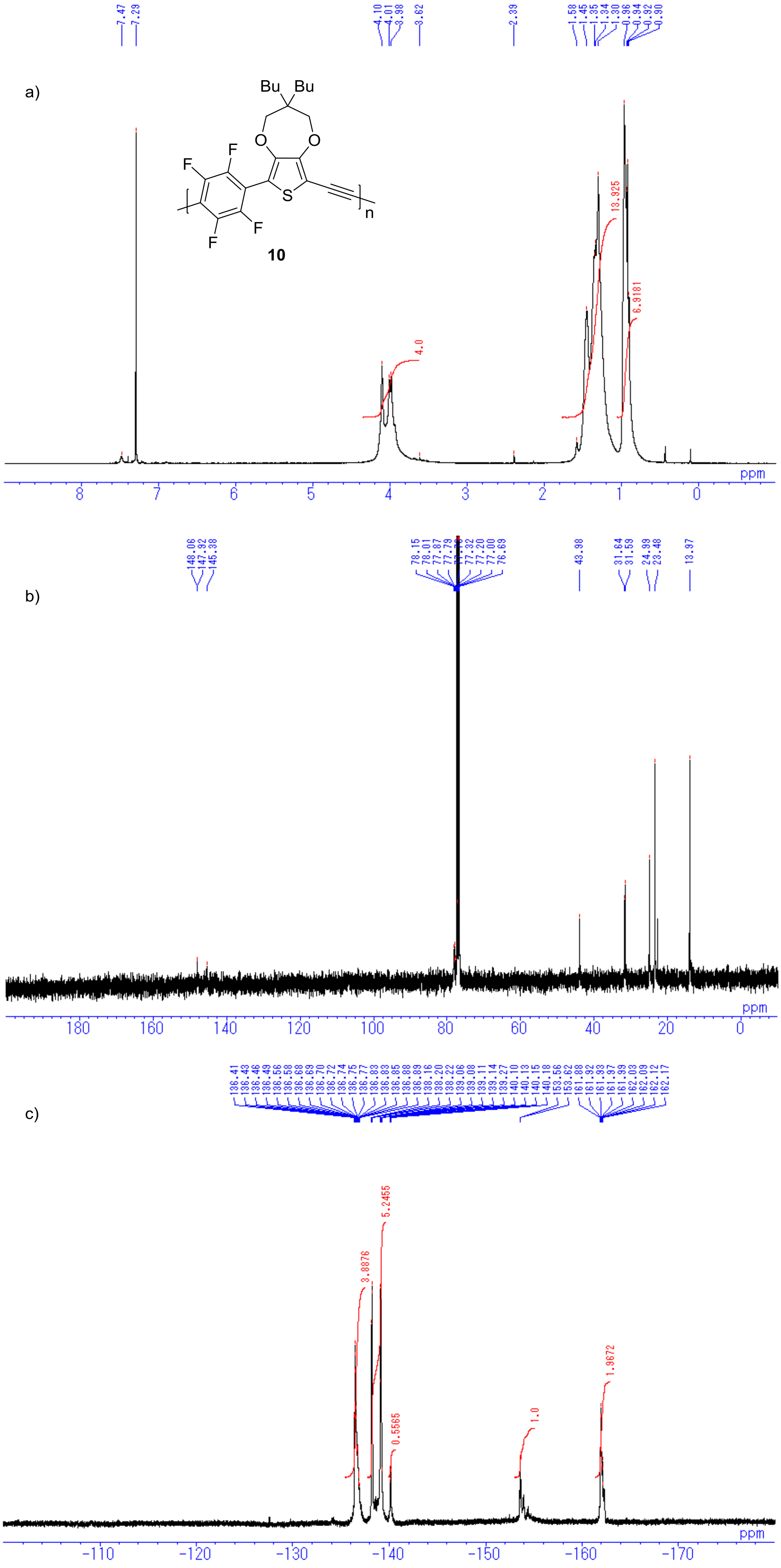

a) ^{1}H, b) ^{13}C, and c) ^{19}F NMR spectra of **10** ($CDCl_3$)

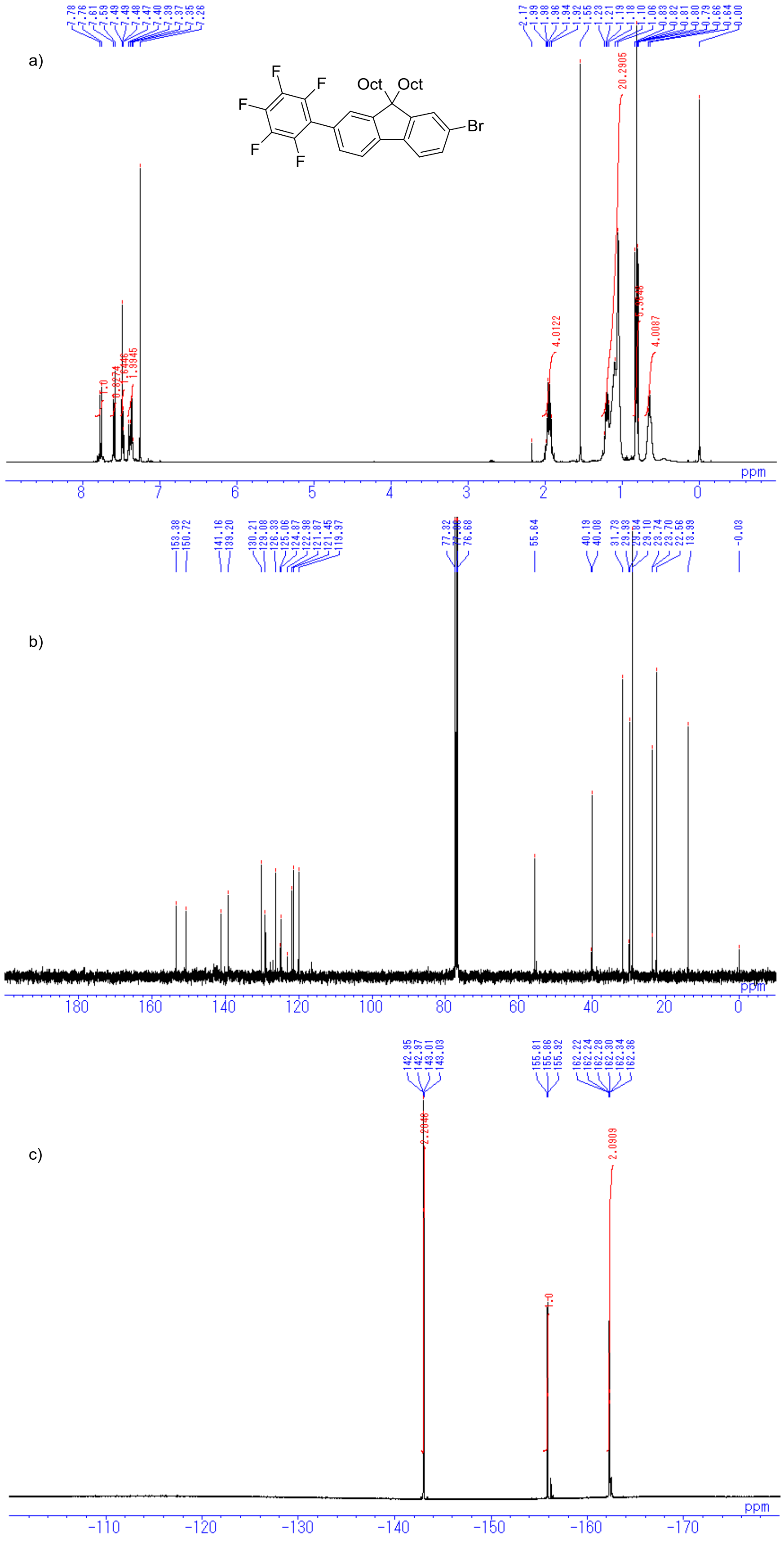

a) ^{1}H, b) ^{13}C, and c) ^{19}F NMR spectra of 2-bromo-7-pentafluorophenyl-9,9-di(*n*-octyl)fluorene ($CDCl_3$)

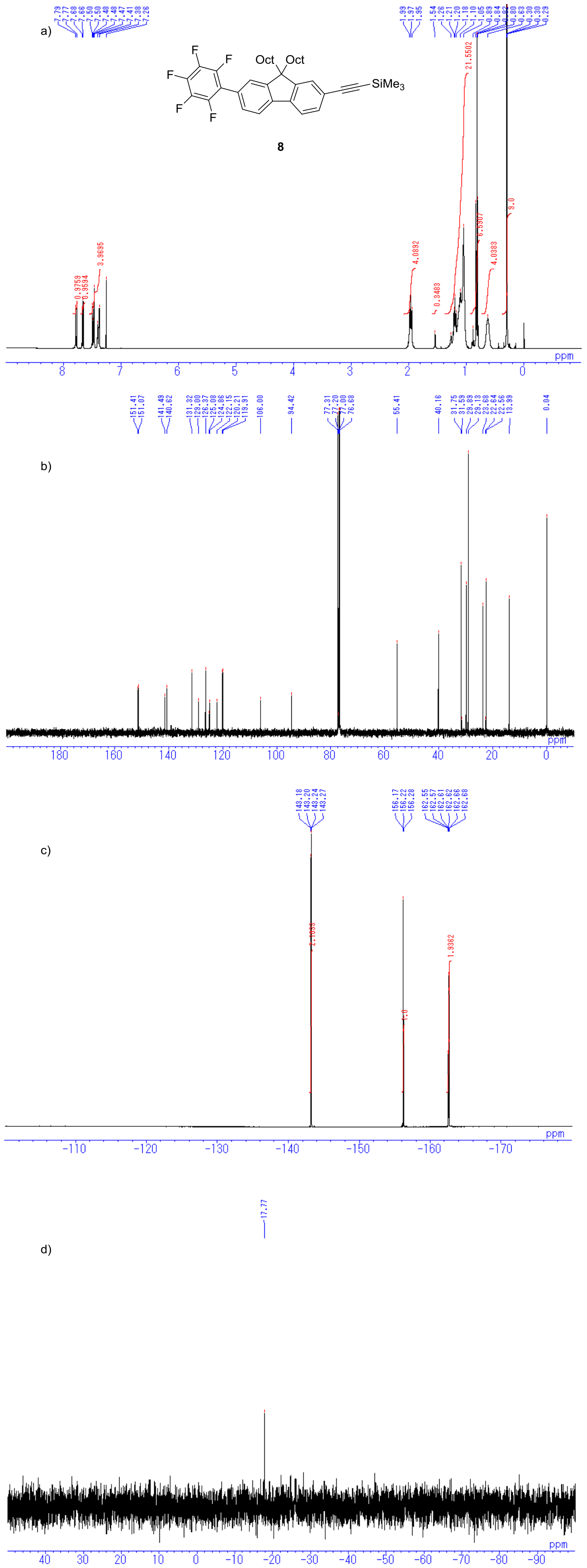

a) ^{1}H, b) ^{13}C, c) ^{19}F, and d) ^{29}Si NMR spectra of **8** ($CDCl_3$)

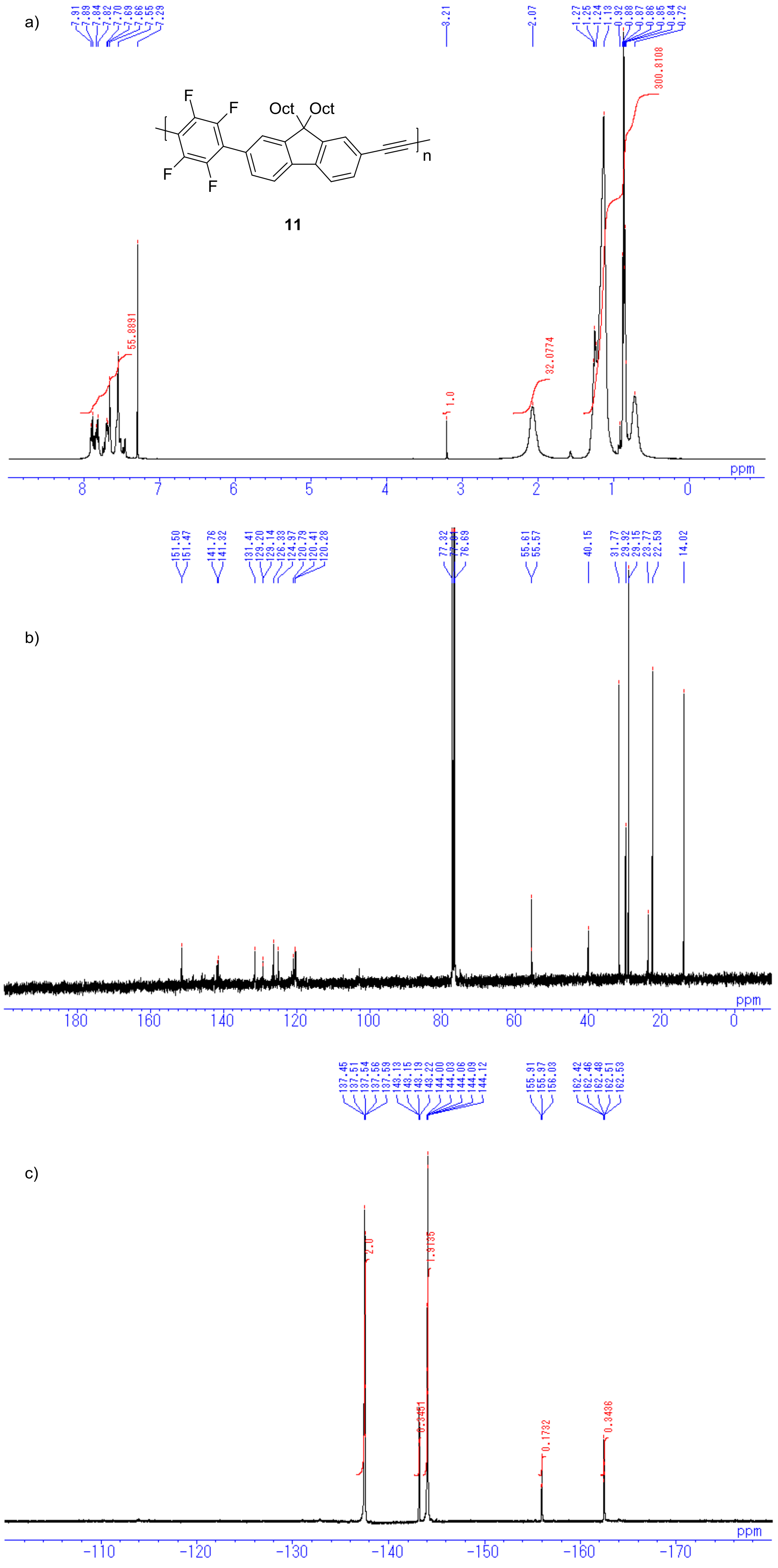

a) ^{1}H, b) ^{13}C, and c) ^{19}F spectra of **11** ($CDCl_3$)

a)

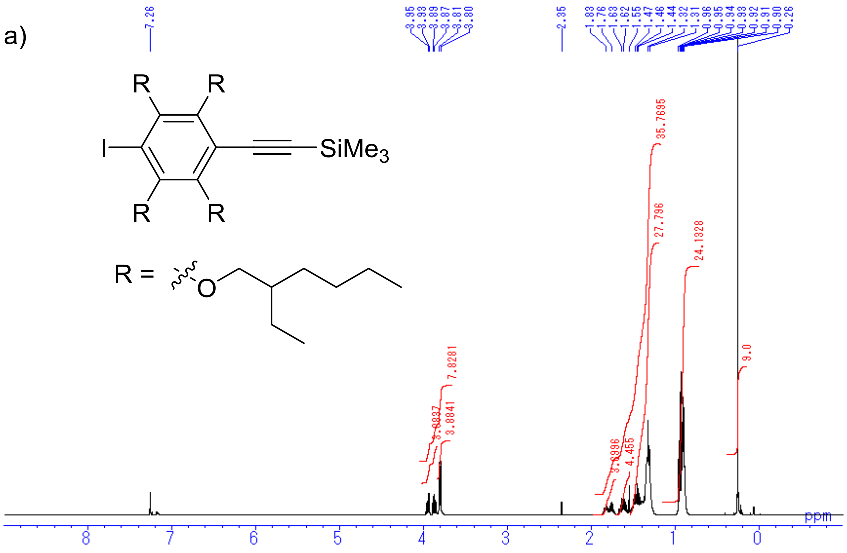

b)

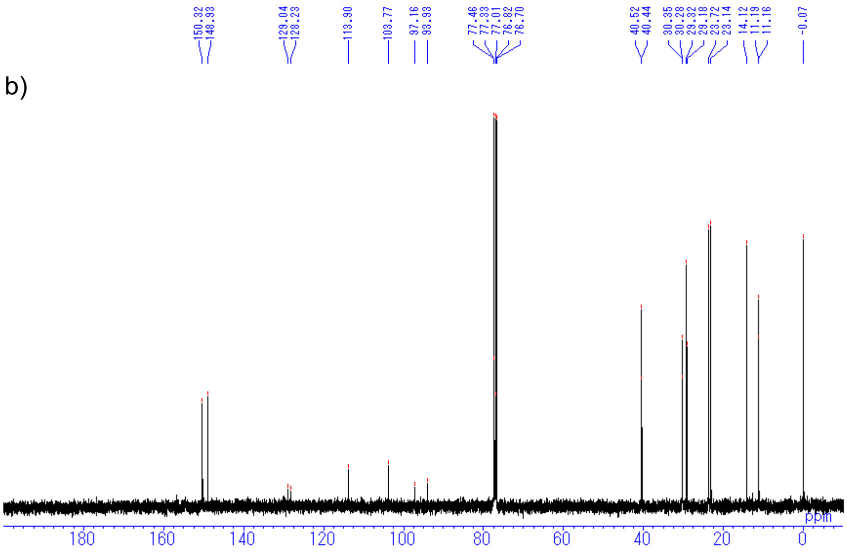

c)

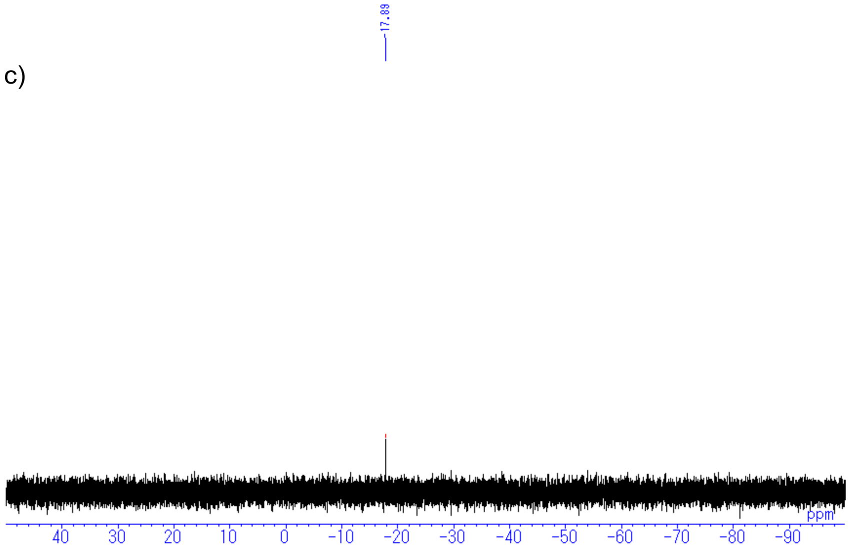

a) ^{1}H, b) ^{13}C, and c) ^{29}Si NMR spectra of 1-trimethylsilylethynyl-2,3,5,6,-tetra(2’-ethylhexyloxy)-4-iodobenzene ($CDCl_3$)

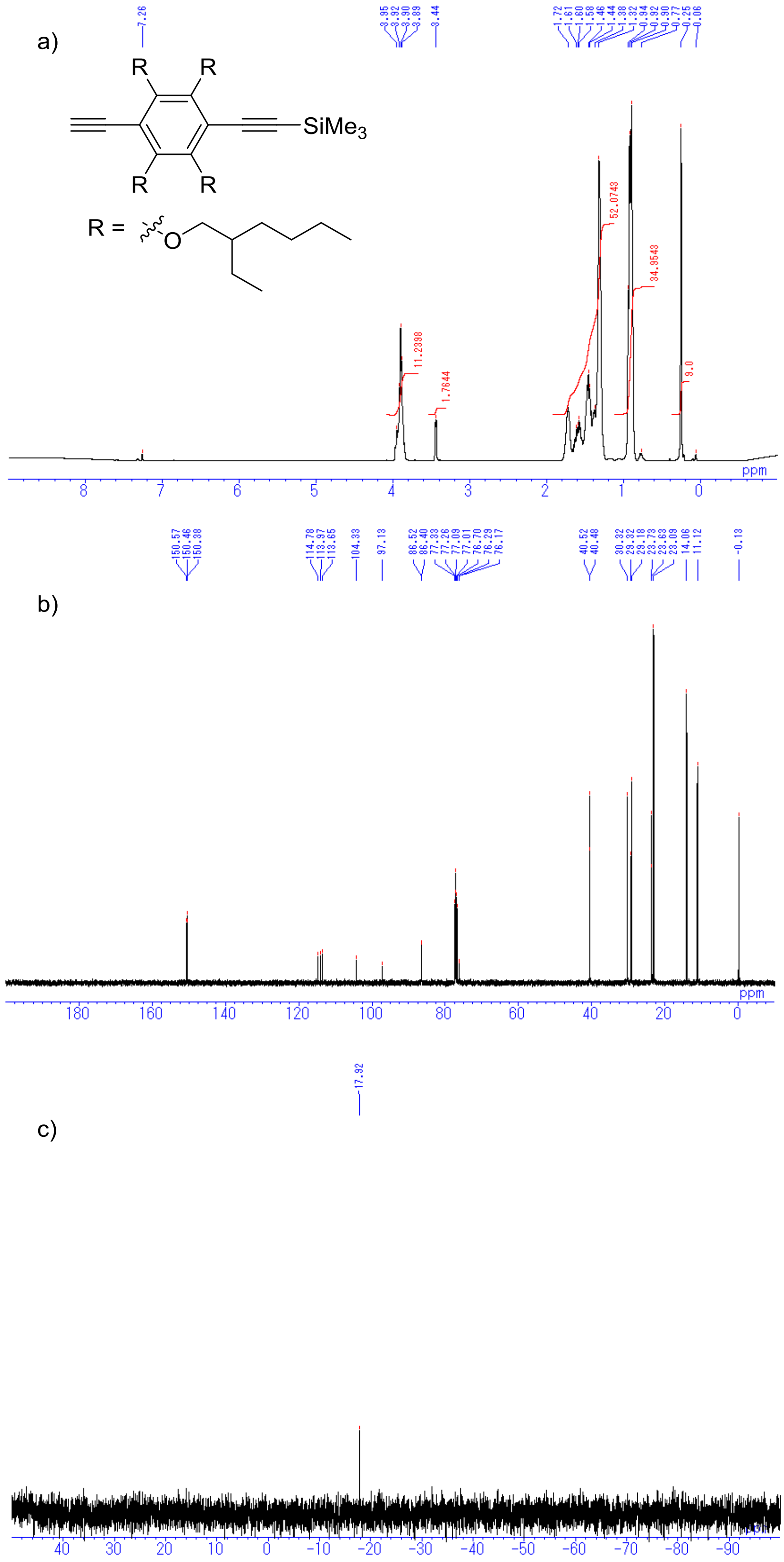

a) ^{1}H, b) ^{13}C, and c) ^{29}Si NMR spectra of 1-trimethylsilylethynyl-2,3,5,6-tetra(2'-ethylhexyloxy)-4-ethynylbenzene ($CDCl_3$)

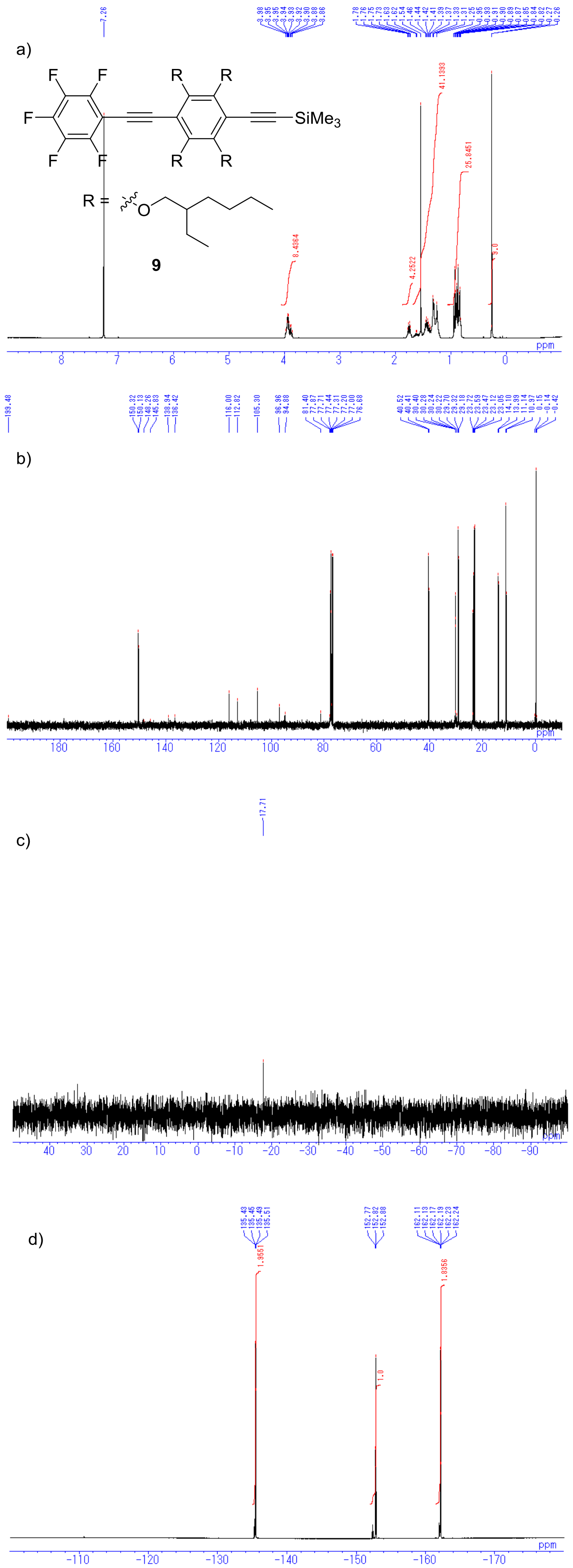

a) ^{1}H, b) ^{13}C, c) ^{29}Si, and d) ^{19}F NMR spectra of **9** ($CDCl_3$)

a)

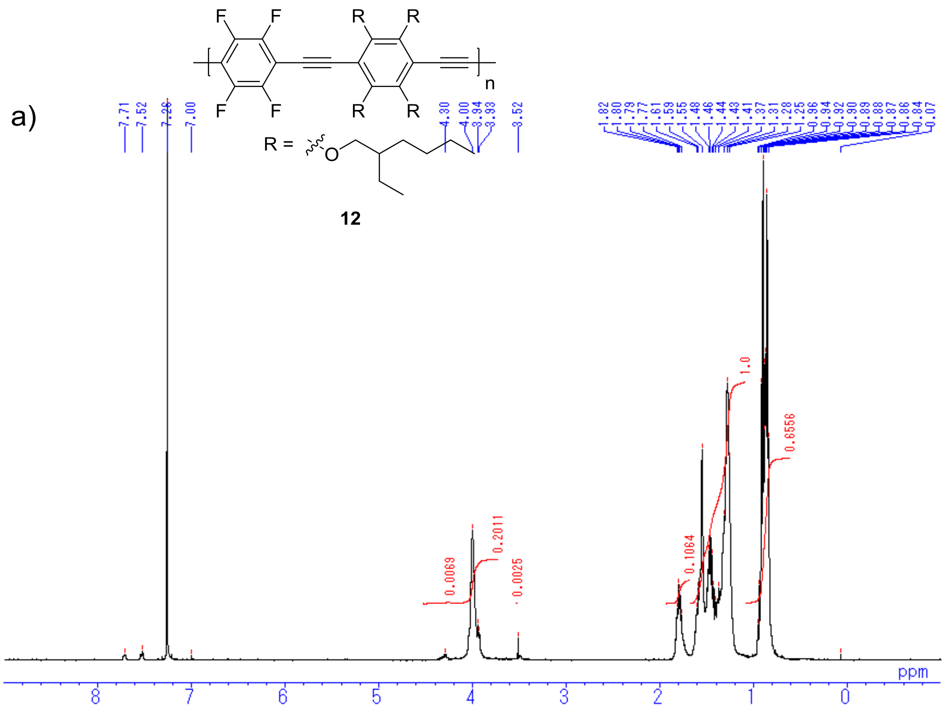

b)

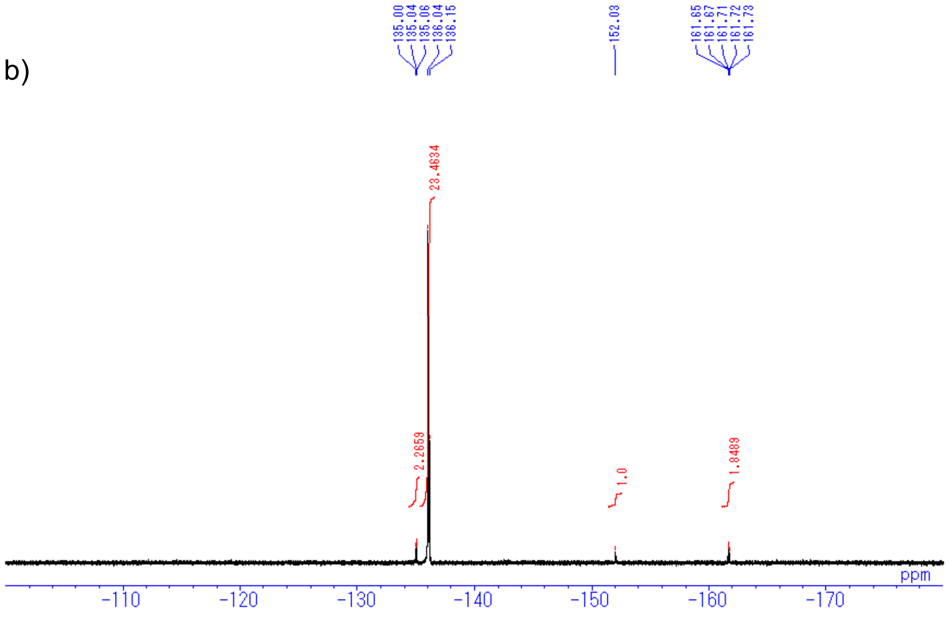

a) ^{1}H and ^{19}F NMR spectra of **12** ($CDCl_3$)
